# Comprehensive ascertainment of bleeding in patients prescribed different combinations of dual antiplatelet therapy (DAPT) and triple therapy (TT) in the UK: study protocol for three population-based cohort studies emulating ‘target trials’ (the ADAPTT Study)

**DOI:** 10.1136/bmjopen-2019-029388

**Published:** 2019-06-04

**Authors:** Maria Pufulete, Jessica Harris, Jonathan A C Sterne, Thomas W Johnson, Daniel Lasserson, Andrew Mumford, Brett Doble, Sarah Wordsworth, Umberto Benedetto, Chris A Rogers, Yoon Loke, Christalla Pithara, Sabi Redwood, Barnaby C Reeves

**Affiliations:** 1 Clinical Trials and Evaluation Unit, Bristol Trials Centre, University of Bristol, Bristol, UK; 2 NIHR Biomedical Research Centre, Department of Population Health Sciences, University of Bristol, Bristol, UK; 3 Department of Cardiology, Bristol Heart Institute, Bristol, UK; 4 Institute of Applied Health Research, University of Birmingham, Birmingham, UK; 5 Bristol Medical School, University of Bristol, Bristol, UK; 6 Health Economics Research Centre, Nuffield Department of Population Health, University of Oxford, Oxford UK; 7 Norwich Medical School, University of East Anglia, Norwich, UK; 8 Ethnography Research Team, National Institute for Health Research Collaboration for Leadership in Applied Health Research and Care West (NIHR CLAHRC West), Bristol, UK

**Keywords:** Dual antiplatelet therapy (DAPT), acute coronary syndrome (ACS), percutaneous coronary intervention (PCI), coronary artery bypass grafting (CABG), bleeding, Clinical Practice Research Datalink (CPRD), Hospital Episode Statistics (HES).

## Abstract

**Introduction:**

‘Real world’ bleeding in patients exposed to different regimens of dual antiplatelet therapy (DAPT) and triple therapy (TT, DAPT plus an anticoagulant) have a clinical and economic impact but have not been previously quantified.

**Methods and analysis:**

We will use linked Clinical Practice Research Datalink (CPRD) and Hospital Episode Statistics (HES) data to assemble populations eligible for three ‘target trials’ in patient groups: percutaneous coronary intervention (PCI); coronary artery bypass grafting (CABG); conservatively managed (medication only) acute coronary syndrome (ACS). Patients ≥18 years old will be eligible if, in CPRD records, they have: ≥1 year of data before the index event; no prescription for DAPT or anticoagulants in the preceding 3 months; a prescription for aspirin or DAPT within 2 months after discharge from the index event. The primary outcome will be any bleeding event (CPRD or HES) up to 12 months after the index event. We will estimate adjusted HR for time to first bleeding event comparing: aspirin and clopidogrel (reference) versus aspirin and prasugrel or aspirin and ticagrelor after PCI; and aspirin (reference) versus aspirin and clopidogrel after CABG and ACS. We will describe rates of bleeding in patients prescribed TT (DAPT plus an anticoagulant). Potential confounders will be identified systematically using literature review, semistructured interviews with clinicians and a short survey of clinicians. We will conduct sensitivity analyses addressing the robustness of results to the study’s main limitation—that we will not be able to identify the intervention group for patients whose bleeding event occurs before a DAPT prescription in CPRD.

**Ethics and dissemination:**

This protocol was approved by the Independent Scientific Advisory Committee for the UK Medicines and Healthcare Products Regulatory Agency Database Research (protocol 16_126R) and the South West Cornwall and Plymouth Research Ethics Committee (17/SW/0092). The findings will be presented in peer-reviewed journals, lay summaries and briefing papers to commissioners/other stakeholders.

**Trial registration number:**

76607611; Pre-results.

**Strengths and limitations of this study**

- We designed our study using the framework recommended by the Cochrane Bias and Non-Randomised Studies Methods Groups for establishing appropriate patient populations, interventions and follow-up to emulate three hypothetical randomised controlled trials (target trials).
- We will identify potential confounders systematically using literature review, semistructured interviews with clinicians (cardiologists, cardiac surgeons and general practitioners) and a short survey with an additional group of clinicians.
- Because there are no medication data in Hospital Episode Statistics, we will assume that patients’ first dual antiplatelet therapy (DAPT) prescription that appears in Clinical Practice Research Datalink (CPRD) after their hospital admission is what they were prescribed at discharge.
- We will conduct sensitivity analyses to address the robustness of results to different assumptions about the unknown intervention group in patients who died or had a bleeding event before a DAPT prescription in CPRD.

## Introduction

Dual antiplatelet therapy (DAPT), a combination of aspirin and either clopidogrel, prasugrel or ticagrelor, is recommended for secondary prevention of ischaemic events (heart attack and stroke) in people with coronary artery disease. Guidelines recommend that patients are treated with DAPT for 6 to 12 months following myocardial infarction (MI) and coronary interventions (percutaneous coronary intervention [PCI] and coronary artery bypass grafting [CABG])^1–3^ and support the use of the more potent antiplatelet inhibitors ticagrelor and prasugrel.^2^ Antiplatelet agents reduce the risk of ischaemic events, by preventing the formation of clots in atherosclerotic coronary arteries and within stents (following PCI) or grafts (following CABG), but increase the risk of bleeding.^4^ Randomised controlled trials (RCTs) have shown that adding clopidogrel to aspirin leads to 1% excess risk of major bleeding (requiring admission to hospital) compared with aspirin alone.^5 6^ Prasugrel and ticagrelor reduce the risk of ischaemic events further but also further increase the risk of bleeding.^7^ Some patients (eg, those with existing atrial fibrillation or those who develop atrial fibrillation after PCI, CABG or acute coronary syndrome [ACS]) are prescribed an anticoagulant (eg, warfarin, dabigatran, rivaroxaban, apixaban) in addition to DAPT (triple therapy [TT]), which further increases the risk of bleeding.

‘Real world’ bleeding events that do not require any intervention are likely to be much more frequent than those reported in RCTs, which excluded patients at high risk of bleeding and mainly reported only on major bleeding. Bleeding events that do not result in hospitalisation are largely managed in primary care and may have a significant clinical and economic impact.^8^ Minor and nuisance bleeding (nose and gum bleeds, bruising and prolonged bleeding from cuts) may also reduce adherence to DAPT^9^ and non-adherent patients may be at increased risk of a secondary ischaemic episode.^10^ Only three studies have reported the incidence and consequences of nuisance bleeding after DAPT^11–13^; these suggest that nuisance bleeding is common (affecting 29%–38% of patients) and impacts on adherence (11% of patients in one study discontinued clopidogrel^12^). A nested case–control study using the Health Improvement Network (a UK primary care database) reported an increased risk of upper gastrointestinal bleeding with clopidogrel and aspirin compared with aspirin alone (relative risk, 2.08; 95% CI 1.34 to 3.21).^14^

The economic impact of bleeding events associated with DAPT is also poorly characterised, in particular for minor bleeding events and their impact on health-related quality of life.^8^ This is not surprising given that health economic analyses often lack detailed data on adverse effects of interventions, despite consensus that such effects should be considered.^15 16^ To ensure appropriate decisions are made about which DAPT regimens to use in clinical practice, the health and resource use consequences of minor and major bleeding events should be incorporated into assessments of cost-effectiveness. For DAPT, this entails accounting for uncertainty in the absolute risk of bleeding, the impact of different bleeding events on health-related quality of life and treatment adherence and subsequent risk of secondary ischaemic events and the cost implications of managing these bleeding events.

We propose to use Hospital Episode Statistics (HES) and the Clinical Practice Research Datalink (CPRD) databases to estimate the incidence of all bleeding events occurring in patients prescribed different DAPT or TT regimens after undergoing coronary interventions (PCI and CABG) and in conservatively managed patients with ACS. Our study will also provide parameter estimates to update existing cost-effectiveness models. We will use the framework recommended by the Cochrane Bias and Non-Randomised Studies Methods Groups for establishing appropriate patient populations, interventions and follow-up to emulate the following three hypothetical RCTs (hereafter referred to as the target trials, table 1)^17^.

**Table 1:** Summary of three target trials and how observational data will be used to emulate these

PICO component	Target trial	Issues in emulating the target trial using observational data
Eligibility criteria	Target trial 1 (PCI) Consecutive patients (age ≥18 years) undergoing PCI (emergency or elective). Exclusions: DAPT or anticoagulant use in the previous 3 months; major bleed requiring hospitalisation in previous 12 months; renal failure requiring dialysis; intolerance/allergy to aspirin, clopidogrel, prasugrel or ticagrelor. Target trial 2 (CABG) Consecutive patients (age ≥18 years) undergoing CABG (urgent and elective). Exclusions: DAPT or anticoagulant use in the previous 3 months; other concomitant cardiac surgery (eg, valve surgery); major bleed requiring hospitalisation in previous 12 months; renal failure requiring dialysis; intolerance/allergy to aspirin, clopidogrel, prasugrel or ticagrelor. Target trial 3 (conservatively managed ACS) Consecutive patients (age ≥18 years) hospitalised for an acute coronary syndrome (ACS): myocardial infarction (MI) with or without ST elevation or unstable angina. Exclusions: PCI or CABG performed at time of ACS diagnosis; major bleed requiring hospitalisation in previous 12 months; renal failure requiring dialysis; intolerance/allergy to aspirin, clopidogrel, prasugrel or ticagrelor.	CPRD-HES linked data set contains information that allows us to identify all eligible patients for the three target trials. The study period is April 2009–July 2017. All eligible patients will have sufficient data (1 year) preceding their index event to apply the exclusion criteria and characterise the population (eg, comorbidities) and sufficient follow-up data (1 year) to identify outcomes. It is not possible to capture intolerance/allergy to aspirin, clopidogrel, prasugrel or ticagrelor.
Interventions	Target trial 1 (PCI) Clopidogrel (75 mg daily) or prasugrel (5 mg or 10 mg daily) or ticagrelor (90 mg twice daily). All patients will receive aspirin (at a dose of 75 mg daily, in line with current guidelines). Target trial 2 (CABG) Clopidogrel (75 mg) in addition to aspirin (at a dose of 75 mg daily, in line with current guidelines) or aspirin only (any dose, reflecting variation in usual care). Target trial 3 (conservatively managed ACS) As for target trial 2.	Relevant interventions can be identified as CPRD has information on all medications (including doses) prescribed in primary care.
Assignment to interventions	Participants are assigned to DAPT interventions in hospital.	Participants enter the study at index procedure date for PCI and CABG, and episode start date for ACS, and will be assigned to DAPT interventions using first prescription in CPRD (within 2 months of hospitalisation) as a proxy for what they were prescribed in hospital (there are no medications data in HES). This assignment will exclude a proportion of eligible patients (those who died or experienced a major bleed that caused them to stop DAPT, or patients who have no prescription for DAPT within the 2-month window); we will identify and describe the characteristics of these excluded patients. In sensitivity analyses, we will address the robustness of results to different assumptions about the intervention group in those patients where the DAPT medication is unknown or a major bleed occurs prior to the first DAPT medication, by using multiple imputation models for handling missing data. Prior known information regarding the likely prescription based on patient characteristics or general policies will be incorporated in these analyses.
Follow-up	Starts at assignment to intervention and ends at first bleed or 12 months from assignment (whichever comes first).	Starts at time of hospitalisation for PCI, CABG or ACS and ends at first bleed or 12 months from hospitalisation (whichever comes first).
Primary outcome	Any bleed within 12 months of the start of DAPT (DAPT is prescribed at hospitalisation for PCI, CABG or ACS).	Any bleed within 12 months of hospitalisation for PCI, CABG or ACS.
Analysis	Intention to treat	According to first prescription for DAPT in CPRD

CABG, coronary artery bypass grafting; CRPD, Clinical Practice Research Datalink; DAPT, dual antiplatelet therapy; HES, Hospital Episode Statistics; PCI, percutaneous coronary intervention; PICO, Population, intervention, comparator, outcome.

1. In patients who have undergone PCI, estimate the effect on bleeding events of assignment to aspirin and clopidogrel (reference) versus aspirin and prasugrel or aspirin and ticagrelor.
2. In patients who have undergone CABG, estimate the effect on bleeding events of assignment to aspirin (reference) versus aspirin and clopidogrel.
3. In patients who are conservatively managed after presenting with ACS, estimate the effect on bleeding events of assignment to aspirin (reference) versus aspirin and clopidogrel.

### Methods

#### Data sources

CPRD is a database of primary care electronic health record data (available online via CPRD GOLD) from participating general practices, covering 7% of the UK population.^18^ Patients included in CPRD are largely representative of the UK population in terms of age, sex, ethnicity and body mass index. HES covers all hospital admissions for all English patients whose treatment is funded by the UK National Health Service (NHS), whether treated by the NHS or by independent providers.^19^ Seventy-five per cent of English general practices included in CPRD are linked to HES data.^18^ We obtained data from 1 April 2009 to 31 July 2017; this period covers the introduction of the newer antiplatelet agents prasugrel and ticagrelor. This study protocol has been approved.

### Study populations

**Figure 1:**
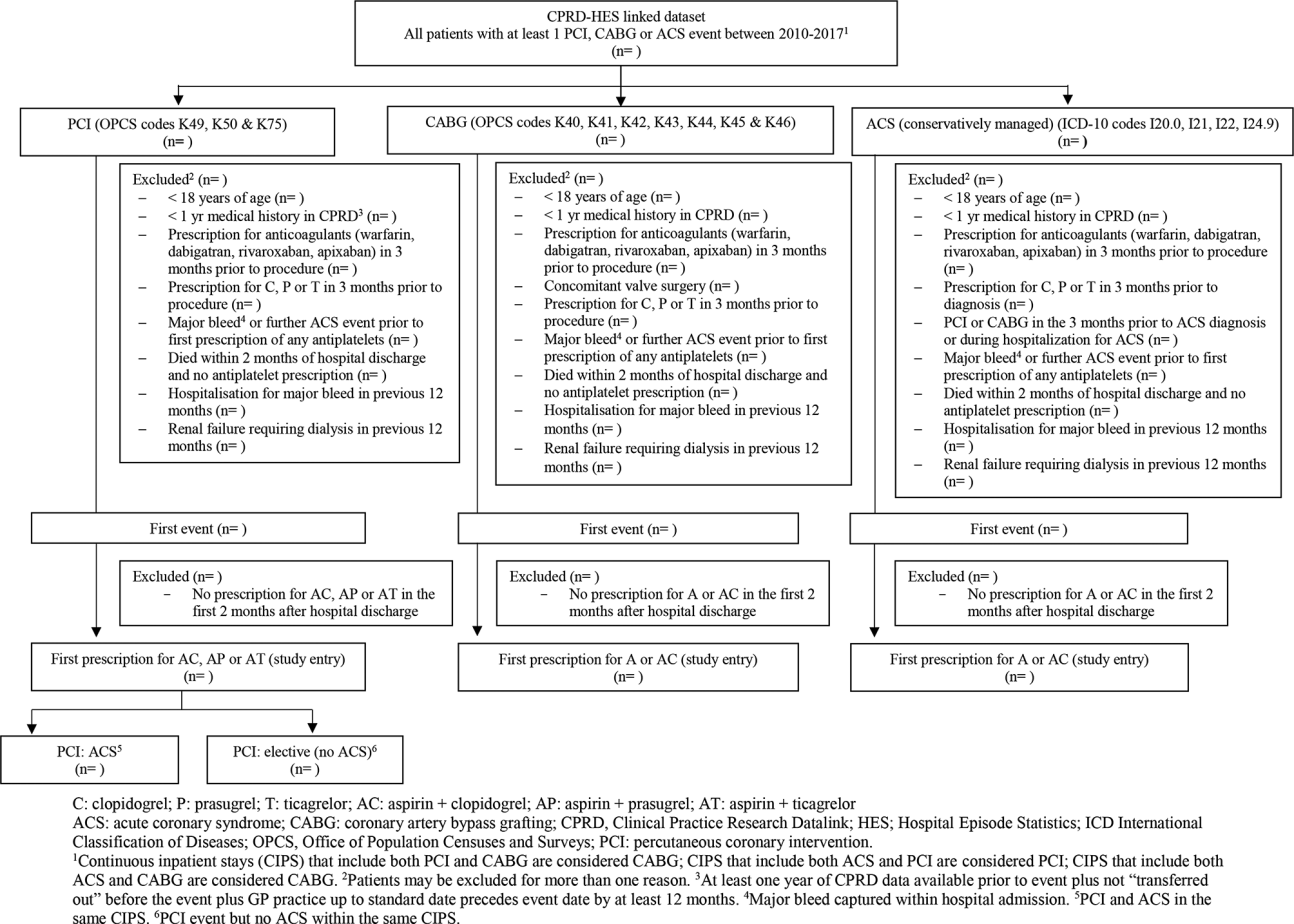
Study diagram describing the construction of the PCI, CABG and ACS (conservatively managed) populations.

Eligibility and exclusion criteria for the three target trials (for patients undergoing PCI, patients undergoing CABG or patients hospitalised and conservatively managed for ACS) are listed in table 1. We will identify eligible patients who are included in Clinical Practice Research Datalink (CPRD) and eligible for linkage with Hospital Episode Statistics (HES) and Office for National Statistics (ONS) mortality data (because they have a valid NHS number and are registered at a practice that was participating in the linkage programme). Patients are included if they had a PCI, CABG or ACS (index event) record in HES during the study period (1 April 2010–31 January 2017), and have at least 1 year of linked CPRD-HES data before the date of their index event. They must also have been prescribed one of the treatment regimens being compared in the target trial corresponding to their index event. One year’s data preceding eligibility for the target trial is adequate to apply most of the exclusion criteria and determine comorbidities and medication history: such information would be collected at baseline in a randomised trial. The following Office of Population Censuses and Surveys (OPCS) procedure codes (PCI and CABG) and International Classification of Diseases (ICD-10) codes (ACS no procedure) will be used to identify patients: PCI, K49, K50 & K75; CABG, K40, K41, K42, K43, K44, K45 & K46; ACS without a procedure, I20.0, I21, I22, I24.9 (with no OPCS code for PCI or CABG in the same hospital admission). Figure 1 shows full details of the inclusion and exclusion criteria.

### Interventions

The interventions of interest for the three target trials are shown in table 1. Guidelines recommend low dose aspirin (75 to 100 mg/d) plus either clopidogrel (75 mg/d), prasugrel (5 mg/d or 10 mg/d) or ticagrelor (90 mg twice/d) for PCI and conservatively managed patients with ACS. For PCI patients, the interventions of interest are aspirin and clopidogrel, aspirin and prasugrel and aspirin and ticagrelor. In conservatively managed patients with ACS, clopidogrel is the most commonly prescribed second antiplatelet agent (in addition to aspirin) and a large proportion of patients are prescribed aspirin only; therefore, the interventions of interest are aspirin only (75 to 100 mg/day) and aspirin and clopidogrel. There is variation in aspirin prescription for CABG patients; some surgeons choose 75 mg/day, others 150 mg/day or 300 mg/day. Surgeons may also prescribe an additional antiplatelet agent, most commonly clopidogrel. Therefore, the comparisons of interest in CABG patients are aspirin only (any dose, reflecting variations in usual care in different hospitals) and aspirin and clopidogrel (doses as for PCI). We have specified these comparisons based on preliminary feasibility counts from CPRD, which indicate that few CABG and conservatively managed patients with ACS are prescribed aspirin and prasugrel or aspirin and ticagrelor.

In the target trials, the interventions would be assigned during the hospital stay, as soon as patients are eligible for antiplatelet therapy. Our observational data set does not have information on medication given to patients at discharge, because HES does not include medications data. Therefore, the first time at which we have information on the antiplatelet regimen to which patients were assigned in hospital is when they receive their first primary care prescription/s for aspirin or DAPT, recorded in CPRD. It is reasonable to use these as a proxy for the medications that patients started in hospital, because patients’ general practitioners (GPs) are unlikely to change the prescriptions that were started in hospital.

We will classify patients according to the first prescription recorded in CPRD during the first 2 months after hospitalisation for PCI, CABG or ACS. This 2-month window is based on variability in the amount of DAPT medication provided to patients in hospital following their PCI, CABG or ACS and hence variability in the time when they first request a repeat prescription from their general practice. A preliminary investigation showed that more than 75% of eligible patients have a prescription for one or more antiplatelet agents during this time period. If a patient only has a prescription for aspirin during the 2-month window after hospital discharge, they will be assigned to an aspirin only intervention. If patients also receive a prescription for clopidogrel, prasugrel or ticagrelor, they will be assigned to aspirin/clopidogrel, aspirin/prasugrel or aspirin/ticagrelor. If there is a prescription for more than one additional antiplatelet agent in the 2-month window, we will assign the patient to an intervention based on the agent prescribed first. For example, if a patient has an aspirin prescription and a prescription for clopidogrel before a prescription for ticagrelor, the patient will be assigned to the aspirin/clopidogrel intervention. Patients with no prescriptions in CPRD for aspirin, or aspirin and clopidogrel, prasugrel or ticagrelor within the 2-month window will be excluded from the main analysis. We will conduct sensitivity analyses of all eligible patients including those with unknown DAPT regimens or a major bleed prior to first DAPT prescription, by estimating assignment to DAPT interventions for those with no prescription data using multiple imputation based on a range of assumptions.^20^

### Outcomes

The primary outcome will be any bleeding event, classified as type 2–5 by the Bleeding Academic Research Consortium (BARC) bleeding scale.^21^ For each patient, we will identify all bleeding events in HES and CPRD during follow-up. We will not be able to identify BARC type 1 bleeding events (bleeding that is not actionable and does not cause the patient to seek treatment) as type 1 assumes there is no interaction with the health system or healthcare professionals; therefore, no bleed event will be recorded in HES or CPRD. We have specified a comprehensive list of bleeding codes in CPRD and HES (see online supplementary appendix 1). These will be categorised according to anatomical site for descriptive purposes. Secondary outcomes will be: any major bleeding event (leading to hospital admission, inpatient HES); any minor bleeding event (recorded within CPRD data); all-cause mortality; cardiovascular mortality; mortality from bleeding (these will be identified from linked ONS data); MI; stroke; additional coronary intervention.

10.1136/bmjopen-2019-029388.supp1 Supplementary file 1

### Follow-up

The start of follow-up (the index event) will be the date of the index hospital procedure (PCI, CABG) or start date of the hospital episode that contains the ACS diagnosis (ACS). Patients will be followed up until 12 months after the index event, since DAPT is prescribed for 12 months in accordance to guidelines.

### Confounding and cointerventions

Potential confounders (variables that predict both risk of bleeding and intervention group) will be specified a priori.^22 23^ We will identify confounders and cointerventions using literature review and clinician expertise as recommended by the Cochrane Bias and Non-Randomised Studies Methods Groups.^17^ We will carry out a comprehensive and systematic literature search to identify all RCT and cohort studies of DAPT interventions, or cohort studies that identify predictors of bleeding. The literature searches for the review are included in supplementary appendix 2. Abstracts will be screened by one researcher and full-text papers will be obtained. Data on confounders and cointerventions will be extracted by two researchers independently using a data extraction form specifically designed for the study; variables extracted will include study characteristics, population characteristics (reported in the tables of baseline characteristics), factors adjusted for in the statistical analyses and factors reported to predict risk of bleeding in our populations. We will not perform a risk of bias assessment because the aim of the review is only descriptive (ie, the output will be lists of confounders and cointerventions) and there are no established criteria for assessing the validity with which primary researchers consider potential confounders and cointerventions; therefore, it would be inappropriate to apply a risk-of-bias tool for studies estimating a treatment effect. We will use ‘saturation’ as a criterion for discontinuing data collection, defined as review of 10 consecutive studies without identifying an additional confounder/cointervention.

10.1136/bmjopen-2019-029388.supp2 Supplementary file 2

In parallel, we will conduct semiquantitative interviews with six clinicians in each of three groups: cardiologists; cardiac surgeons and GPs (to determine whether DAPT prescriptions are changed in primary care). The main aim of the clinician interviews is to understand DAPT prescribing practice in the UK and identify the factors (relating to patients, centres and prescribing practices of the individual doctors) that influence the decision about which antiplatelet regimen to prescribe. All factors that influence DAPT prescribing (confounders) identified from the literature review and clinician interviews will be combined in a short survey (SurveyMonkey). The survey questionnaire will be administered online to all consultant members of the British Cardiovascular Society (cardiologists) and the Society for Cardiothoracic Surgery (cardiac surgeons). The survey will be either emailed to all members individually (if the professional bodies agree) or advertised in weekly/monthly newsletters. Confounders will be grouped in confounding domains.^17^ We will attempt to identify each potential confounder (identified through the literature review, clinician interviews and survey) in the CPRD or HES data set but acknowledge that there may be missing data across patients (and time) for some confounders.

### Sample size

Estimated rates of bleeding with the different therapies are 5% for aspirin, 9% for aspirin/clopidogrel and 12% for aspirin/prasugrel and aspirin/ticagrelor.^5 6 24 25^ Preliminary feasibility counts provided by CPRD suggest that there will at least the following numbers of patients eligible for each target trial:

- PCI: aspirin/clopidogrel (reference, 6738 patients) versus aspirin/prasugrel (842 patients) or aspirin/ticagrelor (770 patients)
- CABG: aspirin (reference, 2556 patients) versus aspirin/clopidogrel (595 patients)
- Conservatively managed ACS: aspirin (reference, 8148 patients) versus aspirin/clopidogrel (3082 patients)

These estimates give expected event rates of at least 700 for PCI, 180 for CABG and 680 for ACS, assuming a ratio of 8:1 (aspirin/clopidogrel:aspirin/prasugrel or aspirin/clopidogrel:aspirin/ticagrelor) for PCI, 4:1 (aspirin:aspirin/clopidogrel) for CABG and 2.5:1 (aspirin:aspirin/clopidogrel) for ACS. The HR detectable with 90% and 80% power at the 5% statistical significance, assuming the group ratios given above, are shown in table 2. The correlation of the DAPT with other covariates adjusted for is unknown and we assessed the impact of a range of correlations (0, 0.3 and 0.5).

**Table 2:** HR for a range of correlations for percutaneous coronary intervention (PCI), coronary artery bypass grafting (CABG) and acute coronary syndrome (ACS)

Ratio of presence: absence of covariate	Squared correlation with other covariates	HR detectable	
		90% power	80% power
PCI			
8:1	0 (ie, unadjusted)	1.48	1.41
	0.3	1.60	1.50
	0.5	1.74	1.62
CABG			
4:1	0 (ie, unadjusted)	1.83	1.69
	0.3	2.06	1.87
	0.5	2.35	2.10
Conservatively managed ACS			
2.5:1	0 (ie, unadjusted)	1.32	1.27
	0.3	1.39	1.33
	0.5	1.48	1.40

### Statistical analyses

We will describe temporal changes in DAPT prescribing and bleeding for PCI, CABG and ACS populations. We will use descriptive statistics to summarise the characteristics of the different intervention groups and standardised mean differences to compare them. We will estimate rates of bleeding (number of events/person time) with 95% CIs for each group. We will separate major and minor bleeding since adverse events of each type have different health and resource use consequences.

Analyses will estimate the effects of assigned intervention (analogous to an intention-to-treat analysis of a randomised trial) for the antiplatelet regimens corresponding to the first prescription of aspirin or DAPT in CPRD (see Interventions). We will use parametric survival models to estimate adjusted HRs with 95% CIs for the time to first bleeding event, comparing intervention groups for each target trial. Exploratory analyses, including assessment of proportional hazards assumptions, will be used to inform the choice of survival distribution (eg, Weibull). Estimated time-dependent event probabilities will be used to update existing cost-effectiveness models.^26^ The confounding factors to be included in the model (which will be identified as described previously and grouped into confounding domains from our data set), the modelling strategy and the approach to handling correlated covariates will be documented in a data analysis plan. Participants free from a bleeding event will be censored at 12 months after the index event. For secondary endpoints, we will use survival models to estimate adjusted HRs with 95% CIs for time to first event. For mortality outcomes, we will take account of the competing risks of death due to other causes.

We will perform three sets of sensitivity analyses:

1. We will address the unknown intervention group of eligible patients who have no prescription data and therefore cannot be assigned to an intervention (ie, those that died before receiving their first prescription, had a major bleed which caused them to stop DAPT or have no aspirin/DAPT prescription recorded in CPRD within the 2-month window). These analyses will be undertaken using multiple imputation methods to deal with missing information on DAPT medication and will take patient characteristics, procedure/diagnosis and general medication policies into account, using a range of assumptions.
2. We will address the possibility that some minor bleeding events are not documented in CPRD but nevertheless prompt medication changes. We anticipate that most bleeding events will occur soon after the index event and before any medication change. Medication changes will be described relative to bleeding events observed (eg, before event, after event, no bleeding event observed). If a substantial proportion (>10%) of people change medication before their first bleeding event, we will perform a sensitivity analysis excluding these patients.
3. We will assess the sensitivity of our findings when the analysis is restricted to a subpopulation of patients at low risk of bleeding (ie, excluding people at high risk for bleeding) who, we hypothesise, have the lowest risk of residual confounding.

We will attempt to identify an instrumental variable (eg, clinician/GP practice prescribing practice) to control for confounding by indication. If we are successful, we will repeat the above analyses for the PCI, CABG and ACS populations.

We will explore the consistency of treatment effect estimates in the following subgroups: ACS versus non-ACS (PCI and CABG populations); diabetic versus non-diabetic; chronic kidney disease versus non-chronic kidney disease; concurrent prescription for proton pump inhibitors versus no prescription for proton pump inhibitors. All subgroups will be defined in the data analysis plan and chosen based on the characteristics of the analysis populations before carrying out any analyses by intervention group. We will report p values from tests of interaction.

### Triple therapy

**Figure 2:**
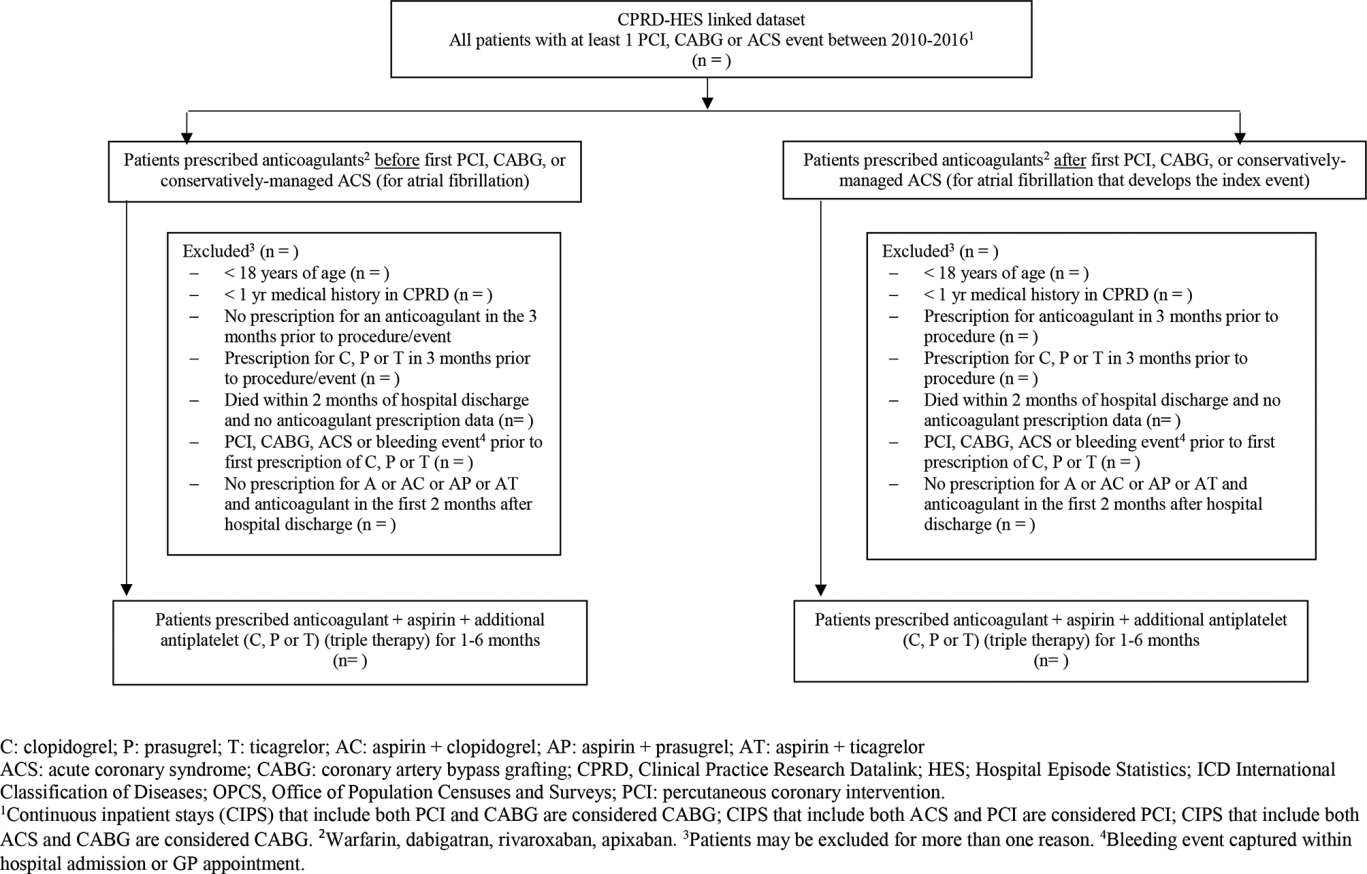
Study diagram describing the construction of the triple therapy (TT) populations.

We will establish additional groups of patients (PCI or CABG or conservatively managed ACS) based on TT prescription. The main reason for prescribing an anticoagulant is to treat atrial fibrillation in a patient otherwise eligible for either of the three target trials. Atrial fibrillation can precede (long-term anticoagulation for pre-existing atrial fibrillation) or follow the index event (anticoagulation for new-onset atrial fibrillation). Figure 2 shows how the patient groups receiving TT will be constructed.

We will identify patients on long-term anticoagulation by identifying prescriptions of oral anticoagulants (warfarin, dabigatran, rivaroxaban, apixaban) in the 3 months before their index event and a concomitant prescription of any anticoagulant (TT) with DAPT and in the first 2 months after the index admission. Patients who develop atrial fibrillation after the index event will be identified from any new anticoagulant prescription during follow-up. We have compiled a list of all drug codes in CPRD for antiplatelet agents (aspirin, clopidogrel, prasugrel and ticagrelor) and anticoagulants (warfarin, dabigatran, rivaroxaban, apixaban) (see supplementary appendix 3).

10.1136/bmjopen-2019-029388.supp3 Supplementary file 3

In patients receiving various TT regimens (grouped by pre-index event anticoagulant prescription and post-index event anticoagulant prescription in PCI, CABG and ACS separately, see figure 2), we will describe rates of bleeding (number of events/person time) with 95% CIs for each group. We have not specified comparison groups because we know there is large variation in prescribing of anticoagulants; TT is usually prescribed for a relatively short period of time (1–6 months, depending on a patient’s individual risks of thrombosis and bleeding) after which an antiplatelet agent (usually aspirin) is removed and patients continue to receive an anticoagulant and single antiplatelet for the remainder of the 12 months after the index admission.

### Patient and public involvement

We set up a patient advisory group comprising of patients from our three cohorts (PCI, CABG and conservatively managed ACS). The group was consulted about several aspects of the proposed study. Group members confirmed the importance of the research topic and agreed that the choice of antiplatelet regimen should be based on shared decision making, with clinicians and patients weighing the potential benefits against the adverse side effects and practical inconvenience. Our PPI group will attend regular meetings to discuss results from the study and will also contribute and collaborate on the development of our results dissemination strategy.

### Ethics and dissemination

This study protocol has approval. We also obtained ethical approval for the semistructured interviews with clinicians and survey from South West Cornwall and Plymouth Research Ethics Committee, 17/SW/0092. The findings will be presented at national/international conferences, published in peer-reviewed academic journals and accessible formats in newsletters to patients (where available). The findings will also be reported as a briefing paper to commissioners (eg, commissioning groups, National Institute for Health and Care Excellence) and to other healthcare stakeholders with an interest in the research through the Cardiac & Stroke Networks. We will also present and discuss our results at local CPRD working groups to disseminate methods to other researchers using the CPRD database.

## Supplementary Material

Reviewer comments

Author's manuscript
