# Supplementary material for: Comprehensive ascertainment of bleeding in patients prescribed different combinations of dual antiplatelet therapy (DAPT) and triple therapy (TT) in the UK: study protocol for three population-based cohort studies emulating ‘target trials’ (the ADAPTT Study)
Source: BMJ Open. 2019 Jun 4;9(6):e029388. doi: 10.1136/bmjopen-2019-029388 (PMC6561407; doi:10.1136/bmjopen-2019-029388)
Supplement: Supplementary file 1 [file bmjopen-2019-029388supp001.pdf]

Appendix 1: List of Read codes and ICD-10 diagnosis codes for bleeding in Clinical Practice Research Datalink (CPRD) and Hospital Episode Statistics (HES)

Read Code	Read Term
J573011	Rectal bleeding
R047.00	[D]Epistaxis
158..12	Vaginal bleeding
K5A1.00	Postmenopausal bleeding
K59z.11	Break - through bleeding
1C6..11	Epistaxis symptom
J573.11	Bleeding PR
F4C7100	Subconjunctival haemorrhage
K597.00	Postcoital bleeding
16B3.00	Spontaneous bruising
1C6..00	Nose bleed symptom
K59y300	Intermenstrual bleeding
SE4..11	Leg bruise
K596.11	Intermenstrual bleeding - irregular
K59yx11	Dysfunctional uterine bleeding
196C.00	Painless rectal bleeding
SE4z.11	Haematoma NOS
J573000	Rectal haemorrhage
SE...11	Haematoma with intact skin
..00	Bruising symptom
SE3..11	Arm bruise
22E9.00	O/E-subconjunctival haemorrh'g
K59yx00	Dysfunctional uterine haemorrhage NOS
G60..00	Subarachnoid haemorrhage
F4C7200	Conjunctival haemorrhage NOS
G848000	Bleeding haemorrhoids NOS
R047.11	[D]Nosebleed
SE33011	Subungal haematoma
R027.11	[D]Spontaneous bruising
K596.00	Metrorrhagia
2I15.00	O/E - bruising
J68z.11	GIB - Gastrointestinal bleeding
196B.00	Painful rectal bleeding
J68..00	Gastrointestinal haemorrhage
SE23111	Perianal haematoma
1C62.00	Has nose bleeds - epistaxis
G8y0.00	Haemorrhage NOS
K5E..00	Other abnormal uterine and vaginal bleeding
SE10.00	Black eye NOS
K56y111	Bleeding - vaginal NOS

G61..00	Intracerebral haemorrhage
F42y500	Retinal haemorrhage NOS
F4K2800	Vitreous haemorrhage
J573.00	Haemorrhage of rectum and anus
G844.11	Perianal haematoma
2D85.00	O/E - blood in auditory canal
SE46.00	Traumatic haematoma
2BB8.00	O/E - vitreous haemorrhages
J68zz00	Gastrointestinal tract haemorrhage NOS
1C6Z.00	Nose bleed symptom NOS
SP21300	Primary post tonsillectomy haemorrhage
S622.00	Closed traumatic subdural haemorrhage
K575.00	Haematoma of vulva
K19y411	Urethral bleeding
K31y000	Breast haematoma due to nontraumatic cause
S62..13	Subdural haemorrhage following injury
7032000	Evacuation of extradural haematoma
SP21400	Secondary post tonsillectomy haemorrhage
K19y400	Bleeding from urethra
J68z000	Gastric haemorrhage NOS
J150000	Acute haemorrhagic gastritis
J510900	Bleeding diverticulosis
R09z000	[D]Umbilical bleeding
J68z100	Intestinal haemorrhage NOS
G60z.00	Subarachnoid haemorrhage NOS
K5E1.00	Abnormal uterine bleeding, unspecified
S62A.00	Traumatic extradural haematoma
J573100	Anal haemorrhage
F404500	Intra-ocular haemorrhage
SP21.12	Haemorrhage - postoperative
J120100	Acute duodenal ulcer with haemorrhage
S62..00	Cerebral haemorrhage following injury
SE22300	Haematoma of rectus sheath
J121111	Bleeding chronic duodenal ulcer
G62z.00	Intracranial haemorrhage NOS
G617.00	Intracerebral haemorrhage, intraventricular
J110111	Bleeding acute gastric ulcer
J68z.00	Gastrointestinal haemorrhage unspecified
K5C2.00	Haematocolpos
F503100	Haematoma of pinna
K286100	Scrotal haemorrhage
SP03216	Bleeding due to intrauterine contraceptive device
SE42011	Heel bruise
7M0G000	Aspiration of haematoma of organ NOC

K286v00	Male genital haematoma NOS
K566.00	Vaginal haematoma
SP21200	Post-operative haematoma formation
G602.00	Subarachnoid haemorrhage from middle cerebral artery
S62..11	Extradural haemorrhage following injury
F42y400	Subretinal haemorrhage
7004300	Evacuation of intracerebral haematoma NEC
G77z000	Capillary haemorrhage
SE06.00	Bruise of mandibular joint area
K544.00	Haematometra
S760100	Kidney haematoma without mention of open wound into cavity
K138300	Intrarenal haematoma
K59yy00	Functional uterine haemorrhage NOS
S760111	Renal haematoma without mention of open wound into cavity
7M0U400	Reexploration of organ & surgical arrest postop bleeding NOC
J111100	Chronic gastric ulcer with haemorrhage
7004200	Evacuation of haematoma from cerebellum
SP03217	Contraception IUCD causing bleeding
K275100	Corpus cavernosum haematoma
K286400	Testicular haemorrhage
ZA13700	Drainage of subungual haematoma with hot wire
S628.00	Traumatic subdural haemorrhage
7008200	Aspiration of haematoma of brain tissue
SE11.12	Bruise of periocular tissue
7H02200	Reopen chest reexplore intraabdom op site surg arr PO bleed
S624.00	Closed traumatic extradural haemorrhage
F4G3200	Exophthalmos due to orbital haemorrhage
S740100	Liver haematoma and contusion without open wound into cavity
S620.00	Closed traumatic subarachnoid haemorrhage
TA0..11	Accidental haemorrhage during medical care
K221100	Prostatic haemorrhage
SP21000	Intra-operative haemorrhage
S626.00	Epidural haemorrhage
7404z00	Surgical arrest of bleeding from internal nose NOS
C063000	Thyroid haemorrhage
ZA13800	Drainage of subungual haematoma with drill
Kyu9D00	[X]Other specified abnormal uterine and vaginal bleeding
H5y0000	Tracheostomy haemorrhage
7E0F500	Uterus operation haemostasis
7H22600	Reopen abdo reexplore intraabd op site surg arr postop bleed
F436z00	Choroidal haemorrhage or rupture NOS
Gyu6200	[X]Other intracerebral haemorrhage
F501G00	Haemorrhagic otitis externa
D305000	Haemorrhagic disorder due to antithrombinaemia

J11yy00	Unspec gastric ulcer; unspec haemorrhage and/or perforation
J13y100	Unspecified peptic ulcer with haemorrhage
D305.00	Haemorrhagic disorder due to circulating anticoagulants
SK02.11	Secondary and recurrent haemorrhage
7404y00	Surgical arrest of bleeding from internal nose OS
J111300	Chronic gastric ulcer with haemorrhage and perforation
K138100	Renal artery haemorrhage
C154200	Adrenal haemorrhage
F42y300	Deep retinal haemorrhage
J12yy00	Unspec duodenal ulcer; unspec haemorrhage and/or perforation
J130300	Acute peptic ulcer with haemorrhage and perforation
K167.00	Haemorrhage into bladder wall
7609y11	Tanner devascularisation for bleeding varices
2DE7.00	O/E - throat haemorrhage
J14y100	Unspecified gastrojejunal ulcer with haemorrhage
Gyu6100	[X]Other subarachnoid haemorrhage
F437200	Haemorrhagic choroidal detachment
7303200	Drainage haematoma ext ear control cavity c bolster suture
Ryu7300	[X]Haemorrhage, not elsewhere classified
J13y300	Unspecified peptic ulcer with haemorrhage and perforation
Gyu6F00	[X]Intracerebral haemorrhage in hemisphere, unspecified
G852000	Oesophageal varices with bleeding in diseases EC
SE45.11	Haematoma of leg
1584	Heavy episode of vaginal bleeding
7017000	Evacuation of subdural haematoma
SP21.11	Haematoma - postoperative
J573012	PRB - Rectal bleeding
SE30011	Shoulder bruise
2D25.00	O/E - epistaxis
SE1..11	Bruise of eye
F42y.11	Haemorrhage - retinal
K5Ez.00	Abnormal uterine and vaginal bleeding, unspecified
7736000	Evacuation of perianal haematoma Subdural haemorrhage - nontraumatic
G621.00 K286000	Scrotal haematoma due to nontraumatic cause
K56y100	Haemorrhage of vagina
K56y112	BPV - Vaginal bleeding
SP21.00	Peri-operative haemorrhage or haematoma
K286w00	Male genital haemorrhage NOS
G61z.00	Intracerebral haemorrhage NOS
SE0..11	Bruise of face
16B2.00	Bruises easily
G622.00	Subdural haematoma - nontraumatic
SP21100	Post-operative haemorrhage

K59y.11	Metropathia haemorrhagica
S629.00	Traumatic subdural haematoma
2556	O/E - bleeding gums
J68z200	Upper gastrointestinal haemorrhage
F586200	Otorrhagia
G61..12	Stroke due to intracerebral haemorrhage
G842000	Internal bleeding haemorrhoids
G530.00	Haemopericardium
SK02.12	Secondary and recurrent haemorrhage
K587.00	Contact bleeding of cervix
G850.00	Oesophageal varices with bleeding
G61..11	CVA - cerebrovascular acid due to intracerebral haemorrhage
1928	Bleeding gums
2BB5.00	O/E - retinal haemorrhages
K5E2.00	Abnormal vaginal bleeding, unspecified
SE0..12	Bruise of head
7M0G400	Evacuation of haematoma NEC
SE43.11	Toenail bruise
SE2..11	Bruise, trunk
16BZ.00	Bruising symptom NOS
K599.00	Mid-cycle bleeding
16B4.00	Post-traumatic bruising
G623.00	Subdural haemorrhage NOS
R063000	[D]Cough with haemorrhage
G613.00	Cerebellar haemorrhage
G845000	External bleeding haemorrhoids
J12y100	Unspecified duodenal ulcer with haemorrhage
J573z00	Haemorrhage of rectum and anus NOS
G718.00	Leaking abdominal aortic aneurysm
SE24211	Bruise of scrotum
J110100	Acute gastric ulcer with haemorrhage
2D66.00	O/E - blood from ear
7303000	Drainage of haematoma of external ear
25T0.00	Bleeding stoma
R063100	[D]Pulmonary haemorrhage NOS
F436000	Unspecified choroidal haemorrhage
J10y000	Haemorrhage of oesophagus
G360.00	Haemopericardium/current comp folow acute myocardial infarction
SK02.00	Secondary and recurrent haemorrhage
J121100	Chronic duodenal ulcer with haemorrhage
ZA13600	Drainage of subungual haematoma
K286300	Testicular haematoma due to nontraumatic cause
SE05.11	Bruise of ear
7D05200	Evacuation of haematoma of vulva

J56y000	Haemoperitoneum - nontraumatic
G614.00	Pontine haemorrhage
K5E0.00	Abnormal uterine bleeding unrelated to menstrual cycle
G611.00	Internal capsule haemorrhage
G60X.00	Subarachnoid haemorrhage from intracranial artery, unspecified
K13y800	Perirenal haematoma
7004100	Evacuation of haematoma from temporal lobe of brain
K16y200	Bladder haemorrhage
K221.00	Prostatic congestion or haemorrhage
G61X000	Left sided intracerebral haemorrhage, unspecified
66UI.00	Hormone replacement therapy bleed pattern - abnormal
G620.00	Extradural haemorrhage - nontraumatic
851..00	Haemorrhage control by packing
F4Ey000	Haemorrhage of eyelid
G61X100	Right sided intracerebral haemorrhage, unspecified
7D1C000	Evacuation of haematoma from vagina
SE11.11	Bruise of eyelids
R048. 00	[D]Throat haemorrhage
7404	Surgical arrest of bleeding from internal nose
S750100	Spleen haematoma without mention of open wound into cavity
F436.00	Choroidal haemorrhage and rupture
SE4z.12	Intramuscular haematoma NOS
F4K7.00	Retrobulbar haemorrhage
G603.00	Subarachnoid haemorrhage from anterior communicating artery
S62..12	Subarachnoid haemorrhage following injury
G610.00	Cortical haemorrhage
G62..00	Other and unspecified intracranial haemorrhage
662o.00	Haemorrhagic stroke monitoring
S630.12	Intracranial haematoma following injury
J130100	Acute peptic ulcer with haemorrhage
K55y300	Haemorrhage of cervix
G61X.00	Intracerebral haemorrhage in hemisphere, unspecified
K53y600	Haematosalpinx
J111111	Bleeding chronic gastric ulcer
G605.00	Subarachnoid haemorrhage from basilar artery
2D65.00	O/E - blood stained ear disch.
G612.00	Basal nucleus haemorrhage
F42y100	Superficial retinal haemorrhage
G604.00	Subarachnoid haemorrhage from posterior communicating artery
K59B.00	Postmenopausal postcoital bleeding
761D500	Endoscopic injection haemostasis of duodenal ulcer
S62z.00	Cerebral haemorrhage following injury NOS
K537.00	Haematoma of the broad ligament
7405300	Insertion of Brighton epistaxis balloon

F4H4100	Optic nerve sheath haemorrhage
S624.11	Epidural haematoma following injury
7619100	Gastrotomy and ligation of bleeding point of stomach
K59A.00	Premenopausal postcoital bleeding
S62..14	Traumatic cerebral haemorrhage
S627.00	Traumatic subarachnoid haemorrhage
7G2H400	Liposuction removal of haematoma
J131100	Chronic peptic ulcer with haemorrhage
FyuH400	[X]Vitreous haemorrhage in diseases classified elsewhere
J11y100	Unspecified gastric ulcer with haemorrhage
J120300	Acute duodenal ulcer with haemorrhage and perforation
S629000	Traumatic subdural haematoma without open intracranial wound
G618.00	Intracerebral haemorrhage, multiple localized
F424300	Retinal pigment epithelium haemorrhagic detachment
F436100	Expulsive choroidal haemorrhage
G616.00	External capsule haemorrhage
G615.00	Bulbar haemorrhage
K275200	Corpus cavernosum haemorrhage
761D600	Endoscopic injection haemostasis of gastric ulcer
J017200	Teeth staining due to pulpal bleeding
7L1L300	Haemostasis of unspecified organ
SE05.12	Bruise of auricle
S761100	Kidney haematoma with open wound into cavity
K221z00	Prostatic congestion or haemorrhage NOS
S625.00	Open traumatic extradural haemorrhage
S63..00	Other cerebral haemorrhage following injury
G606.00	Subarachnoid haemorrhage from vertebral artery
7B37400	Open haemostasis of prostate
S63z.00	Other cerebral haemorrhage following injury NOS
7J01300	Reopen cranium reexploration op site arrest post op bleeding
G601.00	Subarachnoid haemorrhage from carotid siphon and bifurcation
D305100	Haemorrhagic disorder due to hyperheparinaemia
J121300	Chronic duodenal ulcer with haemorrhage and perforation
S751100	Spleen haematoma with open wound into cavity
C12y100	Haemorrhage of parathyroid
J110300	Acute gastric ulcer with haemorrhage and perforation
J12y300	Unspecified duodenal ulcer with haemorrhage and perforation
S623.00	Open traumatic subdural haemorrhage
J140100	Acute gastrojejunal ulcer with haemorrhage
S621.00	Open traumatic subarachnoid haemorrhage

ICD Code	ICD Term	Type
I85.0	Oesophageal varices with bleeding	gastrointestinal
K25.0	Gastric ulcer, acute with haemorrhage	gastrointestinal
K25.2	Gastric ulcer, acute with both haemorrhage and perforation	gastrointestinal
K25.4	Gastric ulcer, chronic or unspecified with haemorrhage	gastrointestinal
K25.6	Chronic or unspecified with both haemorrhage and perforation	gastrointestinal
K26.0	Duodenal ulcer, acute with haemorrhage	gastrointestinal
K26.2	Duodenal ulcer, acute with both haemorrhage and perforation	gastrointestinal
K26.4	Duodenal ulcer, chronic or unspecified with haemorrhage	gastrointestinal
K26.6	Chronic or unspecified with both haemorrhage and perforation	gastrointestinal
K27.0	Peptic ulcer, acute with haemorrhage	gastrointestinal
K27.2	Peptic ulcer, acute with both haemorrhage and perforation	gastrointestinal
K27.4	Peptic ulcer, chronic or unspecified with haemorrhage	gastrointestinal
K27.6	Chronic or unspecified with both haemorrhage and perforation	gastrointestinal
K28.0	Gastrojejunal ulcer, acute with haemorrhage	gastrointestinal
K28.2	Acute with both haemorrhage and perforation	gastrointestinal
K28.4	Gastrojejunal ulcer, chronic or unspecified with haemorrhage	gastrointestinal
K28.6	Chronic or unspecified with both haemorrhage and perforation	gastrointestinal
K29.0	Acute haemorrhagic gastritis	gastrointestinal
K62.5	Haemorrhage of anus and rectum	gastrointestinal
K66.1	Haemoperitoneum	gastrointestinal
K92.0	Haematemesis	gastrointestinal
K92.1	Melaena	gastrointestinal
K92.2	Gastrointestinal haemorrhage, unspecified	gastrointestinal
I60	Subarachnoid haemorrhage	intracranial bleed
I60.0	Subarachnoid haemorrhage from carotid siphon and bifurcation	intracranial bleed
I60.1	Subarachnoid haemorrhage from middle cerebral artery	intracranial bleed
I60.2	Subarachnoid haemorrhage from anterior communicating artery	intracranial bleed
I60.3	Subarachnoid haemorrhage from posterior communicating artery	intracranial bleed
I60.4	Subarachnoid haemorrhage from basilar artery	intracranial bleed
I60.5	Subarachnoid haemorrhage from vertebral artery	intracranial bleed
I60.6	Subarachnoid haemorrhage from other intracranial arteries	intracranial bleed
I60.7	Subarachnoid haemorrhage from intracranial artery, unspec	intracranial bleed
I60.8	Other subarachnoid haemorrhage	intracranial bleed
I60.9	Subarachnoid haemorrhage, unspecified	intracranial bleed
I61	Intracerebral haemorrhage	intracranial bleed

I61.0	Intracerebral haemorrhage in hemisphere, subcortical	intracranial bleed
I61.1	Intracerebral haemorrhage in hemisphere, cortical	intracranial bleed
I61.2	Intracerebral haemorrhage in hemisphere, unspecified	intracranial bleed
I61.3	Intracerebral haemorrhage in brain stem	intracranial bleed
I61.4	Intracerebral haemorrhage in cerebellum	intracranial bleed
I61.5	Intracerebral haemorrhage, intraventricular	intracranial bleed
I61.6	Intracerebral haemorrhage, multiple localized	intracranial bleed
I61.8	Other intracerebral haemorrhage	intracranial bleed
I61.9	Intracerebral haemorrhage, unspecified	intracranial bleed
I62	Other nontraumatic intracranial haemorrhage	intracranial bleed
I62.0	Subdural haemorrhage (acute)(nontraumatic)	intracranial bleed
I62.1	Nontraumatic extradural haemorrhage	intracranial bleed
I62.9	Intracranial haemorrhage (nontraumatic), unspecified	intracranial bleed
I69.0	Sequelae of subarachnoid haemorrhage	intracranial bleed
I69.1	Sequelae of intracerebral haemorrhage	intracranial bleed
I69.2	Sequelae of other nontraumatic intracranial haemorrhage	intracranial bleed
S06.4	Epidural haemorrhage	intracranial bleed
