# Supplementary material for: Comprehensive ascertainment of bleeding in patients prescribed different combinations of dual antiplatelet therapy (DAPT) and triple therapy (TT) in the UK: study protocol for three population-based cohort studies emulating ‘target trials’ (the ADAPTT Study)
Source: BMJ Open. 2019 Jun 4;9(6):e029388. doi: 10.1136/bmjopen-2019-029388 (PMC6561407; doi:10.1136/bmjopen-2019-029388)
Supplement: Supplementary file 2 [file bmjopen-2019-029388supp002.pdf]

Appendix 2: ADAPTT literature searches

DATABASE	Set 1. POPULATION + INTERVENTION + STUDIES *(RCTs/Cohort - **Dual/Triple antiplatelet therapy***	Set 2. POPULATION + INTERVENTION + OUTCOME *(bleeding)*	Set 3. POPULATION + OUTCOME + RISK *Risk of bleeding after a coronary intervention*
**CENTRAL** (database of controlled studies: RCTs, CCTs, ITS, CBA)	**775**	Included in Set 1 (CENTRAL database)	**720**
**MEDLINE**	**1822**	**558** (de-duplicated against Set 1 MEDLINE)	**5001**
**Embase**	**1156**	**520** (de-duplicated against Set 1 EMBASE)	**1582**
Total=	**3753**	**1078**	**7303**
After de-duplication=	**2544**	**849**	**6273**

**Set 3 (P-O-R)**	**Search-within-a-search-1**:<br>(score or scores or model or models or tool or tools or algorithm* or prognosis or predict or prediction or cohort):ti,ab n=1843
	**Search-within-a-search-2:**<br>(risk near3 (score* or factor or factors or model or models or prediction or stratification or category or bleed*)):ti,ab n=3300 (prior to de-duplication)

1. OVID MEDLINE

[Epub Ahead of Print, In-Process & Other Non-Indexed Citations, Ovid MEDLINE(R) Daily and Ovid MEDLINE(R) 1946 to Present]

1. Acute Coronary Syndrome/

2. (acute coronary adj3 syndrome*).ti,ab,kf.

3. ACS.ti,ab,kf.

4. heart attack*1.ti,ab,kf.

5. exp Myocardial Infarction/

6. myocardial infarct*.ti,ab,kf.

7. (MI or AMI).ti,ab,kf.

8. (stemi or non-stemi or nstemi).ti,ab,kf.

9. exp Angina, Unstable/

10. (angina adj3 unstable).ti,ab,kf.

11. exp Percutaneous Coronary Intervention/

12. (percutaneous coronary adj3 intervention).ti,ab,kf.

13. (PCI or PPCI or PCI-S).ti,ab,kf.

14. exp Angioplasty/

15. angioplasty.ti,ab,kf.

16. exp Stents/

17. stent*1.ti,ab,kf.

18. exp Coronary Artery Bypass/

19. CABG.ti,ab,kf.

20. coronary artery bypass.ti,ab,kf.

21. or/1-20 [Population]

22. (dual antiplatelet adj (therapy or treatment)).ti,ab,kf.

23. (DAPT or DAT).ti,ab,kf.

24. or/22-23

25. Aspirin/

26. (aspirin or acetylsalicylic acid or ASA).ti,ab,kf,rn,nm.

27. or/25-26

28. (clopidrogel or prasugrel or ticagrelor or plavix or efient or brilinta).ti,ab,kf,rn,nm,sh.

29. PURINERGIC P2Y RECEPTOR ANTAGONISTS/

30. (P2Y12 adj2 (antagonist* or inhibitor*)).ti,ab,kf,rn,nm.

31. or/28-30

32. 24 or (27 and 31)

33. exp Anticoagulants/

34. (anticoagul* or antithrombo* or anti-coagul* or anti-thrombo* or OAC* or DOAC* or NOAC*).ti,ab,kf,rn,nm.

35. (coumarin* or coumadin* or warfarin or marevan or dicoumarol or dicoumarin or dicumarin or dicumarol or acenocoumarol or phenindione or aldocumar or dabigatram or pradaxa or BIBR1048 or Apixaban or Eliquis or BMS-562247-01 or Edoxaban or Lixiana or

savaysa or DU-176b or betrixaban or PRT-054021 or PRT0504021 or rivaroxaban or xarelto or BAY-59739 or Erixaban or D0913).ti,ab,kf,rn,nm.

36. ((Vitamin K or Factor Xa or Factor 10a or Factor IIa) adj2 (antagonist* or inhibitor*)).ti,ab,kw,rn,nm.

37. or/33-36

38. (triple therapy or triple antiplatelet therapy or triple antithrombotic therapy or triple antithrombotic combination therapy).ti,ab,kf.

39. (TAPT or TOAT).ti,ab,kf.

40. ((24 or 31) and 37) or 38 or 39

41. 32 or 40 [Intervention]

42. (bleed*1 or bleeding).ti,ab,kf.

43. Hemorrhage/

44. (hemorrhag* or haemorrhag*).ti,ab,kf.

45. or/42-44 [Outcome]

46. risk/ or risk assessment/ or risk factors/

47. risk stratification.ti,ab,kf.

48. (risk adj3 model*).ti,ab,kf.

49. risk factor*.ti,ab,kf.

50. or/46-49 [Risk]

51. randomized controlled trial.pt.

52. controlled clinical trial.pt.

53. (RCT or randomi*).ti,ab,kf.

54. placebo.ab.

55. (random* adj (assign* or allocat* or divide* or division)).ti,ab,kf.

56. trial.ti,ab.

57. groups.ab.

58. or/51-57

59. cohort studies/ or follow-up studies/ or longitudinal studies/ or prospective studies/ or retrospective studies/

60. longitudinal.ab.

61. (prospective or retrospective).ab.

62. (CCT or (control* adj (trial*1 or study or studies))).ti,ab,kf.

63. (Follow up adj2 (study or studies)).ti,ab,kf.

64. follow up assessment.ti,ab,kf.

65. (compar* and group*).ab.

66. cohort.ti,ab,kf.

67. (register or registry).ti,ab,kf.

68. or/59-67

69. 58 or 68 **[Study Design Filter]**

70. 21 and 41 and 69

71. 21 and 41 and 45

72. 21 and 41 and 50

73. 21 and 41 and 45 and 50

74. 21 and 45 and 50

2. The Cochrane Library, Issue 7, 2016

ID	Search
#1	MeSH descriptor: [Acute Coronary Syndrome] explode all trees
#2	"acute coronary syndrome":ti,ab,kw (Word variations have been searched)
#3	ACS:ab (Word variations have been searched)
#4	heart attack*:ti,ab,kw (Word variations have been searched)
#5	MeSH descriptor: [Myocardial Infarction] explode all trees
#6	myocardial next infarct*:ti,ab,kw (Word variations have been searched)
#7	MI or AMI:ab (Word variations have been searched)
#8	(stemi or non-stemi or nstemi):ti,ab,kw (Word variations have been searched)
#9	MeSH descriptor: [Angina, Unstable] explode all trees
#10	(angina near unstable):ti,ab,kw (Word variations have been searched)
#11	MeSH descriptor: [Percutaneous Coronary Intervention] explode all trees
#12	(percutaneous next coronary) and intervention:ti,ab,kw (Word variations have been searched)
#13	PCI or PPCI or PCI-S:ab (Word variations have been searched)
#14	MeSH descriptor: [Angioplasty] explode all trees
#15	angioplasty:ti,ab,kw (Word variations have been searched)
#16	MeSH descriptor: [Stents] explode all trees

#17 stent or stents or stenting:ti,ab,kw (Word variations have been searched)

#18 MeSH descriptor: [Coronary Artery Bypass] explode all trees

#19 CABG:ab (Word variations have been searched)

#20 "coronary artery bypass":ti,ab,kw (Word variations have been searched)

#21 (#1 or #2 or #3 or #4 or #5 or #6 or #7 or #8 or #9 or #10 or #11 or #12 or #13 or #14 or #15 or #16 or #17 or #18 or #19 or #20)

#22 (dual next antiplatelet) and (therapy or treatment):ti,ab,kw (Word variations have been searched)

#23 (DAPT or DAT):ti,ab,kw (Word variations have been searched)

#24 #22 or #23

#25 MeSH descriptor: [Aspirin] explode all trees

#26 aspirin or "acetylsalicylic acid":ti,ab,kw (Word variations have been searched)

#27 ASA:ab (Word variations have been searched)

#28 #25 or #26 or #27

#29 (clopidrogel or prasugrel or ticagrelor or plavix or efient or brilinta):ti,ab,kw (Word variations have been searched)

#30 MeSH descriptor: [Purinergic P2Y Receptor Antagonists] explode all trees

#31 (P2Y12 near (antagonist* or inhibitor*)):ti,ab,kw (Word variations have been searched)

#32 #29 or #30 or #31

#33 #24 or (#28 and #32)

#34 MeSH descriptor: [Anticoagulants] explode all trees

#35 (anticoagul* or antithrombo* or anti-coagul* or anti-thrombo* or OAC* or DOAC* or NOAC*):ti,ab,kw (Word variations have been searched)

#36 (coumarin* or coumadin* or warfarin or marevan or dicoumarol or dicoumarin or dicumarin or dicumarol or acenocoumarol or phenindione or aldocumar):ti,ab,kw (Word variations have been searched)

#37 dabigatram or pradaxa or BIBR1048 or Apixaban or Eliquis or BMS-562247-01 or Edoxaban or Lixiana or savaysa or DU-176b or betrixaban or PRT-054021 or PRT0504021 or rivaroxaban or xarelto or BAY-59739 or Erixaban or D0913:ti,ab,kw (Word variations have been searched)

#38 "vitamin K" and (antagonist* or inhibitor*):ti,ab,kw (Word variations have been searched)

#39 ("vitamin K" or "factor Xa" or "factor 10a" or "factor IIa") and (antagonist* or inhibitor*):ti,ab,kw (Word variations have been searched)

#40 #34 or #35 or #36 or #37 or #38 or #39

#41 triple near therapy:ti,ab,kw (Word variations have been searched)

#42 TAPT or TOAT:ab (Word variations have been searched)

#43 ((#24 or #32) and #40) or #41 or #42

#44 #33 or #43

#45 MeSH descriptor: [Hemorrhage] explode all trees

#46 bleed*:ti,ab,kw (Word variations have been searched)

#47 hemorrhag* or haemorrhag*:ti,ab,kw (Word variations have been searched)

#48 #45 or #46 or #47

#49 MeSH descriptor: [Risk] explode all trees

#50 "risk stratification":ti,ab,kw (Word variations have been searched)

#51 risk near (factor* or model*):ti,ab,kw (Word variations have been searched)

#52 #49 or #50 or #51

#53 #21 and #44

#54 #21 and #48 and #52

#55 (#54 not #53)

3. OVID EMBASE (1974 to date)

1. exp acute coronary syndrome/
2. (acute coronary adj3 syndrome*).ti,ab,kw.
3. ACS.ti,ab,kw.
4. heart attack*1.ti,ab,kw.
5. exp heart infarction/
6. myocardial infarct*.ti,ab,kw.
7. (MI or AMI).ti,ab,kw.
8. (stemi or non-stemi or nstemi).ti,ab,kw.
9. exp unstable angina pectoris/
10. (angina adj3 unstable).ti,ab,kw.
11. exp percutaneous coronary intervention/
12. (percutaneous coronary adj3 intervention).ti,ab,kw.
13. (PCI or PPCI or PCI-S).ti,ab,kw.
14. exp angioplasty/
15. angioplasty.ti,ab,kw.
16. exp stent/
17. stent*1.ti,ab,kw.

18. coronary artery bypass graft/

19. CABG.ti,ab,kw.

20. coronary artery bypass.ti,ab,kw.

21. or/1-20

22. (dual antiplatelet adj (therapy or treatment)).ti,ab,kw.

23. (DAPT or DAT).ti,ab,kw.

24. or/22-23

25. acetylsalicylic acid/

26. (aspirin or acetylsalicylic acid or ASA).ti,ab,kw,rn,tn.

27. or/25-26

28. (clopidrogel or prasugrel or ticagrelor or plavix or efient or brilinta).ti,ab,kw,rn,tn,sh.

29. antithrombocytic agent/

30. (P2Y12 adj2 (antagonist* or inhibitor*)).ti,ab,kw,rn.

31. exp purinergic receptor blocking agent/

32. or/28-31

33. 24 or (27 and 32)

34. exp anticoagulant agent/

35. (anticoagul* or antithrombo* or anti-coagul* or anti-thrombo* or OAC* or DOAC* or NOAC*).ti,ab,kw.

36. (coumarin* or coumadin* or warfarin or marevan or dicoumarol or dicoumarin or dicumarin or dicumarol or acenocoumarol or phenindione or aldocumar).ti,ab,kw,rn,tn.

37. (dabigatram or pradaxa or BIBR1048 or Apixaban or Eliquis or BMS-562247-01 or Edoxaban or Lixiana or savaysa or DU-176b or betrixaban or PRT-054021 or PRT0504021 or rivaroxaban or xarelto or BAY-59739 or Erixaban or D0913).ti,ab,kw,rn,tn.

38. (vitamin K adj2 (antagonist$ or inhibitor$)).ti,ab,kw,rn.

39. (factor Xa adj2 (antagonist$ or inhibitor$)).ti,ab,kw,rn.

40. (factor 10a adj2 (antagonist$ or inhibitor$)).ti,ab,kw,rn.

41. (factor IIa adj2 (antagonist$ or inhibitor$)).ti,ab,kw,rn.

42. (adjunct* or combin* or concurrent or cotherap* or co-therap* or dual or plus or triple).ti,ab,kw.

43. drug combination/

44. or/34-43

45. (triple therapy or triple antiplatelet therapy or triple antithrombotic therapy or triple antithrombotic combination therapy).ti,ab,kw.

46. (TAPT or TOAT).ti,ab,kw.

47. ((24 or 32) and 44) or 45 or 46

48. 33 or 47

49. (bleed*1 or bleeding).ti,ab,kw.

50. exp Bleeding/

51. (hemorrhag* or haemorrhag*).ti,ab,kw.

52. or/49-51

53. risk assessment/ or risk factor/ or patient risk/ or risk/ or high risk patient/

54. risk stratification.ti,ab,kw.

55. (risk adj3 model*).ti,ab,kw.

56. risk factor*.ti,ab,kw.

57. or/53-56

58. Randomized Controlled Trial/

59. Randomization/

60. (random* adj (assign* or allocat* or divide* or division)).ti,ab,kw.

61. (RCT or randomi*).ti,ab,kw.

62. trial.ti,ab.

63. placebo.ti,ab,kw.

64. ((assign$ or match or matched or allocation) adj5 (alternate or group$1 or intervention$1 or patient$1 or subject$1 or participant$1)).ti,ab,kw.

65. double blind procedure/

66. ((double or single or doubly or singly) adj (blind or blinded or blindly)).ti.

67. or/58-66

68. Controlled Clinical Trial/

69. (CCT or (controlled adj7 (study or design or trial))).ti,ab,kw.

70. cohort analysis/

71. cohort.ti,ab,kw.

72. longitudinal.ab.

73. (prospective or retrospective).ab.

74. follow up assessment.ti,ab,kw.

75. clinical trial/ or multicenter study/ or phase 2 clinical trial/ or phase 3 clinical trial/ or phase 4 clinical trial/

76. clinical study/ or exp longitudinal study/ or major clinical study/ or prospective study/ or retrospective study/

77. (Follow up adj2 study).ti,ab,kw.

78. register.ti,ab,kw.

79. or/68-78

80. 67 or 79

81. Animal experiment/ not (human experiment/ or human/)

82. (rat or rats or mouse or mice or swine or porcine or murine or sheep or lambs or pigs or piglets or rabbit or rabbits or cat or cats or dog or dogs or cattle or bovine or monkey or monkeys or trout or marmoset$1).ti. and animal experiment/

83. or/81-82

84. 80 not 83

85. 21 and 48 and 84

86. limit 85 to exclude medline journals

87. **21 and 48 and 52**

88. limit 87 to exclude medline journals

89. **21 and 48 and 57**

90. limit 89 to exclude medline journals

91. **21 and 48 and 52 and 57**

92. 86 or 88 or 90 or 91
