# Supplementary material for: Comprehensive ascertainment of bleeding in patients prescribed different combinations of dual antiplatelet therapy (DAPT) and triple therapy (TT) in the UK: study protocol for three population-based cohort studies emulating ‘target trials’ (the ADAPTT Study)
Source: BMJ Open. 2019 Jun 4;9(6):e029388. doi: 10.1136/bmjopen-2019-029388 (PMC6561407; doi:10.1136/bmjopen-2019-029388)
Supplement: Supplementary file 3 [file bmjopen-2019-029388supp003.pdf]

Appendix 3: List of product codes in Clinical Practice Research Datalink (CPRD). All for antiplatelet agents (aspirin, clopidogrel, prasugrel and ticagrelor) and anticoagulants (warfarin, dabigatran, rivaroxaban, apixaban)

Antiplatelet agents (aspirin, clopidogrel, prasugrel and ticagrelor)

Gemscript product code	Product name	Category
3	Aspirin 75mg dispersible tablets	Aspirin
16	Aspirin 75mg tablets	Aspirin
34	Aspirin 75mg gastro-resistant tablets	Aspirin
111	ASPIRIN 40 MG CAP	Aspirin
216	ASPIRIN 70 MG TAB	Aspirin
254	Aspirin 300mg tablets	Aspirin
377	Aspirin 300mg dispersible tablets	Aspirin
383	ASPIRIN 60 MG TAB	Aspirin
393	Disprin 300mg dispersible tablets (Reckitt Benckiser Healthcare (UK) Ltd)	Aspirin
395	Aspirin mixture	Aspirin
434	Aspirin 300mg gastro-resistant tablets	Aspirin
1137	Nu-seals aspirin ec 300mg Gastro-resistant tablet (Eli Lilly and Company Ltd)	Aspirin
1486	ASPIRIN 75 MG SUP	Aspirin
2105	Solprin 300mg Tablet (Reckitt Benckiser Healthcare (UK) Ltd)	Aspirin
2607	Paynocil Tablet (Beecham Research Laboratories)	Aspirin
2628	Nu-seals aspirin ec 75mg Gastro-resistant tablet (Eli Lilly and Company Ltd)	Aspirin
2754	ASPIRIN SOLUBLE 150 MG TAB	Aspirin
2924	ASPIRIN 150 MG TAB	Aspirin
4271	ASPIRIN SOLUBLE 200 MG TAB	Aspirin
4523	ASPIRIN 50 MG CAP	Aspirin
6006	Nu-Seals 75 gastro-resistant tablets (Alliance Pharmaceuticals Ltd)	Aspirin
6007	Nu-Seals 300 gastro-resistant tablets (Alliance Pharmaceuticals Ltd)	Aspirin
6696	Micropirin 75mg gastro-resistant tablets (Dexcel-Pharma Ltd)	Aspirin
7417	ASPIRIN 40 MG TAB	Aspirin
7462	ASPIRIN 325 MG CAP	Aspirin
7486	ASPIRIN 37.5 MG TAB	Aspirin
7516	Aspirin 300mg effervescent tablets sugar free	Aspirin
7665	ASPIRIN SR 300 MG TAB	Aspirin
7915	ASPIRIN SR 100 MG TAB	Aspirin
7944	ASPIRIN SOLUBLE 40 MG CAP	Aspirin
8185	Disprin CV 300mg modified-release tablets (Reckitt Benckiser Healthcare (UK) Ltd)	Aspirin
8186	Aspirin 300mg modified-release tablets	Aspirin

8424	ASPIRIN PAED 81 MG TAB	Aspirin
8645	Aspirin 300mg effervescent tablets	Aspirin
8733	JUNIOR ASPIRIN 37.5 MG TAB	Aspirin
8734	ASPIRIN disp 37.5 MG TAB	Aspirin
8843	ASPIRIN 325 MG TAB	Aspirin
9027	ASPIRIN disp 150 MG TAB	Aspirin
9144	Caprin 75mg gastro-resistant tablets (Wockhardt UK Ltd)	Aspirin
9301	Aspirin 100mg modified-release tablets	Aspirin
10305	Aspirin 162.5mg capsules	Aspirin
10310	Aspirin powder	Aspirin
11941	ASPIRIN SACHETS 30 MG	Aspirin
11977	Aspro clear maximum strength tablets	Aspirin
12102	ASPIRIN SOLUBLE 100 MG TAB	Aspirin
13882	Imazin XL tablets (Napp Pharmaceuticals Ltd)	Aspirin
15397	ASPIRIN SOLUBLE 50 MG TAB	Aspirin
15517	ASPIRIN 100 MG SUP	Aspirin
17704	Platet 100mg Effervescent tablet (Roche Products Ltd)	Aspirin
17920	Disprin cv 100mg Modified-release tablet (Reckitt Benckiser Healthcare (UK) Ltd)	Aspirin
18030	Imazin XL forte tablets (Napp Pharmaceuticals Ltd)	Aspirin
18217	Aspirin 300mg orodispersible tablets sugar free	Aspirin
18329	Enprin 75mg gastro-resistant tablets (Galpharm International Ltd)	Aspirin
19189	Micropirin 75mg Gastro-resistant tablet (Ratiopharm UK Ltd)	Aspirin
19577	NU-SEALS ASPIRIN	Aspirin
19674	ASPIRIN DISPERSIBLE	Aspirin
19797	NU-SEALS ASPIRIN	Aspirin
19813	ASPIRIN SOLUBLE	Aspirin
20206	ASPIRIN 50 MG SUP	Aspirin
20840	Acetylsalicylic acid mix	Aspirin
21380	Aspirin 75mg / Isosorbide mononitrate 60mg modified-release tablets	Aspirin
21382	Aspirin 150mg / Isosorbide mononitrate 60mg modified-release tablets	Aspirin
21921	Postmi ec 300mg Gastro-resistant tablet (Ashbourne Pharmaceuticals Ltd)	Aspirin
22107	ASPIRIN disp 200 MG TAB	Aspirin
22138	Aspirin 324mg modified-release tablets	Aspirin
22232	Disprin Direct 300mg orodispersible tablets (Reckitt Benckiser Healthcare (UK) Ltd)	Aspirin
22618	Solprin 75mg Tablet (Reckitt Benckiser Healthcare (UK) Ltd)	Aspirin
22864	ASPIRIN PAED MIX	Aspirin
23488	Claradin 300mg Tablet (Nicholas Laboratories Ltd)	Aspirin
23495	ASPIRIN	Aspirin
23593	PostMI 75 dispersible tablets (Ashbourne Pharmaceuticals Ltd)	Aspirin
23878	Nu-seals cardio ec 75mg Gastro-resistant tablet (Genus Pharmaceuticals Ltd)	Aspirin

23932	Aspro Clear 300mg effervescent tablets (Bayer Plc)	Aspirin
24025	Caprin 300mg gastro-resistant tablets (Pinewood Healthcare)	Aspirin
24960	Aspirin 300mg tablets (Vantage)	Aspirin
25335	PostMI 75 EC tablets (Ashbourne Pharmaceuticals Ltd)	Aspirin
25718	Angettes 75 tablets (Bristol-Myers Squibb Pharmaceuticals Ltd)	Aspirin
27467	ASPIRIN SOLUBLE 400 MG TAB	Aspirin
28707	ASPIRIN M/F 324 MG TAB	Aspirin
29515	ACETYLSALICYLIC ACID	Aspirin
29759	Aspro Tablet (Roche Consumer Health)	Aspirin
29848	Aspirin 300mg with Glycine 150mg chewable tablets	Aspirin
30920	Aspirin 300mg Dispersible tablet (M & A Pharmachem Ltd)	Aspirin
31210	Aspirin 300mg Tablet (Co-operative)	Aspirin
31211	Aspirin 75mg Dispersible tablet (A A H Pharmaceuticals Ltd)	Aspirin
31858	Caspac xl 162.5mg Capsule (Pharmacia Ltd)	Aspirin
31870	Aspirin 320mg tablets	Aspirin
31938	Aspirin 75mg gastro-resistant tablets (Sandoz Ltd)	Aspirin
31953	Aspirin 75mg dispersible tablets (IVAX Pharmaceuticals UK Ltd)	Aspirin
31954	Aspirin 75mg dispersible tablets (Teva UK Ltd)	Aspirin
31956	Aspirin 75mg gastro-resistant tablets (Kent Pharmaceuticals Ltd)	Aspirin
32036	Aspirin 75mg dispersible tablets (Actavis UK Ltd)	Aspirin
32210	Aspirin 300mg dispersible tablets (Actavis UK Ltd)	Aspirin
32992	Aspirin 75mg gastro-resistant tablets (Mylan)	Aspirin
33293	Aspirin 75mg gastro-resistant tablets (Sterwin Medicines)	Aspirin
33320	Aspirin 75mg Dispersible tablet (Sovereign Medical Ltd)	Aspirin
33656	Aspirin 75mg dispersible tablets (A A H Pharmaceuticals Ltd)	Aspirin
33662	Aspirin 300mg Dispersible tablet (A A H Pharmaceuticals Ltd)	Aspirin
33668	Aspirin 300mg Dispersible tablet (Rusco Ltd)	Aspirin
33676	Aspirin 75mg dispersible tablets (Kent Pharmaceuticals Ltd)	Aspirin
34309	Aspirin 300mg dispersible tablets (A A H Pharmaceuticals Ltd)	Aspirin
34385	Aspirin 75mg Soluble tablet (Co-operative)	Aspirin
34386	Aspirin 300mg tablets (Actavis UK Ltd)	Aspirin
34434	Aspirin 75mg dispersible tablets (Thornton & Ross Ltd)	Aspirin
34485	Aspirin 75mg gastro-resistant tablets (IVAX Pharmaceuticals UK Ltd)	Aspirin
34611	Aspirin 75mg gastro-resistant tablets (C P Pharmaceuticals Ltd)	Aspirin
34666	Aspirin ec 300mg Gastro-resistant tablet (A A H Pharmaceuticals Ltd)	Aspirin
34762	Aspirin 300mg Gastro-resistant tablet (Galen Ltd)	Aspirin
34796	Aspirin 75mg Gastro-resistant tablet (Galen Ltd)	Aspirin
34797	Aspirin 75mg gastro-resistant tablets (Actavis UK Ltd)	Aspirin
34942	Aspirin 75mg Dispersible tablet (Nucare Plc)	Aspirin
36543	Aspirin 100mg effervescent tablets	Aspirin
37541	Aspirin 227mg medicated chewing-gum	Aspirin
39738	Aspirin 162.5mg modified-release capsules	Aspirin
40144	Aspirin 300mg Dispersible tablet (Thornton & Ross Ltd)	Aspirin

40381	Aspirin 75mg Soluble tablet (C P Pharmaceuticals Ltd)	Aspirin
41512	Aspirin 75mg gastro-resistant tablets (Teva UK Ltd)	Aspirin
41569	Aspirin 300mg tablets (A A H Pharmaceuticals Ltd)	Aspirin
41594	Aspirin 300mg Dispersible tablet (Teva UK Ltd)	Aspirin
42061	ASPIRIN 65 MG SUP	Aspirin
43060	Aspirin 300mg Soluble tablet (Celltech Pharma Europe Ltd)	Aspirin
43434	Aspirin 300mg gastro-resistant tablets (A A H Pharmaceuticals Ltd)	Aspirin
43679	Flamasacard 162.5mg Modified-release capsule (Abbey Pharmaceuticals Ltd)	Aspirin
43709	Aspirin 75mg gastro-resistant tablets (Almus Pharmaceuticals Ltd)	Aspirin
43806	Aspirin 300mg gastro-resistant tablets (Sandoz Ltd)	Aspirin
44639	Aspirin 300mg Dispersible tablet (Nucare Plc)	Aspirin
45643	Aspirin 75mg Soluble tablet (Celltech Pharma Europe Ltd)	Aspirin
45840	Aspirin 300mg Dispersible tablet (Numark Management Ltd)	Aspirin
45851	Aspirin 300mg Soluble tablet (Ranbaxy (UK) Ltd)	Aspirin
47937	Aspirin 75mg dispersible tablets (Wockhardt UK Ltd)	Aspirin
47992	Aspirin 75mg gastro-resistant tablets (A A H Pharmaceuticals Ltd)	Aspirin
48000	Aspirin 300mg tablets (Sigma Pharmaceuticals Plc)	Aspirin
48021	Aspirin 75mg Tablet (Hillcross Pharmaceuticals Ltd)	Aspirin
48165	Aspirin 300mg tablets (Aspar Pharmaceuticals Ltd)	Aspirin
48974	Aspirin 75mg tablets (Phoenix Healthcare Distribution Ltd)	Aspirin
49060	Aspirin 75mg dispersible tablets (Alliance Healthcare (Distribution) Ltd)	Aspirin
49220	Aspirin 300mg tablets (Kent Pharmaceuticals Ltd)	Aspirin
49685	Aspirin 75mg dispersible tablets (Sigma Pharmaceuticals Plc)	Aspirin
50555	Aspirin 300mg dispersible tablets (DE Pharmaceuticals)	Aspirin
50926	Aspirin 75mg dispersible tablets (The Boots Company Plc)	Aspirin
50949	Aspirin 75mg tablets (A A H Pharmaceuticals Ltd)	Aspirin
51561	Aspirin 75mg gastro-resistant tablets (Zanza Laboratories Ltd)	Aspirin
52044	Aspirin 300mg caplets (The Boots Company Plc)	Aspirin
52280	Aspirin 300mg Tablet (Wockhardt UK Ltd)	Aspirin
52618	Aspirin 75mg dispersible tablets (Bristol Laboratories Ltd)	Aspirin
52905	Aspirin 300mg tablets (Lloyds Pharmacy Ltd)	Aspirin
53178	Aspirin 75mg gastro-resistant tablets (Wockhardt UK Ltd)	Aspirin
53622	Aspirin 300mg Tablet (M & A Pharmachem Ltd)	Aspirin
53711	Aspirin 300mg Tablet (Nucare Plc)	Aspirin
53791	Aspirin 150mg suppositories (Alliance Healthcare (Distribution) Ltd)	Aspirin
53804	Aspirin 300mg gastro-resistant tablets (Alliance Healthcare (Distribution) Ltd)	Aspirin
53816	Aspirin 300mg dispersible tablets (Alliance Healthcare (Distribution) Ltd)	Aspirin
54284	Aspirin 75mg dispersible tablets (Almus Pharmaceuticals Ltd)	Aspirin
54430	Aspirin 75mg tablets (Alliance Healthcare (Distribution) Ltd)	Aspirin
54526	Aspirin 300mg tablets (Alliance Healthcare (Distribution) Ltd)	Aspirin

54565	Aspirin 75mg dispersible tablets (Lloyds Pharmacy Ltd)	Aspirin
54734	Aspirin 300mg tablets (Wockhardt UK Ltd)	Aspirin
54997	Aspirin 75mg dispersible tablets (Dowelhurst Ltd)	Aspirin
55230	Aspirin 300mg dispersible tablets (Kent Pharmaceuticals Ltd)	Aspirin
55579	Aspirin 300mg tablets (Almus Pharmaceuticals Ltd)	Aspirin
56007	Aspirin 300mg dispersible tablets (Sigma Pharmaceuticals Plc)	Aspirin
56736	Aspirin 300mg tablets (Waymade Healthcare Plc)	Aspirin
56883	Aspirin 75mg tablets (Waymade Healthcare Plc)	Aspirin
56995	Aspirin 75mg dispersible tablets (Phoenix Healthcare Distribution Ltd)	Aspirin
56996	Aspirin 75mg dispersible tablets (Waymade Healthcare Plc)	Aspirin
57057	Aspirin 75mg dispersible tablets (Wockhardt UK Ltd)	Aspirin
58331	Aspirin 300mg gastro-resistant tablets (Mylan)	Aspirin
59021	Aspirin 75mg gastro-resistant tablets (Bristol Laboratories Ltd)	Aspirin
59244	Aspirin 100mg capsules	Aspirin
59253	Aspirin 75mg gastro-resistant tablets (Waymade Healthcare Plc)	Aspirin
59728	Aspirin 75mg tablets (Alissa Healthcare Research Ltd)	Aspirin
59791	Aspirin 75mg dispersible tablets (Aspar Pharmaceuticals Ltd)	Aspirin
60127	Aspirin 75mg tablets (DE Pharmaceuticals)	Aspirin
60278	Aspirin 300mg tablets (DE Pharmaceuticals)	Aspirin
60693	Aspirin 15mg/5ml oral solution	Aspirin
60694	Aspirin 25mg/5ml oral solution	Aspirin
60777	Aspirin 75mg gastro-resistant tablets (DE Pharmaceuticals)	Aspirin
62334	Aspirin 300mg caplets (Wockhardt UK Ltd)	Aspirin
62430	Aspirin 300mg suppositories (A A H Pharmaceuticals Ltd)	Aspirin
63603	Laboprin Tablet (Laboratories For Applied Biology Ltd)	Aspirin
64071	Aspirin powder (J M Loveridge Ltd)	Aspirin
65027	Bisoprolol 5mg / Aspirin 100mg capsules	Aspirin
66345	Aspirin 75mg dispersible tablets (DE Pharmaceuticals)	Aspirin
66546	Aspirin 75mg dispersible tablets (Numark Ltd)	Aspirin
66563	Aspirin 75mg gastro-resistant tablets (Phoenix Healthcare Distribution Ltd)	Aspirin
66861	Aspirin 75mg effervescent tablets	Aspirin
67124	Bisoprolol 10mg / Aspirin 75mg capsules	Aspirin
67160	Aspirin 300mg dispersible tablets (Lloyds Pharmacy Ltd)	Aspirin
67362	Aspirin 300mg suppositories (Alliance Healthcare (Distribution) Ltd)	Aspirin
67521	Aspirin 15mg/5ml oral suspension	Aspirin
67754	Aspirin 300mg dispersible tablets (Almus Pharmaceuticals Ltd)	Aspirin
67858	Aspirin 25mg capsules	Aspirin
68051	Aspirin 150mg suppositories (Colorama Pharmaceuticals Ltd)	Aspirin
68752	Aspirin 75mg tablets (Sigma Pharmaceuticals Plc)	Aspirin
70549	Danamep 75mg dispersible tablets (Ecogen Europe Ltd)	Aspirin
70841	Aspirin 300mg Dispersible tablet (Family Health)	Aspirin
71078	Aspirin 300mg dispersible tablets (Mawdsley-Brooks & Company	Aspirin

	Ltd)	
71192	Aspirin 75mg tablets (Kent Pharmaceuticals Ltd)	Aspirin
489	Clopidogrel 75mg tablets	Clopidogrel
836	Plavix 75mg tablets (Sanofi)	Clopidogrel
17816	PLAVIX FC	Clopidogrel
17817	CLOPIDOGREL FC	Clopidogrel
38349	Clopidogrel 300mg tablets	Clopidogrel
38998	Plavix 300mg tablets (Sanofi)	Clopidogrel
40913	Grepid 75mg tablets (Kent Pharmaceuticals Ltd)	Clopidogrel
42750	Clopidogrel 75mg tablets (Actavis UK Ltd)	Clopidogrel
45905	Clopidogrel 1mg/ml oral suspension	Clopidogrel
46891	Clopidogrel 75mg/5ml oral suspension	Clopidogrel
52761	Clopidogrel 75mg tablets (Dr Reddy's Laboratories (UK) Ltd)	Clopidogrel
53751	Clopidogrel 75mg tablets (Phoenix Healthcare Distribution Ltd)	Clopidogrel
54700	Clopidogrel 75mg tablets (A A H Pharmaceuticals Ltd)	Clopidogrel
55161	Clopidogrel 75mg tablets (Wockhardt UK Ltd)	Clopidogrel
56807	Clopidogrel 75mg tablets (Teva UK Ltd)	Clopidogrel
57036	Clopidogrel 75mg tablets (Mylan)	Clopidogrel
58347	Clopidogrel 75mg tablets (DE Pharmaceuticals)	Clopidogrel
58448	Clopidogrel 75mg tablets (Aspire Pharma Ltd)	Clopidogrel
59904	Clopidogrel 75mg/5ml oral solution	Clopidogrel
62855	Clopidogrel 75mg tablets (Alliance Healthcare (Distribution) Ltd)	Clopidogrel
62978	Clopidogrel 75mg tablets (Sandoz Ltd)	Clopidogrel
63450	Clopidogrel 75mg tablets (Almus Pharmaceuticals Ltd)	Clopidogrel
65909	Clopidogrel 75mg tablets (Milpharm Ltd)	Clopidogrel
67037	Clopidogrel 75mg tablets (Zentiva)	Clopidogrel
39932	Prasugrel 10mg tablets	Prasugrel
40114	Prasugrel 5mg tablets	Prasugrel
40591	Efient 5mg tablets (Eli Lilly and Company Ltd)	Prasugrel
41229	Efient 10mg tablets (Eli Lilly and Company Ltd)	Prasugrel
45576	Ticagrelor 90mg tablets	Ticagrelor
47895	Brilique 90mg tablets (AstraZeneca UK Ltd)	Ticagrelor
66973	Ticagrelor 60mg tablets	Ticagrelor
68710	Brilique 60mg tablets (AstraZeneca UK Ltd)	Ticagrelor
70606	Ticagrelor 90mg orodispersible tablets sugar free	Ticagrelor

Anticoagulants (warfarin, dabigatran, rivaroxaban, apixaban)

Gemscript product code	Product name
45	Warfarin 1mg tablets
61	Warfarin 3mg tablets
833	Warfarin 3mg/5ml oral solution
1781	Warfarin 5mg tablets
2675	Fragmin 10,000units/4ml solution for injection ampoules (Pfizer Ltd)
2676	Fragmin 5,000units/0.2ml solution for injection pre-filled syringes (Pfizer Ltd)
2677	Clexane 100mg/ml Injection (Aventis Pharma)
3744	Heparin 10 iu/ml Flush solution
3895	Heparin sodium 1000iu/ml Injection
4446	Acenocoumarol 1mg tablets
4888	Heplok 10 iu/ml Oral solution (LEO Pharma)
4995	Enoxaparin 100mg/ml injection
5305	Sinthrome 1mg tablets (Merus Labs Luxco S.a R.L.)
5526	Fragmin 2,500units/0.2ml solution for injection pre-filled syringes (Pfizer Ltd)
5747	Fragmin 25,000iu/ml Solution for injection (Pfizer Ltd)
5998	Fragmin 10,000 iu/ml Solution for injection (Pfizer Ltd)
6262	Warfarin 500microgram tablets
6478	Enoxaparin sodium 20mg/0.2ml solution for injection pre-filled syringes
6695	Dalteparin sodium 2,500units/0.2ml solution for injection pre-filled syringes
6822	Elmiron 100mg capsules (Teva UK Ltd)
6860	Fragmin 15,000units/0.6ml solution for injection pre-filled syringes (Pfizer Ltd)
7154	Clexane Forte 120mg/0.8ml solution for injection pre-filled syringes (Sanofi)
7199	Enoxaparin sodium 40mg/0.4ml solution for injection pre-filled syringes
7307	Clexane 40mg/0.4ml solution for injection pre-filled syringes (Sanofi)
7371	Clexane 100mg/1ml solution for injection pre-filled syringes (Sanofi)
8466	Marevan 1mg tablets (AMCo)
8467	Marevan 3mg tablets (AMCo)
8664	Heparin sodium 5000iu/ml Injection
9140	Dalteparin sodium 10,000units/4ml solution for injection ampoules
9593	Dalteparin 25000iu/ml injection solution
9605	Dalteparin sodium 5,000units/0.2ml solution for injection pre-filled syringes
9610	Tinzaparin 20000 iu/ml Injection
9640	Tinzaparin 10000 IU/ml Injection
10002	Dalteparin 10000iu/1ml injection solution
10004	Clexane 80mg/0.8ml solution for injection pre-filled syringes (Sanofi)
10044	Dalteparin sodium 10,000units/0.4ml solution for injection pre-filled syringes
10072	Fragmin 10,000units/0.4ml solution for injection pre-filled syringes (Pfizer Ltd)
10170	Dalteparin sodium 15,000units/0.6ml solution for injection pre-filled syringes
10194	Dalteparin sodium 12,500units/0.5ml solution for injection pre-filled syringes

10240	Tinzaparin sodium 14,000units/0.7ml solution for injection pre-filled syringes
10532	Minihep calcium 5000iu/0.2ml Injection (LEO Pharma)
10533	Calciparine 25,000iu/ml Injection (Sanofi-Synthelabo Ltd)
10560	WARFARIN 10 MG TAB
11372	Heparin 100iu/ml Flush solution
12681	Heparin calcium 25,000iu/ml Injection
12974	Clexane 150mg/ml Injection (Aventis Pharma)
13058	Enoxaparin 150mg/ml injection
13097	Clexane 20mg/0.2ml solution for injection pre-filled syringes (Sanofi)
13210	Enoxaparin sodium 80mg/0.8ml solution for injection pre-filled syringes
13270	Enoxaparin sodium 120mg/0.8ml solution for injection pre-filled syringes
13348	Marevan 5mg tablets (AMCo)
13501	Dindevan 50mg Tablet (Goldshield Pharmaceuticals Ltd)
13502	Dindevan 10mg Tablet (Goldshield Pharmaceuticals Ltd)
13503	Phenindione 50mg tablets
13504	Phenindione 25mg tablets
13505	Phenindione 10mg tablets
13568	Heparin sodium 25,000iu/ml Subcutaneous injection
13644	Dindevan 25mg Tablet (Goldshield Pharmaceuticals Ltd)
13663	Innohep 20000 iu/ml Injection (LEO Pharma)
13716	Heparin sodium 25,000iu/ml Injection
14099	Clexane forte 150mg/ml Injection (Aventis Pharma)
14110	Tinzaparin sodium 10,000units/0.5ml solution for injection pre-filled syringes
14138	Enoxaparin sodium 60mg/0.6ml solution for injection pre-filled syringes
14212	Tinzaparin sodium 3,500units/0.35ml solution for injection pre-filled syringes
14308	Tinzaparin sodium 18,000units/0.9ml solution for injection pre-filled syringes
14341	Clexane Forte 150mg/1ml solution for injection pre-filled syringes (Sanofi)
14788	Innohep 10,000units/0.5ml solution for injection pre-filled syringes (LEO Pharma)
14794	Monoparin 1000iu/ml Injection (C P Pharmaceuticals Ltd)
14851	Tinzaparin sodium 4,500units/0.45ml solution for injection pre-filled syringes
14891	Dalteparin sodium 18,000units/0.72ml solution for injection pre-filled syringes
15006	Sinthrome 4mg Tablet (Alliance Pharmaceuticals Ltd)
15293	Heparin sodium 5000iu/ml pre-filled Injection
15376	Acenocoumarol 4mg tablets
15709	Tinzaparin 3500 IU/0.3ml Sterile solution
16061	Innohep 3,500units/0.35ml solution for injection pre-filled syringes (LEO Pharma)
16476	Fragmin 18,000units/0.72ml solution for injection pre-filled syringes (Pfizer Ltd)
16530	Fragmin 12,500units/0.5ml solution for injection pre-filled syringes (Pfizer Ltd)
17004	Tinzaparin sodium 20,000units/2ml solution for injection vials
17007	Tinzaparin sodium 2,500units/0.25ml solution for injection pre-filled syringes
17049	Innohep 18,000units/0.9ml solution for injection pre-filled syringes (LEO Pharma)
17484	Innohep 10,000 IU/ml Injection (LEO Pharma)
17592	Innohep 4,500units/0.45ml solution for injection pre-filled syringes (LEO Pharma)
17664	Clexane 60mg/0.6ml solution for injection pre-filled syringes (Sanofi)
17791	Innohep 5000iu/5ml Sterile solution (LEO Pharma)

17965	Marevan 500microgram tablets (AMCo)
18209	Fragmin 7,500units/0.3ml solution for injection pre-filled syringes (Pfizer Ltd)
18732	Innohep 3500 iu/0.3ml Sterile solution (LEO Pharma)
19280	Innohep 14,000units/0.7ml solution for injection pre-filled syringes (LEO Pharma)
19337	Multiparin 125,000units/5ml solution for injection vials (Wockhardt UK Ltd)
19486	Dalteparin sodium 7,500units/0.3ml solution for injection pre-filled syringes
19989	Tinzaparin sodium 40,000units/2ml solution for injection vials
20010	Uniparin calcium 25,000iu/ml Subcutaneous injection (C P Pharmaceuticals Ltd)
20024	Uniparin forte 10,000 iu/0.4ml Subcutaneous injection (C P Pharmaceuticals Ltd)
20028	Multiparin 5,000units/5ml solution for injection vials (Wockhardt UK Ltd)
20029	Multiparin 25,000units/5ml solution for injection vials (Wockhardt UK Ltd)
20153	Enoxaparin sodium 150mg/1ml solution for injection pre-filled syringes
20154	Enoxaparin sodium 100mg/1ml solution for injection pre-filled syringes
20411	Alphaparin 3000iu/0.5ml Injection (Grifols UK Ltd)
20754	WARFARIN
21233	Innohep 20,000units/2ml solution for injection vials (LEO Pharma)
21316	Innohep 40,000units/2ml solution for injection vials (LEO Pharma)
21365	Uniparin 5000iu/0.2ml Injection (C P Pharmaceuticals Ltd)
21490	Monoparin 5000iu/ml Injection (C P Pharmaceuticals Ltd)
21518	Monoparin 25,000iu/ml Injection (C P Pharmaceuticals Ltd)
22428	Dalteparin sodium 100,000units/4ml solution for injection vials
23078	Warfarin 1mg Tablet (WB Pharmaceuticals Ltd)
23570	Fondaparinux sodium 7.5mg/0.6ml solution for injection pre-filled syringes
23573	Fondaparinux sodium 5mg/0.4ml solution for injection pre-filled syringes
23579	Fondaparinux sodium 2.5mg/0.5ml solution for injection pre-filled syringes
24896	Heparin low molecular weight 2500 iu/0.2ml Sterile solution
25155	Fragmin 100,000units/4ml solution for injection vials (Pfizer Ltd)
25195	Heparin sodium 25,000iu/ml pre-filled Injection
25287	Unihep leo 1000unit/ml Injection (LEO Pharma)
26146	Heparin low molecular weight 10,000 iu/ml Sterile solution
27035	Pump-hep 1000unit/ml Infusion (LEO Pharma)
27139	Pentosan polysulfate sodium 100mg capsules
27325	Innohep 2,500units/0.25ml solution for injection pre-filled syringes (LEO Pharma)
28506	Heparin low molecular weight 3500 iu/0.3ml Sterile solution
28593	Heparin sodium 1000iu/ml pre-filled Injection
29043	Arixtra 2.5mg/0.5ml solution for injection pre-filled syringes (Aspen Pharma Trading Ltd)
29207	Innohep 5000iu/0.5ml Sterile solution (LEO Pharma)
29317	Tinzaparin 5000 i.u./0.5ml Sterile solution
29318	Heparin low molecular weight 2500 iu/ml Sterile solution
30108	Heparin calcium 5000iu/0.2ml Injection
30202	Warfarin wbp 1mg Tablet (Boehringer Ingelheim Ltd)
30203	Warfarin wbp 3mg Tablet (Boehringer Ingelheim Ltd)
30396	Unihep leo 5000iu/ml Injection (LEO Pharma)
31148	Flolan 500microgram powder and solvent (pH10.5) for solution for infusion vials

	(GlaxoSmithKline UK Ltd)
31511	Warfarin 3mg Tablet (WB Pharmaceuticals Ltd)
31937	Warfarin 5mg tablets (Teva UK Ltd)
32511	Tinzaparin 5000 i.u./5ml Sterile solution
32645	Heparin sodium 25,000iu/ml Injection
33307	Heparin sodium 5,000units/1ml solution for injection ampoules
33558	Monoparin calcium 5,000units/0.2ml solution for injection ampoules (Wockhardt UK Ltd)
33711	Warfarin 5mg Tablet (WB Pharmaceuticals Ltd)
34019	Warfarin 1mg tablets (IVAX Pharmaceuticals UK Ltd)
34086	Warfarin 3mg Tablet (Celltech Pharma Europe Ltd)
34087	Warfarin 1mg Tablet (Celltech Pharma Europe Ltd)
34088	Warfarin 5mg Tablet (Celltech Pharma Europe Ltd)
34095	Warfarin wbp 5mg Tablet (Boehringer Ingelheim Ltd)
34299	Warfarin 1mg tablets (Teva UK Ltd)
34416	Warfarin 1mg tablets (Kent Pharmaceuticals Ltd)
34417	Warfarin 3mg tablets (Teva UK Ltd)
34418	Warfarin 5mg tablets (Mylan)
34517	Warfarin 1mg tablets (Mylan)
34526	Warfarin 3mg tablets (Mylan)
34576	Warfarin 1mg Tablet (Lagap)
34691	Warfarin 5mg Tablet (Regent Laboratories Ltd)
34758	Warfarin 3mg tablets (IVAX Pharmaceuticals UK Ltd)
34864	Warfarin 5mg tablets (IVAX Pharmaceuticals UK Ltd)
34918	Warfarin 5mg tablets (Actavis UK Ltd)
35033	Heparin sodium 5,000units/5ml solution for injection vials
35941	Heparin sodium 5,000units/5ml solution for injection ampoules
36099	Warfarin 1mg/5ml oral suspension
36142	Heparin sodium 25,000units/1ml solution for injection ampoules
36172	Clexane 300mg/3ml solution for injection multidose vials (Sanofi)
36196	Heparin sodium 1,000units/1ml solution for injection ampoules
36911	Fragmin 10,000units/1ml solution for injection ampoules (Pfizer Ltd)
36989	Fragmin 10,000units/1ml solution for injection pre-filled syringes (Pfizer Ltd)
37086	Enoxaparin sodium 300mg/3ml solution for injection vials
37131	Heparin sodium 25,000units/5ml solution for injection vials
37613	Heparin sodium 10,000units/10ml solution for injection ampoules
37616	Heparin sodium 10 unit/ml Solution
37678	Heparin sodium 5,000units/0.2ml solution for injection ampoules
37704	Minihep 25,000iu/ml Subcutaneous preparation (LEO Pharma)
38041	Warfarin sodium 5mg/ml oral suspension
38044	Warfarin 5mg/5ml oral solution
38327	Arixtra 7.5mg/0.6ml solution for injection pre-filled syringes (Aspen Pharma Trading Ltd)
38536	Fondaparinux sodium 1.5mg/0.3ml solution for injection pre-filled syringes
38839	Arixtra 5mg/0.4ml solution for injection pre-filled syringes (Aspen Pharma Trading

	Ltd)
39119	Rivaroxaban 10mg tablets
39444	Dabigatran etexilate 110mg capsules
39503	Dabigatran etexilate 75mg capsules
39639	Xarelto 10mg tablets (Bayer Plc)
39755	Pradaxa 110mg capsules (Boehringer Ingelheim Ltd)
39866	Warfarin 1mg tablets (Almus Pharmaceuticals Ltd)
40143	Warfarin 500microgram tablets (A A H Pharmaceuticals Ltd)
40715	Heparin 100iu/ml Oral solution (LEO Pharma)
42106	Unihep leo 25,000iu/ml Injection (LEO Pharma)
42474	Pradaxa 75mg capsules (Boehringer Ingelheim Ltd)
42853	Heparin calcium 25,000iu/ml Injection
43407	Warfarin 3mg tablets (A A H Pharmaceuticals Ltd)
43408	Warfarin 1mg tablets (A A H Pharmaceuticals Ltd)
43409	Warfarin 5mg tablets (A A H Pharmaceuticals Ltd)
43655	Warfarin sodium oral solution
44238	Heparin 50 iu/5ml Flush solution (Wockhardt UK Ltd)
44491	Heparin sodium 125,000units/5ml solution for injection vials
44866	Warfarin sodium 1mg/ml oral supension SF
45597	Lepirudin 50mg powder for solution for injection vials
45911	Arixtra 1.5mg/0.3ml solution for injection pre-filled syringes (Aspen Pharma Trading Ltd)
46632	Dabigatran etexilate 150mg capsules
46678	Pradaxa 150mg capsules (Boehringer Ingelheim Ltd)
46924	Phenindione 10mg tablets (AMCo)
47207	Rivaroxaban 20mg tablets
47353	Rivaroxaban 15mg tablets
47397	Heparin sodium 25,000units/5ml solution for injection ampoules
47566	Apixaban 2.5mg tablets
47925	Xarelto 20mg tablets (Bayer Plc)
47944	Warfarin 1mg tablets (Actavis UK Ltd)
48070	Warfarin sodium tablets
48134	Xarelto 15mg tablets (Bayer Plc)
48673	Dalteparin sodium 10,000units/1ml solution for injection ampoules
48869	Warfarin 1mg/ml oral suspension sugar free
48966	Rivaroxaban 15mg tablets
49578	Dalteparin sodium 10,000units/1ml solution for injection pre-filled syringes
50000	Warfarin 1mg/ml oral suspension sugar free (A A H Pharmaceuticals Ltd)
50391	Fragmin 18,000units/0.72ml solution for injection pre-filled syringes (Waymade Healthcare Plc)
50994	Heparin sodium 500units/500ml infusion bags
51006	Clexane 80mg/0.8ml solution for injection pre-filled syringes (DE Pharmaceuticals)
51350	Fragmin 15,000units/0.6ml solution for injection pre-filled syringes (Waymade Healthcare Plc)
51484	Warfarin 1mg tablets (Bristol Laboratories Ltd)

51496	Warfarin 1mg tablets (Phoenix Healthcare Distribution Ltd)
51509	Warfarin 1mg tablets (APC Pharmaceuticals & Chemicals (Europe) Ltd)
51642	Clexane 100mg/1ml solution for injection pre-filled syringes (Lexon (UK) Ltd)
52004	Fragmin 12,500units/0.5ml solution for injection pre-filled syringes (Waymade Healthcare Plc)
52841	Heparin calcium 5,000units/0.2ml solution for injection ampoules
53350	Heparin sodium 1,000units/500ml infusion bags
53740	Eliquis 2.5mg tablets (Bristol-Myers Squibb Pharmaceuticals Ltd)
53745	Warfarin 3mg tablets (Bristol Laboratories Ltd)
53752	Warfarin 1mg tablets (Alliance Healthcare (Distribution) Ltd)
54066	Apixaban 5mg tablets
54234	Heparin sodium 1,000units/500ml infusion Viaflex bags (Baxter Healthcare Ltd)
54451	Rivaroxaban 20mg tablets
54892	Warfarin 1mg/ml oral suspension sugar free (Alliance Healthcare (Distribution) Ltd)
54927	Heparin sodium 2,000units/1litre infusion bags
54946	Warfarin 3mg tablets (Actavis UK Ltd)
55096	Fragmin 5,000units/0.2ml solution for injection pre-filled syringes (Waymade Healthcare Plc)
55316	Warfarin 3mg/5ml oral suspension
55490	Heparin sodium 10,000 iu/ml Injection
55565	Clexane 100mg/1ml solution for injection pre-filled syringes (DE Pharmaceuticals)
55577	Sinthrome 1mg tablets (Lexon (UK) Ltd)
55604	Orgaran 750units/0.6ml solution for injection ampoules (Aspen Pharma Trading Ltd)
56166	Heparin sodium 100units/1ml solution for injection ampoules
56289	Xarelto 20mg tablets (Bayer Plc)
56314	Warfarin 3mg tablets (Kent Pharmaceuticals Ltd)
56315	Anticoagulant Citrate Dextrose Solution formula A infusion 500ml bags
56398	Fragmin 5,000units/0.2ml solution for injection pre-filled syringes (Mawdsley-Brooks & Company Ltd)
56640	Xarelto 15mg tablets (Bayer Plc)
57032	Warfarin 1mg/ml oral suspension sugar free (Rosemont Pharmaceuticals Ltd)
58519	Warfarin 1mg tablets (DE Pharmaceuticals)
58594	Eliquis 5mg tablets (Bristol-Myers Squibb Pharmaceuticals Ltd)
58787	Warfarin 5mg tablets (Alliance Healthcare (Distribution) Ltd)
58962	Warfarin 3mg tablets (DE Pharmaceuticals)
59400	Warfarin 500microgram tablets (Sigma Pharmaceuticals Plc)
59578	Warfarin 3mg tablets (Phoenix Healthcare Distribution Ltd)
59761	Heparin sodium 1,000units/1ml solution for injection ampoules (Wockhardt UK Ltd)
60041	Danaparoid sodium 750units/0.6ml solution for injection ampoules
60188	Heparin sodium 5,000units/1litre infusion bags
60589	Warfarin 500microgram tablets (Actavis UK Ltd)
60949	Warfarin 5mg/5ml oral suspension
61949	Fondaparinux sodium 10mg/0.8ml solution for injection pre-filled syringes
62150	Rivaroxaban 2.5mg tablets
62309	Warfarin 500microgram tablets (Kent Pharmaceuticals Ltd)

62310	Warfarin 500microgram tablets (AMCo)
62856	Tinzaparin sodium 12,000units/0.6ml solution for injection pre-filled syringes
62902	Tinzaparin sodium 16,000units/0.8ml solution for injection pre-filled syringes
62959	Heparin calcium 5,000units/0.2ml solution for injection ampoules (A A H Pharmaceuticals Ltd)
63071	Warfarin 4mg tablets
63101	Tinzaparin sodium 8,000units/0.4ml solution for injection pre-filled syringes
63146	Heparin sodium 20,000units/20ml solution for injection ampoules
63169	Innohep 12,000units/0.6ml solution for injection pre-filled syringes (LEO Pharma)
63297	Heparin sodium 5,000units/5ml solution for injection vials (LEO Pharma)
63440	Epoprostenol 500microgram powder and solvent (pH10.5) for solution for infusion vials
63571	Innohep 16,000units/0.8ml solution for injection pre-filled syringes (LEO Pharma)
64133	Heparin sodium 5,000units/0.2ml solution for injection ampoules (A A H Pharmaceuticals Ltd)
64315	Anticoagulant solution ACD-A 500ml bags (Haemonetics Ltd)
64500	Xarelto 2.5mg tablets (Bayer Plc)
64559	Heparin sodium 1,000units/1ml solution for injection ampoules (A A H Pharmaceuticals Ltd)
64581	Innohep 8,000units/0.4ml solution for injection pre-filled syringes (LEO Pharma)
64678	Edoxaban 60mg tablets
64969	Clexane 20mg/0.2ml solution for injection pre-filled syringes (Sigma Pharmaceuticals Plc)
64998	Epoprostenol 1.5mg powder and solvent (pH10.5) for solution for infusion vials
65247	Edoxaban 30mg tablets
65285	Warfarin 1mg tablets (Crescent Pharma Ltd)
65496	Warfarin 500microgram tablets (Phoenix Healthcare Distribution Ltd)
65538	Elmiron 100mg capsules (Imported (United States))
65746	Warfarin 500microgram tablets (DE Pharmaceuticals)
65850	Lixiana 60mg tablets (Daiichi Sankyo UK Ltd)
65876	Edoxaban 15mg tablets
66286	Warfarin 2.5mg/5ml oral solution
66529	Lixiana 30mg tablets (Daiichi Sankyo UK Ltd)
66570	Warfarin 1mg tablets (Waymade Healthcare Plc)
68591	Warfarin 500microgram tablets (Alliance Healthcare (Distribution) Ltd)
68667	Warfarin 5mg capsules
68795	Warfarin 1mg capsules
69128	Warfarin 500micrograms/5ml oral solution
69194	Heparin low molecular weight 5000iu/0.2ml Sterile solution
70831	Phenindione 50mg tablets (AMCo)
70866	Inhixa 40mg/0.4ml solution for injection pre-filled syringes (Techdow Pharma England Ltd)
71132	Clexane 20mg/0.2ml solution for injection pre-filled syringes (DE Pharmaceuticals)
71196	Warfarin 1.5mg/5ml oral solution
71274	Inhixa 60mg/0.6ml solution for injection pre-filled syringes (Techdow Pharma England Ltd)

71303	Rivaroxaban 15mg tablets and Rivaroxaban 20mg tablets
71386	Warfarin 1mg/5ml oral solution (Special Order)
