# Supplementary material for: Comprehensive ascertainment of bleeding in patients prescribed different combinations of dual antiplatelet therapy (DAPT) and triple therapy (TT) in the UK: study protocol for three population-based cohort studies emulating ‘target trials’ (the ADAPTT Study)
Source: BMJ Open. 2019 Jun 4;9(6):e029388. doi: 10.1136/bmjopen-2019-029388 (PMC6561407; doi:10.1136/bmjopen-2019-029388)
Supplement: Author's manuscript [file bmjopen-2019-029388.draft_revisions.pdf]

BMJ Open is committed to open peer review. As part of this commitment we make the peer review history of every article we publish publicly available.

When an article is published we post the peer reviewers' comments and the authors' responses online. We also post the versions of the paper that were used during peer review. These are the versions that the peer review comments apply to.

The versions of the paper that follow are the versions that were submitted during the peer review process. They are not the versions of record or the final published versions. They should not be cited or distributed as the published version of this manuscript.

BMJ Open is an open access journal and the full, final, typeset and author-corrected version of record of the manuscript is available on our site with no access controls, subscription charges or pay-per-view fees (http://bmjopen.bmj.com).

If you have any questions on BMJ Open's open peer review process please email info.bmjopen@bmj.com

BMJ Open

Comprehensive ascertainment of bleeding in patients prescribed different combinations of dual antiplatelet therapy (DAPT) and triple therapy (TT) in the UK: study protocol for three population-based cohort studies emulating 'target trials' (the ADAPTT Study)

Journal:	*BMJ Open*
Manuscript ID	bmjopen-2019-029388
Article Type:	Protocol
Date Submitted by the Author:	24-Jan-2019

Complete List of Authors:	Pufulete, Maria; University of Bristol,<br>Harris, Jessica; University of Bristol, Clinical Trials and Evaluation Unit, Bristol Medical School, Level 7, Bristol Royal Infirmary<br>Sterne, Jonathan; University of Bristol, Dept of Social Medicine<br>Johnson, Thomas; University Hospitals Bristol NHS Foundation Trust, Bristol heart Institute<br>Lasserson, Daniel; University of Birmingham, Institute of Applied Health Research; University of Oxford, Nuffield Department of Medicine<br>Mumford, Andrew; University Hospitals Bristol NHS Foundation Trust, Department of Haematology<br>Doble, Brett; University of Oxford, Nuffield Department of Population Health<br>Wordsworth, Sarah; University of Oxford, University of Oxford<br>Benedetto, Umberto; University of Bristol, Clinical Sciences<br>Rogers, Chris; university of bristol, Clinical Science South Bristol<br>Loke, Yoon; University of East Anglia, Norwich Medical School<br>Pithara, Christalla; University of Bristol, CLAHRC West, 9th Floor, Whitefriars BS1 2NT<br>Redwood, Sabi; Bristol, Population Health Sciences<br>Reeves, Barnaby; University of Bristol, Bristol Heart Institute

Keywords:	Dual antiplatelet therapy (DAPT), Acute coronary syndrome (ACS), Percutaneous coronary intervention (PCI), Coronary artery bypass grafting (CABG), Clinical Practice Research Datalink (CPRD), Hospital Episode Statistics (HES)

SCHOLARONE™
Manuscripts

Comprehensive ascertainment of bleeding in patients prescribed different combinations of dual antiplatelet therapy (DAPT) and triple therapy (TT) in the UK: study protocol for three population-based cohort studies emulating 'target trials' (the ADAPTT Study)

Maria Pufulete[1*], Jessica Harris[1], Jonathan A C Sterne[2], Thomas W. Johnson[3], Daniel Lasserson[4], Andrew Mumford[5], Brett Doble[6], Sarah Wordsworth[6], Umberto Benedetto[5], Chris A Rogers[1], Yoon Loke[7], Christalla Pithara[8], Sabi Redwood[8], Barnaby C Reeves[1]

[1] Clinical Trials and Evaluation Unit, Bristol Trials Centre, University of Bristol, Bristol, UK
[2] NIHR Biomedical Research Centre, Department of Population Health Sciences, University of Bristol, Bristol, UK
[3] Bristol Heart Institute, Bristol, UK
[4] Institute of Applied Health Research, University of Birmingham, Birmingham, UK
[5] Bristol Medical School, University of Bristol, Bristol, UK
[6] Health Economics Research Centre, Nuffield Department of Population Health, University of Oxford, Oxford UK
[7] Norwich Medical School, University of East Anglia, Norwich, UK
[8] National Institute for Health Research Collaboration for Leadership in Applied Health Research and Care West (NIHR CLAHRC West), Bristol, UK

[*] **Correspondence to**: Maria Pufulete, Clinical Trials and Evaluation Unit, University of Bristol, Level 7, Bristol Royal Infirmary, Queen's Building, Bristol, United Kingdom. Tel. +44 117 3423284. Fax. +44 117 3423288. Email: maria.pufulete@bristol.ac.uk

Key words: Dual antiplatelet therapy (DAPT); acute coronary syndrome (ACS); percutaneous coronary intervention (PCI), coronary artery bypass grafting (CABG); bleeding; Clinical Practice Research Datalink (CPRD); Hospital Episode Statistics (HES).

Abbreviations: ACS, acute coronary syndrome; AP, aspirin and prasugrel; AT, aspirin and ticagrelor; BARC, Bleeding Academic Research Consortium; CABG, coronary artery bypass grafting; CI, confidence interval; CIPS, Continuous inpatient stays; CPRD, Clinical Practice Research Datalink; DAPT, dual antiplatelet therapy; GP, General Practitioner; HES; Hospital Episode Statistics; HR, hazard ratios, ICD, International Classification of Diseases; MI, myocardial infarction; NICE, National Institute for Health and Care Excellence; NIHR, National Institute for Health Research; NHS, National Health Service; ONS, Office for

For peer review only - http://bmjopen.bmj.com/site/about/guidelines.xhtml

National Statistics; OPCS, Office of Population Censuses and Surveys; PCI, percutaneous coronary intervention; RCT, Randomized controlled trial; RR, relative risk; TT, triple therapy.

Word count: 3468 words

Number of tables: 2

Number of figures: 2

For peer review only

For peer review only - http://bmjopen.bmj.com/site/about/guidelines.xhtml

Abstract

INTRODUCTION: 'Real world' bleeding in patients exposed to different regimens of dual antiplatelet therapy (DAPT) and triple therapy (TT, DAPT plus an anticoagulant) have a clinical and economic impact but have not been previously quantified.

METHODS AND ANALYSIS: We will use linked Clinical Practice Research Datalink (CPRD) and Hospital Episode Statistics (HES) data to assemble populations eligible for three "target trials" in patient groups: percutaneous coronary intervention (PCI); coronary artery bypass grafting (CABG); conservatively managed (medication only) acute coronary syndrome (ACS). Patients ≥18 years old will be eligible if, in CPRD records, they have: ≥1 year of data before the index event; no prescription for DAPT or anticoagulants in the preceding three months; a prescription for aspirin or DAPT within two months after discharge from the index event. The primary outcome will be any bleeding event (CPRD or HES) up to 12 months after the index event. We will estimate adjusted hazard ratios for time to first bleeding event comparing: aspirin and clopidogrel (reference) vs. aspirin and prasugrel or aspirin and ticagrelor after PCI; and aspirin (reference) vs. aspirin and clopidogrel after CABG and ACS. We will describe rates of bleeding in patients prescribed triple therapy (DAPT plus an anticoagulant). Potential confounders will be identified systematically using literature review, semi-structured interviews with clinicians and a short survey of clinicians. We will conduct sensitivity analyses addressing the robustness of results to the study's main limitation – that we will not be able to identify the intervention group for patients whose bleeding event occurs before a DAPT prescription in CPRD.

ETHICS AND DISSEMINATION: This protocol was approved by the Independent Scientific Advisory Committee for the UK Medicines and Healthcare Products Regulatory Agency Database Research (protocol 16_126R). The findings will be presented in peer-reviewed journals, lay summaries, and briefing papers to commissioners/other stakeholders.

ISRCTN: 76607611

For peer review only

Strengths and limitations of this study

This study has four main strengths:

- We designed our study using the framework recommended by the Cochrane Bias and Non-Randomized Studies Methods Groups for establishing appropriate patient populations, interventions and follow-up to emulate three hypothetical RCTs (target trials).
- We will describe (using linked Hospital Episode Statistics, HES, and Clinical Practice Research Datalink, CPRD) the frequency of "real world" bleeding events in populations of patients who undergo coronary interventions (percutaneous coronary intervention, PCI, and coronary artery bypass grafting, CABG) or have acute coronary syndrome (ACS) and are managed conservatively (medication only) and have treatment initiated with different antiplatelet regimens or antiplatelet and anticoagulant (e.g. warfarin, dabigatran, rivaroxaban, apixaban) regimens.
- We will compare the frequency of bleeding events by DAPT regimen in the different populations: PCI (aspirin and clopidogrel versus aspirin and prasugrel or aspirin and ticagrelor); CABG and ACS (aspirin versus aspirin and clopidogrel).
- We will identify potential confounders systematically using literature review, semi-structured interviews with clinicians (cardiologists, cardiac surgeons and general practitioner) and a short survey with an additional group of clinicians.

This study has two main limitations:

- Because there are no medication data in HES, we cannot identify the DAPT regimen that patients were prescribed at the time of their index event (PCI, CABG or ACS). We will assume that patients' first DAPT prescription that appears in CPRD after their hospital admission is what they were prescribed at discharge.
- For the same reason, we will not know the intervention group of patients who had major bleeding before leaving hospital or before their first prescription in CPRD and either died, stopped or changed antiplatelet therapy. We will conduct sensitivity analyses to address the robustness of results to different assumptions about the unknown intervention group in patients who died or had a bleeding event before a DAPT prescription in CPRD.

For peer review only - http://bmjopen.bmj.com/site/about/guidelines.xhtml

Introduction

Dual antiplatelet therapy (DAPT), a combination of aspirin and either clopidogrel, prasugrel, or ticagrelor, is recommended for secondary prevention of ischaemic events (heart attack and stroke) in people with coronary artery disease. Guidelines recommend that patients are treated with DAPT for 6 to 12 months following myocardial infarction (MI) and coronary interventions (percutaneous coronary intervention, PCI, and coronary artery bypass grafting, CABG) [1-3] and support the use of the more potent antiplatelet inhibitors ticagrelor and prasugrel [2]. Antiplatelet agents reduce the risk of ischaemic events, by preventing the formation of clots in atherosclerotic coronary arteries and within stents (following PCI) or grafts (following CABG), but increase the risk of bleeding [4]. Randomized controlled trials (RCTs) have shown that adding clopidogrel to aspirin leads to 1% excess risk of major bleeding (requiring admission to hospital) compared with aspirin alone [5, 6]. Prasugrel and ticagrelor reduce the risk of ischemic events further but also further increase the risk of bleeding [7]. Some patients (e.g. those with existing atrial fibrillation or those who develop atrial fibrillation after PCI, CABG or ACS) are prescribed an anticoagulant (e.g. warfarin, dabigatran, rivaroxaban, apixaban) in addition to DAPT (triple therapy, TT), which further increases the risk of bleeding.

"Real world" bleeding events that do not require any intervention are likely to be much more frequent than those reported in RCTs, which excluded patients at high risk of bleeding and mainly reported only on major bleeding. Bleeding events that do not result in hospitalisation are largely managed in primary care and may have a significant clinical and economic impact [8]. Minor and nuisance bleeding (nose and gum bleeds, bruising and prolonged bleeding from cuts) may also reduce adherence to DAPT and therefore the benefit of DAPT in non-adherent patients [9] who are at increased risk of a secondary ischaemic coronary episode [10]. Only three studies have reported the incidence and consequences of nuisance bleeding after DAPT [11-13]; these suggest that nuisance bleeding is common (affecting 29-38% of patients) and impacts on adherence (11% of patients in one study discontinued clopidogrel [12]). A nested case control study using the Health Improvement Network (a UK primary care database) reported an increased risk of upper gastrointestinal bleeding with clopidogrel and aspirin compared with aspirin alone (relative risk [RR], 2.08; 95% confidence interval [CI], 1.34 to 3.21) [14].

For peer review only - http://bmjopen.bmj.com/site/about/guidelines.xhtml

The economic impact of bleeding events associated with DAPT is also poorly characterized, in particular for minor bleeding events and their impact on health-related quality of life [8].This is not surprising given that health economic analyses often lack detailed data on adverse effects of interventions, despite consensus that such effects should be considered [15, 16]. To ensure appropriate decisions are made about which DAPT regimens to use in clinical practice, the health and resource use consequences of minor and major bleeding events should be incorporated into assessments of cost-effectiveness. For DAPT, this entails accounting for uncertainty in the absolute risk of bleeding, the impact of different bleeding events on health-related quality of life and treatment adherence and subsequent risk of secondary ischaemic events, and the cost implications of managing these bleeding events.

We propose to use Hospital Episode Statistics (HES) and the Clinical Practice Research Datalink (CPRD) databases to estimate the incidence of all bleeding events occurring in patients prescribed different DAPT or TT regimens after undergoing coronary interventions (PCI and CABG) and in conservatively-managed ACS patients. Our study will also provide parameter estimates to update existing cost effectiveness models. We will use the framework recommended by the Cochrane Bias and Non-Randomized Studies Methods Groups for establishing appropriate patient populations, interventions and follow-up to emulate the following three hypothetical RCTs (hereafter referred to as the target trials, **Table 1**) [17]:

1. In patients who have undergone PCI, estimate the effect on bleeding events of assignment to aspirin and clopidogrel (reference) versus aspirin and prasugrel or aspirin and ticagrelor.
2. In patients who have undergone CABG, estimate the effect on bleeding events of assignment to aspirin (reference) versus aspirin and clopidogrel.
3. In patients who are conservatively-managed patients after presenting with ACS, estimate the effect on bleeding events of assignment to aspirin (reference) versus aspirin and clopidogrel.

For peer review only - http://bmjopen.bmj.com/site/about/guidelines.xhtml

Methods

Data sources

CPRD is a database of primary care electronic health record data (available online via CPRD GOLD) from participating general practices, covering 7% of the UK population [18]. Patients included in CPRD are largely representative of the UK population in terms of age, sex, ethnicity, and body mass index. HES covers all hospital admissions for all English patients whose treatment is funded by the UK National Health Service (NHS), whether treated by the NHS or by independent providers [19]. Seventy-five percent of English general practices included in CPRD are linked to HES data [18]. We obtained data from 1 April 2009 to 31 July 2017; this period covers the introduction of the newer antiplatelet agents prasugrel and ticagrelor. This study protocol has been approved by the Independent Scientific Advisory Committee (ISAC) of the CPRD (protocol number 16_126R).

Study populations

Eligibility and exclusion criteria for the three target trials (for patients undergoing PCI, patients undergoing CABG; or patients hospitalised and conservatively-managed for ACS) are listed in **Table 1**. We will identify eligible patients who are included in CPRD and eligible for linkage with HES and Office for National Statistics (ONS) mortality data (because they have a valid NHS number and are registered at a practice that was participating in the linkage programme). Patients are included if they had a PCI, CABG or ACS (index event) a record in HES during the study period (1 April 2010 – 31 July 2016), and have at least 1 year of linked CPRD-HES data before the date of their index event. They must also have been prescribed one of the treatment regimens being compared in the target trial corresponding to their index event. One year's data preceding eligibility for the target trial is adequate to apply most of the exclusion criteria and determine co-morbidities and medication history: such information would be collected at baseline in a randomized trial. The following Office of Population Censuses and Surveys (OPCS) procedure codes (PCI and CABG) and International Classification of Diseases (ICD-10) codes (ACS no procedure) will be used to identify patients: PCI, K49, K50 & K75; CABG, K40, K41, K42, K43, K44, K45 & K46; ACS without a procedure, I20.0, I21, I22, I24.9 (with no OPCS code for PCI or CABG in the same hospital admission). **Figure 1** shows full details of the inclusion and exclusion criteria.

For peer review only - http://bmjopen.bmj.com/site/about/guidelines.xhtml

Table 1. Summary of three target trials and how observational data will be used to emulate these

PICO component	Target trial	Issues in emulating the target trial using observational data
Eligibility criteria	**Target trial 1 (PCI)**<br>Consecutive patients (age ≥18 years) undergoing PCI (emergency or elective). Exclusions: DAPT or anticoagulant use in the previous 3 months; major bleed requiring hospitalization in previous 12 months; renal failure requiring dialysis; intolerance/allergy to aspirin, clopidogrel, prasugrel or ticagrelor.<br>**Target trial 2 (CABG)**<br>Consecutive patients (age ≥18 years) undergoing CABG (urgent and elective). Exclusions: DAPT or anticoagulant use in the previous 3 months; other concomitant cardiac surgery (e.g. valve surgery); major bleed requiring hospitalization in previous 12 months; renal failure requiring dialysis; intolerance/allergy to aspirin, clopidogrel, prasugrel or ticagrelor.<br>**Target trial 3 (conservatively-managed ACS)**<br>Consecutive patients (age ≥18 years) hospitalized for an acute coronary syndrome (ACS): myocardial infarction (MI) with or without ST-elevation or unstable angina. Exclusions: PCI or CABG performed at time of ACS diagnosis; major bleed requiring hospitalization in previous 12 months; renal failure requiring dialysis; intolerance/allergy to aspirin, clopidogrel, prasugrel or ticagrelor.	CPRD-HES linked dataset contains information that allows us to identify all eligible patients for the three target trials. The study period is April 2009-July 2017. All eligible patients will have sufficient data (1 year) preceding their index event to apply the exclusion criteria and characterize the population (e.g. co-morbidities) and sufficient follow-up data (1 year) to identify outcomes. It is not possible to capture intolerance/allergy to aspirin, clopidogrel, prasugrel or ticagrelor.
Interventions	**Target trial 1 (PCI)**<br>Clopidogrel (75mg daily) or prasugrel (5mg or 10mg daily) or ticagrelor (90mg twice daily). All patients will receive aspirin (at a dose of 75mg daily, in line with current guidelines).<br>**Target trial 2 (CABG)**	Relevant interventions can be identified as CPRD has information on all medications (including doses) prescribed in primary care.

For peer review only - http://bmjopen.bmj.com/site/about/guidelines.xhtml

	Clopidogrel (75mg) in addition to aspirin (at a dose of 75mg daily, in line with current guidelines) or aspirin only (any dose, reflecting variation in usual care).<br>**Target trial 3 (conservatively-managed ACS)**<br>As for target trial 2.	
Assignment to interventions	Participants are assigned to DAPT interventions in hospital.	Participants enter the study at index procedure date for PCI and CABG, and episode start date for ACS, and will be assigned to DAPT interventions using first prescription in CPRD (within 2 months of hospitalization) as a proxy for what they were prescribed in hospital (there are no medications data in HES). This assignment will exclude a proportion of eligible patients (those who died or experienced a major bleed that caused them to stop DAPT, or patients who have no prescription for DAPT within the 2 month window); we will identify and describe the characteristics of these excluded patients.<br>In sensitivity analyses, we will address the robustness of results to different assumptions about the intervention group in those patients where the DAPT medication is unknown or a major bleed occurs prior to the first DAPT medication, by using multiple imputation models for handling missing data. Prior known information regarding the likely prescription based on patient characteristics or general policies will be incorporated in these analyses.
Follow-up	Starts at assignment to intervention and ends at first bleed or 12 months from assignment (whichever comes first).	Starts at time of hospitalization for PCI, CABG or ACS and ends at first bleed or 12 months from hospitalization (whichever comes first).
Primary outcome	Any bleed within 12 months of the start of DAPT (DAPT is prescribed at hospitalization for PCI, CABG or ACS).	Any bleed within 12 months of hospitalization for PCI, CABG or ACS.

For peer review only - http://bmjopen.bmj.com/site/about/guidelines.xhtml

Analysis	Intention to treat	According to first prescription for DAPT in CPRD

For peer review only

Interventions

The interventions of interest for the three target trials are shown in **Table 1**. Guidelines recommend low dose aspirin (75 to 100mg/d) plus either clopidogrel (75mg/d), prasugrel (5mg/d or 10mg/d), or ticagrelor (90mg twice/d) for PCI and conservatively-managed ACS patients. For PCI patients, the interventions of interest are aspirin and clopidogrel, aspirin and prasugrel, and aspirin and ticagrelor. In conservatively-managed ACS patients, clopidogrel is the most commonly prescribed second antiplatelet agent (in addition to aspirin) and a large proportion of patients are prescribed aspirin only, therefore the interventions of interest are aspirin only (75 to 100mg/d) and aspirin and clopidogrel. There is variation in aspirin prescription for CABG patients; some surgeons choose 75mg/d, others 150mg/d, or 300mg/d. surgeons may also prescribe an additional antiplatelet agent, most commonly clopidogrel. Therefore, the comparisons of interest in CABG patients are aspirin only (any dose, reflecting variations in usual care in different hospitals) and aspirin and clopidogrel (doses as for PCI). We have specified these comparisons based on preliminary feasibility counts from CPRD, which indicate that few CABG and conservatively-managed ACS patients are prescribed aspirin and prasugrel or aspirin and ticagrelor.

In the target trials the interventions would be assigned during the hospital stay, as soon as patients are eligible for antiplatelet therapy. Our observational dataset does not have information on medication given to patients at discharge, because HES does not include medications data. Therefore, the first time at which we have information on the antiplatelet regimen to which patients were assigned in hospital is when they receive their first primary care prescription/s for aspirin or DAPT, recorded in CPRD. It is reasonable to use these as a proxy for the medications that patients started in hospital, because patients' general practitioners are unlikely to change the prescriptions that were started in hospital.

We will classify patients according to the first prescription recorded in CPRD during the first two months after hospitalization for PCI, CABG, or ACS. This two-month window is based on variability in the amount of DAPT medication provided to patients in hospital following their PCI, CABG or ACS and hence variability in the time when they first request a repeat prescription from their general practice. A preliminary investigation showed that more than 80% of eligible patients have a prescription for one or more antiplatelet agents during this time period. If a patient only has a prescription for aspirin during the 2-month window after

For peer review only - http://bmjopen.bmj.com/site/about/guidelines.xhtml

hospital discharge, they will be assigned to an aspirin only intervention. If patients also receives a prescription for clopidogrel, prasugrel or ticagrelor, they will be assigned to aspirin/clopidogrel, aspirin/prasugrel or aspirin/ticagrelor. If there is a prescription for more than one additional antiplatelet agent in the 2-month window, we will assign the patient to an intervention based on the agent prescribed first. For example, if a patient has an aspirin prescription and a prescription for clopidogrel before a prescription for ticagrelor, the patient will be assigned to the aspirin/clopidogrel intervention. Patients with no prescriptions in CPRD for aspirin, or aspirin and clopidogrel, prasugrel or ticagrelor within the 2-month window will be excluded from the main analysis. We will conduct sensitivity analyses of all eligible patients including those with unknown DAPT regimens or a major bleed prior to first DAPT prescription, by estimating assignment to DAPT interventions for those with no prescription data using multiple imputation based on a range of assumptions [20].

Outcomes

The primary outcome will be any bleeding event, classified as type 2-5 by the Bleeding Academic Research Consortium (BARC) bleeding scale [21]. For each patient, we will identify all bleeding events in HES and CPRD during follow-up. We will not be able to identify BARC type 1 bleeding events (bleeding that is not actionable and does not cause the patient to seek treatment) as type 1 assumes there is no interaction with the health system or healthcare professionals therefore no bleed event will be recorded in HES or CPRD. We have specified a comprehensive list of bleeding codes in CPRD and HES (see **Appendix 1**). These will be categorised according to anatomical site for descriptive purposes. Secondary outcomes will be: any major bleeding event; any minor bleeding event; all-cause mortality; cardiovascular mortality; mortality from bleeding (these will be identified from linked ONS data); hospital admission for a bleeding event (inpatient HES); MI; stroke; additional coronary intervention.

Follow up

The start of follow up (the index event) will be the date of the index hospital procedure (PCI, CABG) or start date of the hospital episode that contains the ACS diagnosis (ACS). Patients will be followed up until 12 months after the index event, since DAPT is prescribed for 12 months in accordance to guidelines.

For peer review only - http://bmjopen.bmj.com/site/about/guidelines.xhtml

Confounding and co-interventions

Potential confounders (variables that predict both risk of bleeding and intervention group) will be specified *a priori* [22, 23]. We will identify confounders and co-interventions using literature review and clinician expertise as recommended by the Cochrane Bias and Non-Randomized Studies Methods Groups [17]. We will carry out a comprehensive and systematic literature search to identify all RCT and cohort studies of DAPT interventions, or cohort studies that identify predictors of bleeding. The literature searches for the review are included in **Appendix 2**. Abstracts will be screened by one researcher and full text papers will be obtained. Data on confounders and co-interventions will be extracted by two researchers independently using a data extraction form specifically designed for the study; variables extracted will include study characteristics, population characteristics (reported in the tables of baseline characteristics), factors adjusted for in the statistical analyses, and factors reported to predict risk of bleeding in our populations. We will not perform a risk of bias assessment because the aim of the review is only descriptive (i.e. the output will be lists of confounders and co-interventions) and there are no established criteria for assessing the validity with which primary researchers consider potential confounders and co-interventions; therefore it would be inappropriate to apply a risk-of-bias tool for studies estimating a treatment effect. We will use "saturation" as a criterion for discontinuing data collection, defined as review of 10 consecutive studies without identifying an additional confounder/co-intervention.

In parallel, we will conduct semi quantitative interviews with six clinicians in each of three groups: cardiologists; cardiac surgeons and general practitioners (GPs, to determine whether DAPT prescriptions are changed in primary care). The main aim of the clinician interviews is to understand DAPT prescribing practice in the UK and identify the factors (relating to patients, centres and prescribing practices of the individual doctors) that influence the decision about which antiplatelet regimen to prescribe. All factors that influence DAPT prescribing (confounders) identified from the literature review and clinician interviews will be combined in a short survey (SurveyMonkey®). The survey questionnaire will be administered online to all consultant members of the British Cardiovascular Society (cardiologists), the Society for Cardiothoracic Surgery (cardiac surgeons), and the Royal College of General Practitioners (GPs). The survey will be either emailed to all members individually (if the professional bodies agree) or advertised in weekly/monthly newsletters.

For peer review only - http://bmjopen.bmj.com/site/about/guidelines.xhtml

Confounders will be grouped in confounding domains [17]. We will attempt to identify each potential confounder (identified through the literature review, clinician interviews and survey) in the CPRD or HES dataset but acknowledge that there may be missing data across patients (and time) for some confounders.

Sample size

Estimated rates of bleeding with the different therapies are 5% for aspirin, 9% for aspirin/clopidogrel, and 12% for aspirin/prasugrel and aspirin/ticagrelor [5, 6, 24, 25]. Preliminary feasibility counts provided by CPRD suggest that there will at least the following numbers of patients eligible for each target trial:

- PCI: aspirin/clopidogrel (reference, 6738 patients) vs aspirin/prasugrel (842 patients) or aspirin/ticagrelor (770 patients)
- CABG: aspirin (reference, 2556 patients) vs aspirin/clopidogrel (595 patients)
- Conservatively-managed ACS: aspirin (reference, 8148 patients) vs aspirin/clopidogrel (3082 patients)

These estimates give expected event rates of at least 700 for PCI, 180 for CABG and 680 for ACS, assuming a ratio of 8:1 (aspirin/clopidogrel:aspirin/prasugrel or aspirin/clopidogrel:aspirin/ticagrelor) for PCI, 4:1 (aspirin:aspirin/clopidogrel) for CABG and 2.5:1 (aspirin:aspirin/clopidogrel) for ACS. The hazard ratios detectable with 90% and 80% power at the 5% statistical significance, assuming the group ratios given above are shown in **Table 2**. The correlation of the DAPT with other covariates adjusted for is unknown and we assessed the impact of a range of correlations (0, 0.3 and 0.5).

Table 2. Hazard ratios for a range of correlations for PCI, CABG and ACS

Ratio of presence: absence of covariate	Squared correlation with other covariates	Hazard ratio detectable 90% power	80% power
**PCI**			
8:1	0 (i.e. unadjusted)	1.48	1.41
	0.3	1.60	1.50
	0.5	1.74	1.62

For peer review only - http://bmjopen.bmj.com/site/about/guidelines.xhtml

**CABG**			
4:1	0 (i.e. unadjusted)	1.83	1.69
	0.3	2.06	1.87
	0.5	2.35	2.10
**Conservatively-managed ACS**			
2.5:1	0 (i.e. unadjusted)	1.32	1.27
	0.3	1.39	1.33
	0.5	1.48	1.40

Statistical analyses

We will describe temporal changes in DAPT prescribing and bleeding for PCI, CABG and ACS populations. We will use descriptive statistics to summarize the characteristics of the different intervention groups and standardised mean differences to compare them. We will estimate rates of bleeding (number of events/person time) with 95% confidence intervals (CIs) for each group. We will separate major and minor bleeding since adverse events of each type have different health and resource use consequences.

Analyses will estimate the effects of assigned intervention (analogous to an intention-to-treat analysis of a randomized trial) for the antiplatelet regimens corresponding to the first prescription of aspirin or DAPT in CPRD (see Interventions). We will use parametric survival models to estimate adjusted hazard ratios (HRs) with 95% CIs for the time to first bleeding event, comparing intervention groups for each target trial. Exploratory analyses, including assessment of proportional hazards assumptions, will be used to inform the choice of survival distribution (e.g. Weibull). Estimated time-dependent event probabilities will be used to update existing cost-effectiveness models [26]. The confounding factors to be included in the model (which will be identified as described previously and grouped into confounding domains from our dataset), the modelling strategy and the approach to handling correlated covariates will be documented in a data analysis plan. Participants free from a

For peer review only - http://bmjopen.bmj.com/site/about/guidelines.xhtml

bleeding event will be censored at 12 months after the index event. For secondary endpoints we will use survival models to estimate adjusted hazard ratios (HRs) with 95% CIs for time to first event. For mortality outcomes, we will take account of the competing risks of death due to other causes.

We will perform three sets of sensitivity analyses:

1. We will address the unknown intervention group of eligible patients who have no prescription data and therefore cannot be assigned to an intervention (i.e. those that died before receiving their first prescription, had a major bleed which caused them to stop DAPT, or have no aspirin/DAPT prescription recorded in CPRD within the 2 month window). These analyses will be undertaken using multiple imputation methods to deal with missing information on DAPT medication and will take patient characteristics, procedure/diagnosis and general medication policies into account, using a range of assumptions.
2. We will address the possibility that some minor bleeding events are not documented in CPRD but nevertheless prompt medication changes. We anticipate that most bleeding events will occur soon after the index event and before any medication change. Medication changes will be described relative to bleeding events observed (e.g. before event, after event, no bleeding event observed). If a substantial proportion (>10%) of people change medication before their first bleeding event, we will perform a sensitivity analysis excluding these patients.
3. We will assess the sensitivity of our findings when the analysis is restricted to a sub-population of patients at low risk of bleeding (i.e. excluding people at high risk for bleeding) who, we hypothesise, have the lowest risk of residual confounding.

We will attempt to identify an instrumental variable (e.g. clinician/GP practice prescribing practice) to control for confounding by indication. If we are successful we will repeat the above analyses for the PCI, CABG and ACS populations.

We will explore the consistency of treatment effect estimates in the following subgroups: ACS vs non-ACS (PCI and CABG populations); diabetic vs non-diabetic; chronic kidney disease vs. non-chronic kidney disease; concurrent prescription for proton pump inhibitors vs. no prescription for proton pump inhibitors. All subgroups will be defined in the data analysis

For peer review only - http://bmjopen.bmj.com/site/about/guidelines.xhtml

plan and chosen based on the characteristics of the analysis populations before carrying out any analyses by intervention group. We will report p values from tests of interaction.

Triple therapy (TT)

We will establish additional groups of patients (PCI or CABG or conservatively-managed ACS) based on triple therapy (TT) prescription. The main reason for prescribing an anticoagulant is to treat atrial fibrillation in a patient otherwise eligible for either of the three target trials. Atrial fibrillation can precede (long-term anticoagulation for pre-existing atrial fibrillation) or follow the index event (anticoagulation for new-onset atrial fibrillation). **Figure 2** shows how the patient groups receiving TT will be constructed.

We will identify patients on long-term anticoagulation by identifying prescriptions of oral anticoagulants (warfarin, dabigatran, rivaroxaban, apixaban) in the 3 months before their index event and a concomitant prescription of any anticoagulant (TT) with DAPT and in the first two months after the index admission. Patients who develop atrial fibrillation after the index event will be identified from any new anticoagulant prescription during follow-up. We have compiled a list of all drug codes in CPRD for antiplatelet agents (aspirin, clopidogrel, prasugrel and ticagrelor) and anticoagulants (warfarin, dabigatran, rivaroxaban, apixaban) (see **Appendix 3**).

In patients receiving various TT regimens (grouped by pre-index event anticoagulant prescription and post-index event anticoagulant prescription in PCI, CABG and ACS separately, see Figure 2), we will describe rates of bleeding (number of events/person time) with 95% CIs for each group. We have not specified comparison groups because we know there is large variation in prescribing of anticoagulants;TT is usually prescribed for a relatively short period of time (1-6 months, depending on a patient's individual risks of thrombosis and bleeding) after which an antiplatelet agent (usually aspirin) is removed and patients continue to receive an anticoagulant and single antiplatelet for the remainder of the 12 months after the index admission.

Ethics and dissemination

For peer review only - http://bmjopen.bmj.com/site/about/guidelines.xhtml

For peer review only

This study protocol has approval from the Independent Scientific Advisory Committee for the UK Medicines and Healthcare products Regulatory Agency Database Research (protocol 16_126R). The findings will be presented at national/international conferences, published in peer-reviewed academic journals and accessible formats in newsletters to patients (where available). The findings will also be reported as a briefing paper to commissioners (e.g. commissioning groups, National Institute for Health and Care Excellence, NICE) and to other health care stakeholders with an interest in the research through the Cardiac & Stroke Networks. We will also present and discuss our results at local CPRD working groups to disseminate out methods to other researchers using the CPRD database.

For peer review only

Author contributions

MP: designed and will conduct the study and wrote the manuscript.

JH: study statistician responsible for data linkage and analysis.

JACS: provided strategic direction with respect to study design and analysis.

TJ: conceived the research question and provided cardiology expertise.

DL: provided general practice expertise, clinical epidemiological expertise

AM: conceived the research question and provided haematology expertise.

BD: provided health economics expertise.

SW: provided health economics expertise.

UB: provided cardiac surgery expertise.

CAR: provided guidance with respect to the statistical analysis.

YL: provided pharmacoepidemiology expertise.

CP: responsible for conduct of the qualitative study with clinicians.

SR: will oversee conduct and reporting of the qualitative study with clinicians.

BCR: provided strategic direction with respect to study design and analysis.

All authors read and approved the final version of the manuscript.

Funding

This study is funded by the National Institute for Health Research (NIHR) Health Technology Assessment (HTA 14/192/89). The British Heart Foundation and NIHR Bristol Biomedical Research Unit for Cardiovascular Disease funded some staff time (MP, JH,

For peer review only - http://bmjopen.bmj.com/site/about/guidelines.xhtml

BCR). The views expressed are those of the authors and not necessarily those of the NHS, the NIHR or the Department of Health.

Competing interests

TJ has received speaker & consultancy fees from Astra Zeneca & Daichi-Sankyo

None of the other authors have competing interests to declare.

Acknowledgements

We would like to thank Taria Murray-Thomas, senior researcher at CRPD, who assisted with the data requests. This study was designed and delivered in collaboration with the Clinical Trials and Evaluation Unit (CTEU), a UKCRC registered clinical trials unit which, as part of the Bristol Trials Centre, is in receipt of National Institute for Health Research CTU support funding.

For peer review only

References

1. http://www.nice.org.uk/guidance/cg172/resources/guidance-mi-secondary-prevention-pdf NCG: 2013.
2. Valgimigli M, Bueno H, Byrne RA, Collet JP, Costa F, Jeppsson A, Juni P, Kastrati A, Kolh P, Mauri L *et al*: **2017 ESC focused update on dual antiplatelet therapy in coronary artery disease developed in collaboration with EACTS: The Task Force for dual antiplatelet therapy in coronary artery disease of the European Society of Cardiology (ESC) and of the European Association for Cardio-Thoracic Surgery (EACTS)**. *European heart journal* 2018, **39**(3):213-260.
3. Wijns W, Kolh P, Danchin N, Di Mario C, Falk V, Folliguet T, Garg S, Huber K, James S, Knuuti J *et al*: **Guidelines on myocardial revascularization**. *European heart journal* 2010, **31**(20):2501-2555.
4. Tada T, Natsuaki M, Morimoto T, Furukawa Y, Nakagawa Y, Byrne RA, Kastrati A, Kadota K, Iwabuchi M, Shizuta S *et al*: **Duration of dual antiplatelet therapy and long-term clinical outcome after coronary drug-eluting stent implantation: landmark analyses from the CREDO-Kyoto PCI/CABG Registry Cohort-2**. *Circulation Cardiovascular interventions* 2012, **5**(3):381-391.
5. Main C, Palmer S, Griffin S, Jones L, Orton V, Sculpher M, Henderson R, Sudlow C, Hawkins N, Riemsma R: **Clopidogrel used in combination with aspirin compared with aspirin alone in the treatment of non-ST-segment-elevation acute coronary syndromes: a systematic review and economic evaluation**. *Health technology assessment (Winchester, England)* 2004, **8**(40):iii-iv, xv-xvi, 1-141.
6. Terpening C: **An appraisal of dual antiplatelet therapy with clopidogrel and aspirin for prevention of cardiovascular events**. *Journal of the American Board of Family Medicine : JABFM* 2009, **22**(1):51-56.
7. Pilgrim T, Windecker S: **Antiplatelet therapy for secondary prevention of coronary artery disease**. *Heart (British Cardiac Society)* 2014, **100**(22):1750-1756.

For peer review only - http://bmjopen.bmj.com/site/about/guidelines.xhtml

8. Doble B, Pufulete M, Harris J, Johnson T, Lasserson D, Reeves BC, Wordsworth S: **Health-related quality of life impact of minor and major bleeding events during dual antiplatelet therapy: A systematic review and patient preference elicitation study.** *Health & Quality of Life Outcomes* 2018, **16**:191.
9. Czarny MJ, Nathan AS, Yeh RW, Mauri L: **Adherence to dual antiplatelet therapy after coronary stenting: a systematic review**. *Clinical cardiology* 2014, **37**(8):505-513.
10. Cohen M: **Expanding the recognition and assessment of bleeding events associated with antiplatelet therapy in primary care**. *Mayo Clinic proceedings* 2009, **84**(2):149-160.
11. Ben-Dor I, Torguson R, Scheinowitz M, Li Y, Delhaye C, Wakabayashi K, Maluenda G, Syed AI, Collins SD, Gonzalez MA *et al*: **Incidence, correlates, and clinical impact of nuisance bleeding after antiplatelet therapy for patients with drug-eluting stents**. *American heart journal* 2010, **159**(5):871-875.
12. Roy P, Bonello L, Torguson R, de Labriolle A, Lemesle G, Slottow TL, Steinberg DH, Kaneshige K, Xue Z, Satler LF *et al*: **Impact of "nuisance" bleeding on clopidogrel compliance in patients undergoing intracoronary drug-eluting stent implantation**. *The American journal of cardiology* 2008, **102**(12):1614-1617.
13. Amin AP, Bachuwar A, Reid KJ, Chhatriwalla AK, Salisbury AC, Yeh RW, Kosiborod M, Wang TY, Alexander KP, Gosch K *et al*: **Nuisance bleeding with prolonged dual antiplatelet therapy after acute myocardial infarction and its impact on health status**. *Journal of the American College of Cardiology* 2013, **61**(21):2130-2138.
14. Garcia Rodriguez LA, Lin KJ, Hernandez-Diaz S, Johansson S: **Risk of upper gastrointestinal bleeding with low-dose acetylsalicylic acid alone and in combination with clopidogrel and other medications**. *Circulation* 2011, **123**(10):1108-1115.
15. Craig D, McDaid C, Fonseca T, Stock C, Duffy S, Woolacott N: **Are adverse effects incorporated in economic models? An initial review of current practice**. *Health technology assessment (Winchester, England)* 2009, **13**(62):1-71, 97-181, iii.
16. Heather EM, Payne K, Harrison M, Symmons DP: **Including adverse drug events in economic evaluations of anti-tumour necrosis factor-alpha drugs for adult rheumatoid arthritis: a systematic review of economic decision analytic models**. *PharmacoEconomics* 2014, **32**(2):109-134.
17. Sterne JA, Hernan MA, Reeves BC, Savovic J, Berkman ND, Viswanathan M, Henry D, Altman DG, Ansari MT, Boutron I *et al*: **ROBINS-I: a tool for assessing risk of bias in non-randomised studies of interventions**. *BMJ (Clinical research ed)* 2016, **355**:i4919.
18. Herrett E, Gallagher AM, Bhaskaran K, Forbes H, Mathur R, van Staa T, Smeeth L: **Data Resource Profile: Clinical Practice Research Datalink (CPRD)**. *International journal of epidemiology* 2015, **44**(3):827-836.
19. http://content.digital.nhs.uk/hes.
20. Sterne JA, White IR, Carlin JB, Spratt M, Royston P, Kenward MG, Wood AM, Carpenter JR: **Multiple imputation for missing data in epidemiological and clinical research: potential and pitfalls**. *BMJ (Clinical research ed)* 2009, **338**:b2393.
21. Mehran R, Rao SV, Bhatt DL, Gibson CM, Caixeta A, Eikelboom J, Kaul S, Wiviott SD, Menon V, Nikolsky E *et al*: **Standardized bleeding definitions for cardiovascular clinical trials: a consensus report from the Bleeding Academic Research Consortium**. *Circulation* 2011, **123**(23):2736-2747.
22. Reeves BC, Deeks JJ, Higgins JPT, Wells GA: **Chapter 13: Including non-randomized studies. In: Higgins JPT, Green S (editors), Cochrane Handbook for Systematic Reviews of Interventions. Version 5.0.2 [updated September 2009]. The Cochrane Collaboration, 2009.**
23. Reeves BC, Higgins JP, Ramsay C, Shea B, Tugwell P, Wells GA: **An introduction to methodological issues when including non-randomised studies in systematic reviews on the effects of interventions**. *Research synthesis methods* 2013, **4**(1):1-11.

For peer review only - http://bmjopen.bmj.com/site/about/guidelines.xhtml

24. Wiviott SD, Braunwald E, McCabe CH, Montalescot G, Ruzyllo W, Gottlieb S, Neumann FJ, Ardissino D, De Servi S, Murphy SA *et al*: **Prasugrel versus clopidogrel in patients with acute coronary syndromes**. *The New England journal of medicine* 2007, **357**(20):2001-2015.
25. Wallentin L, Becker RC, Budaj A, Cannon CP, Emanuelsson H, Held C, Horrow J, Husted S, James S, Katus H *et al*: **Ticagrelor versus clopidogrel in patients with acute coronary syndromes**. *The New England journal of medicine* 2009, **361**(11):1045-1057.
26. Briggs A, Claxton K, Sculpher M: **Decision Modelling for Health Economic Evaluation. New York, Oxford University Press Inc.** . 2006.

For peer review only

Figure 1. Study diagram describing the construction of the PCI, CABG and ACS (conservatively managed) populations

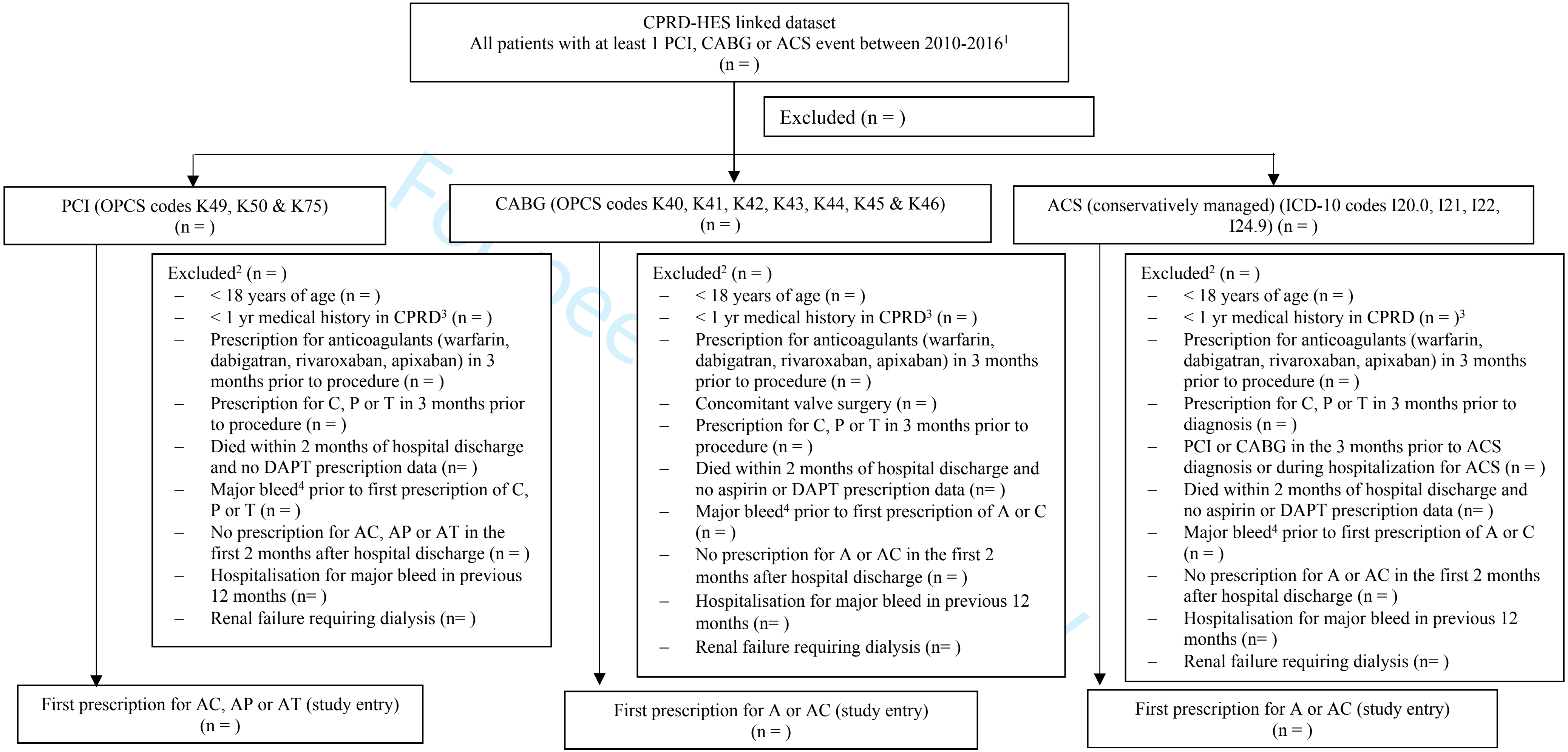

C: clopidogrel; P: prasugrel; T: ticagrelor; AC: aspirin + clopidogrel; AP: aspirin + prasugrel; AT: aspirin + ticagrelor
ACS: acute coronary syndrome; CABG: coronary artery bypass grafting; CPRD, Clinical Practice Research Datalink; HES; Hospital Episode Statistics; ICD International Classification of Diseases; OPCS, Office of Population Censuses and Surveys; PCI: percutaneous coronary intervention.

For peer review only - http://bmjopen.bmj.com/site/about/guidelines.xhtml

[1]Continuous inpatient stays (CIPS) that include both PCI and CABG are considered CABG; CIPS that include both ACS and PCI are considered PCI; CIPS that include both ACS and CABG are considered CABG. [2]Patients may be excluded for more than one reason. [3]At least one year of CPRD data available prior to event plus not "transferred out" before the event plus GP practice up to standard date precedes event date by at least 12 months. [4]Major bleed captured within hospital admission.

For peer review only

For peer review only - http://bmjopen.bmj.com/site/about/guidelines.xhtml

Figure 2. Study diagram describing the construction of the triple therapy (TT) populations

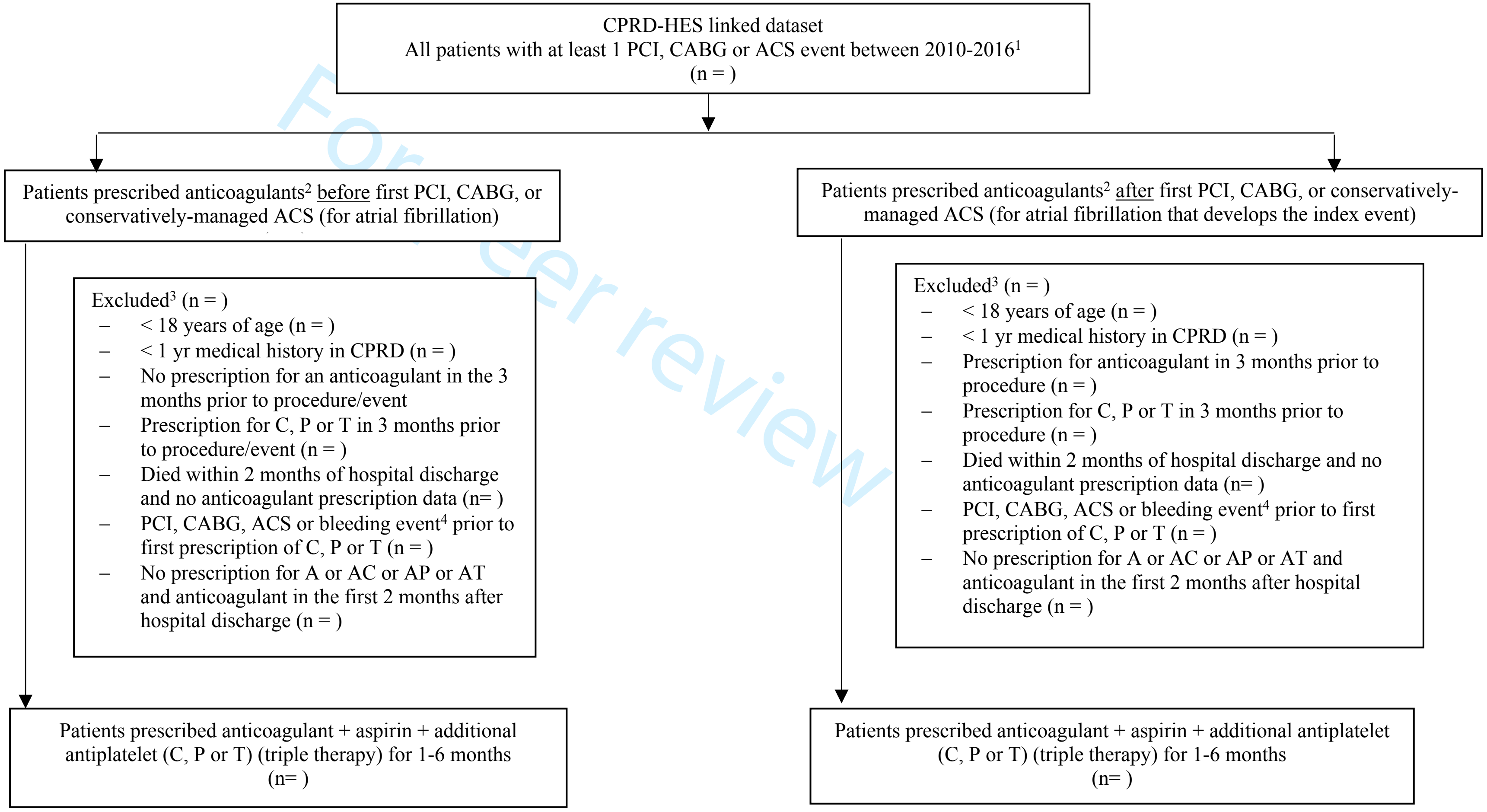

For peer review

C: clopidogrel; P: prasugrel; T: ticagrelor; AC: aspirin + clopidogrel; AP: aspirin + prasugrel; AT: aspirin + ticagrelor
ACS: acute coronary syndrome; CABG: coronary artery bypass grafting; CPRD, Clinical Practice Research Datalink; HES; Hospital Episode Statistics; ICD International Classification of Diseases; OPCS, Office of Population Censuses and Surveys; PCI: percutaneous coronary intervention.
[1]Continuous inpatient stays (CIPS) that include both PCI and CABG are considered CABG; CIPS that include both ACS and PCI are considered PCI; CIPS that include both ACS and CABG are considered CABG. [2]Warfarin, dabigatran, rivaroxaban, apixaban. [3]Patients may be excluded for more than one reason. [4]Bleeding event captured within hospital admission or GP appointment.

For peer review only

Appendix 1: List of Read codes and ICD-10 diagnosis codes for bleeding in Clinical Practice Research Datalink (CPRD) and Hospital Episode Statistics (HES)

Read Code	Read Term
J573011	Rectal bleeding
R047.00	[D]Epistaxis
158..12	Vaginal bleeding
K5A1.00	Postmenopausal bleeding
K59z.11	Break - through bleeding
1C6..11	Epistaxis symptom
J573.11	Bleeding PR
F4C7100	Subconjunctival haemorrhage
K597.00	Postcoital bleeding
16B3.00	Spontaneous bruising
1C6..00	Nose bleed symptom
K59y300	Intermenstrual bleeding
SE4..11	Leg bruise
K596.11	Intermenstrual bleeding - irregular
K59yx11	Dysfunctional uterine bleeding
196C.00	Painless rectal bleeding
SE4z.11	Haematoma NOS
J573000	Rectal haemorrhage
SE...11	Haematoma with intact skin
..00	Bruising symptom
SE3..11	Arm bruise
22E9.00	O/E-subconjunctival haemorrh'g
K59yx00	Dysfunctional uterine haemorrhage NOS
G60..00	Subarachnoid haemorrhage
F4C7200	Conjunctival haemorrhage NOS
G848000	Bleeding haemorrhoids NOS
R047.11	[D]Nosebleed
SE33011	Subungal haematoma
R027.11	[D]Spontaneous bruising
K596.00	Metrorrhagia
2I15.00	O/E - bruising
J68z.11	GIB - Gastrointestinal bleeding
196B.00	Painful rectal bleeding
J68..00	Gastrointestinal haemorrhage
SE23111	Perianal haematoma
1C62.00	Has nose bleeds - epistaxis
G8y0.00	Haemorrhage NOS
K5E..00	Other abnormal uterine and vaginal bleeding
SE10.00	Black eye NOS
K56y111	Bleeding - vaginal NOS

For peer review only

G61..00	Intracerebral haemorrhage
F42y500	Retinal haemorrhage NOS
F4K2800	Vitreous haemorrhage
J573.00	Haemorrhage of rectum and anus
G844.11	Perianal haematoma
2D85.00	O/E - blood in auditory canal
SE46.00	Traumatic haematoma
2BB8.00	O/E - vitreous haemorrhages
J68zz00	Gastrointestinal tract haemorrhage NOS
1C6Z.00	Nose bleed symptom NOS
SP21300	Primary post tonsillectomy haemorrhage
S622.00	Closed traumatic subdural haemorrhage
K575.00	Haematoma of vulva
K19y411	Urethral bleeding
K31y000	Breast haematoma due to nontraumatic cause
S62..13	Subdural haemorrhage following injury
7032000	Evacuation of extradural haematoma
SP21400	Secondary post tonsillectomy haemorrhage
K19y400	Bleeding from urethra
J68z000	Gastric haemorrhage NOS
J150000	Acute haemorrhagic gastritis
J510900	Bleeding diverticulosis
R09z000	[D]Umbilical bleeding
J68z100	Intestinal haemorrhage NOS
G60z.00	Subarachnoid haemorrhage NOS
K5E1.00	Abnormal uterine bleeding, unspecified
S62A.00	Traumatic extradural haematoma
J573100	Anal haemorrhage
F404500	Intra-ocular haemorrhage
SP21.12	Haemorrhage - postoperative
J120100	Acute duodenal ulcer with haemorrhage
S62..00	Cerebral haemorrhage following injury
SE22300	Haematoma of rectus sheath
J121111	Bleeding chronic duodenal ulcer
G62z.00	Intracranial haemorrhage NOS
G617.00	Intracerebral haemorrhage, intraventricular
J110111	Bleeding acute gastric ulcer
J68z.00	Gastrointestinal haemorrhage unspecified
K5C2.00	Haematocolpos
F503100	Haematoma of pinna
K286100	Scrotal haemorrhage
SP03216	Bleeding due to intrauterine contraceptive device
SE42011	Heel bruise
7M0G000	Aspiration of haematoma of organ NOC

For peer review only

K286v00	Male genital haematoma NOS
K566.00	Vaginal haematoma
SP21200	Post-operative haematoma formation
G602.00	Subarachnoid haemorrhage from middle cerebral artery
S62..11	Extradural haemorrhage following injury
F42y400	Subretinal haemorrhage
7004300	Evacuation of intracerebral haematoma NEC
G77z000	Capillary haemorrhage
SE06.00	Bruise of mandibular joint area
K544.00	Haematometra
S760100	Kidney haematoma without mention of open wound into cavity
K138300	Intrarenal haematoma
K59yy00	Functional uterine haemorrhage NOS
S760111	Renal haematoma without mention of open wound into cavity
7M0U400	Reexploration of organ & surgical arrest postop bleeding NOC
J111100	Chronic gastric ulcer with haemorrhage
7004200	Evacuation of haematoma from cerebellum
SP03217	Contraception IUCD causing bleeding
K275100	Corpus cavernosum haematoma
K286400	Testicular haemorrhage
ZA13700	Drainage of subungual haematoma with hot wire
S628.00	Traumatic subdural haemorrhage
7008200	Aspiration of haematoma of brain tissue
SE11.12	Bruise of periocular tissue
7H02200	Reopen chest reexplore intraabdom op site surg arr PO bleed
S624.00	Closed traumatic extradural haemorrhage
F4G3200	Exophthalmos due to orbital haemorrhage
S740100	Liver haematoma and contusion without open wound into cavity
S620.00	Closed traumatic subarachnoid haemorrhage
TA0..11	Accidental haemorrhage during medical care
K221100	Prostatic haemorrhage
SP21000	Intra-operative haemorrhage
S626.00	Epidural haemorrhage
7404z00	Surgical arrest of bleeding from internal nose NOS
C063000	Thyroid haemorrhage
ZA13800	Drainage of subungual haematoma with drill
Kyu9D00	[X]Other specified abnormal uterine and vaginal bleeding
H5y0000	Tracheostomy haemorrhage
7E0F500	Uterus operation haemostasis
7H22600	Reopen abdo reexplore intraabd op site surg arr postop bleed
F436z00	Choroidal haemorrhage or rupture NOS
Gyu6200	[X]Other intracerebral haemorrhage
F501G00	Haemorrhagic otitis externa
D305000	Haemorrhagic disorder due to antithrombinaemia

For peer review only

J11yy00	Unspec gastric ulcer; unspec haemorrhage and/or perforation
J13y100	Unspecified peptic ulcer with haemorrhage
D305.00	Haemorrhagic disorder due to circulating anticoagulants
SK02.11	Secondary and recurrent haemorrhage
7404y00	Surgical arrest of bleeding from internal nose OS
J111300	Chronic gastric ulcer with haemorrhage and perforation
K138100	Renal artery haemorrhage
C154200	Adrenal haemorrhage
F42y300	Deep retinal haemorrhage
J12yy00	Unspec duodenal ulcer; unspec haemorrhage and/or perforation
J130300	Acute peptic ulcer with haemorrhage and perforation
K167.00	Haemorrhage into bladder wall
7609y11	Tanner devascularisation for bleeding varices
2DE7.00	O/E - throat haemorrhage
J14y100	Unspecified gastrojejunal ulcer with haemorrhage
Gyu6100	[X]Other subarachnoid haemorrhage
F437200	Haemorrhagic choroidal detachment
7303200	Drainage haematoma ext ear control cavity c bolster suture
Ryu7300	[X]Haemorrhage, not elsewhere classified
J13y300	Unspecified peptic ulcer with haemorrhage and perforation
Gyu6F00	[X]Intracerebral haemorrhage in hemisphere, unspecified
G852000	Oesophageal varices with bleeding in diseases EC
SE45.11	Haematoma of leg
1584	Heavy episode of vaginal bleeding
7017000	Evacuation of subdural haematoma
SP21.11	Haematoma - postoperative
J573012	PRB - Rectal bleeding
SE30011	Shoulder bruise
2D25.00	O/E - epistaxis
SE1..11	Bruise of eye
F42y.11	Haemorrhage - retinal
K5Ez.00	Abnormal uterine and vaginal bleeding, unspecified
7736000	Evacuation of perianal haematoma Subdural haemorrhage - nontraumatic
G621.00 K286000	Scrotal haematoma due to nontraumatic cause
K56y100	Haemorrhage of vagina
K56y112	BPV - Vaginal bleeding
SP21.00	Peri-operative haemorrhage or haematoma
K286w00	Male genital haemorrhage NOS
G61z.00	Intracerebral haemorrhage NOS
SE0..11	Bruise of face
16B2.00	Bruises easily
G622.00	Subdural haematoma - nontraumatic
SP21100	Post-operative haemorrhage

For peer review only

K59y.11	Metropathia haemorrhagica
S629.00	Traumatic subdural haematoma
2556	O/E - bleeding gums
J68z200	Upper gastrointestinal haemorrhage
F586200	Otorrhagia
G61..12	Stroke due to intracerebral haemorrhage
G842000	Internal bleeding haemorrhoids
G530.00	Haemopericardium
SK02.12	Secondary and recurrent haemorrhage
K587.00	Contact bleeding of cervix
G850.00	Oesophageal varices with bleeding
G61..11	CVA - cerebrovascular acid due to intracerebral haemorrhage
1928	Bleeding gums
2BB5.00	O/E - retinal haemorrhages
K5E2.00	Abnormal vaginal bleeding, unspecified
SE0..12	Bruise of head
7M0G400	Evacuation of haematoma NEC
SE43.11	Toenail bruise
SE2..11	Bruise, trunk
16BZ.00	Bruising symptom NOS
K599.00	Mid-cycle bleeding
16B4.00	Post-traumatic bruising
G623.00	Subdural haemorrhage NOS
R063000	[D]Cough with haemorrhage
G613.00	Cerebellar haemorrhage
G845000	External bleeding haemorrhoids
J12y100	Unspecified duodenal ulcer with haemorrhage
J573z00	Haemorrhage of rectum and anus NOS
G718.00	Leaking abdominal aortic aneurysm
SE24211	Bruise of scrotum
J110100	Acute gastric ulcer with haemorrhage
2D66.00	O/E - blood from ear
7303000	Drainage of haematoma of external ear
25T0.00	Bleeding stoma
R063100	[D]Pulmonary haemorrhage NOS
F436000	Unspecified choroidal haemorrhage
J10y000	Haemorrhage of oesophagus
G360.00	Haemopericardium/current comp folow acute myocardial infarction
SK02.00	Secondary and recurrent haemorrhage
J121100	Chronic duodenal ulcer with haemorrhage
ZA13600	Drainage of subungual haematoma
K286300	Testicular haematoma due to nontraumatic cause
SE05.11	Bruise of ear
7D05200	Evacuation of haematoma of vulva

For peer review only

J56y000	Haemoperitoneum - nontraumatic
G614.00	Pontine haemorrhage
K5E0.00	Abnormal uterine bleeding unrelated to menstrual cycle
G611.00	Internal capsule haemorrhage
G60X.00	Subarachnoid haemorrhage from intracranial artery, unspecified
K13y800	Perirenal haematoma
7004100	Evacuation of haematoma from temporal lobe of brain
K16y200	Bladder haemorrhage
K221.00	Prostatic congestion or haemorrhage
G61X000	Left sided intracerebral haemorrhage, unspecified
66UI.00	Hormone replacement therapy bleed pattern - abnormal
G620.00	Extradural haemorrhage - nontraumatic
851..00	Haemorrhage control by packing
F4Ey000	Haemorrhage of eyelid
G61X100	Right sided intracerebral haemorrhage, unspecified
7D1C000	Evacuation of haematoma from vagina
SE11.11	Bruise of eyelids
R048. 00	[D]Throat haemorrhage
7404	Surgical arrest of bleeding from internal nose
S750100	Spleen haematoma without mention of open wound into cavity
F436.00	Choroidal haemorrhage and rupture
SE4z.12	Intramuscular haematoma NOS
F4K7.00	Retrobulbar haemorrhage
G603.00	Subarachnoid haemorrhage from anterior communicating artery
S62..12	Subarachnoid haemorrhage following injury
G610.00	Cortical haemorrhage
G62..00	Other and unspecified intracranial haemorrhage
662o.00	Haemorrhagic stroke monitoring
S630.12	Intracranial haematoma following injury
J130100	Acute peptic ulcer with haemorrhage
K55y300	Haemorrhage of cervix
G61X.00	Intracerebral haemorrhage in hemisphere, unspecified
K53y600	Haematosalpinx
J111111	Bleeding chronic gastric ulcer
G605.00	Subarachnoid haemorrhage from basilar artery
2D65.00	O/E - blood stained ear disch.
G612.00	Basal nucleus haemorrhage
F42y100	Superficial retinal haemorrhage
G604.00	Subarachnoid haemorrhage from posterior communicating artery
K59B.00	Postmenopausal postcoital bleeding
761D500	Endoscopic injection haemostasis of duodenal ulcer
S62z.00	Cerebral haemorrhage following injury NOS
K537.00	Haematoma of the broad ligament
7405300	Insertion of Brighton epistaxis balloon

For peer review only

F4H4100	Optic nerve sheath haemorrhage
S624.11	Epidural haematoma following injury
7619100	Gastrotomy and ligation of bleeding point of stomach
K59A.00	Premenopausal postcoital bleeding
S62..14	Traumatic cerebral haemorrhage
S627.00	Traumatic subarachnoid haemorrhage
7G2H400	Liposuction removal of haematoma
J131100	Chronic peptic ulcer with haemorrhage
FyuH400	[X]Vitreous haemorrhage in diseases classified elsewhere
J11y100	Unspecified gastric ulcer with haemorrhage
J120300	Acute duodenal ulcer with haemorrhage and perforation
S629000	Traumatic subdural haematoma without open intracranial wound
G618.00	Intracerebral haemorrhage, multiple localized
F424300	Retinal pigment epithelium haemorrhagic detachment
F436100	Expulsive choroidal haemorrhage
G616.00	External capsule haemorrhage
G615.00	Bulbar haemorrhage
K275200	Corpus cavernosum haemorrhage
761D600	Endoscopic injection haemostasis of gastric ulcer
J017200	Teeth staining due to pulpal bleeding
7L1L300	Haemostasis of unspecified organ
SE05.12	Bruise of auricle
S761100	Kidney haematoma with open wound into cavity
K221z00	Prostatic congestion or haemorrhage NOS
S625.00	Open traumatic extradural haemorrhage
S63..00	Other cerebral haemorrhage following injury
G606.00	Subarachnoid haemorrhage from vertebral artery
7B37400	Open haemostasis of prostate
S63z.00	Other cerebral haemorrhage following injury NOS
7J01300	Reopen cranium reexploration op site arrest post op bleeding
G601.00	Subarachnoid haemorrhage from carotid siphon and bifurcation
D305100	Haemorrhagic disorder due to hyperheparinaemia
J121300	Chronic duodenal ulcer with haemorrhage and perforation
S751100	Spleen haematoma with open wound into cavity
C12y100	Haemorrhage of parathyroid
J110300	Acute gastric ulcer with haemorrhage and perforation
J12y300	Unspecified duodenal ulcer with haemorrhage and perforation
S623.00	Open traumatic subdural haemorrhage
J140100	Acute gastrojejunal ulcer with haemorrhage
S621.00	Open traumatic subarachnoid haemorrhage

For peer review only

ICD Code	ICD Term	Type
I85.0	Oesophageal varices with bleeding	gastrointestinal
K25.0	Gastric ulcer, acute with haemorrhage	gastrointestinal
K25.2	Gastric ulcer, acute with both haemorrhage and perforation	gastrointestinal
K25.4	Gastric ulcer, chronic or unspecified with haemorrhage	gastrointestinal
K25.6	Chronic or unspecified with both haemorrhage and perforation	gastrointestinal
K26.0	Duodenal ulcer, acute with haemorrhage	gastrointestinal
K26.2	Duodenal ulcer, acute with both haemorrhage and perforation	gastrointestinal
K26.4	Duodenal ulcer, chronic or unspecified with haemorrhage	gastrointestinal
K26.6	Chronic or unspecified with both haemorrhage and perforation	gastrointestinal
K27.0	Peptic ulcer, acute with haemorrhage	gastrointestinal
K27.2	Peptic ulcer, acute with both haemorrhage and perforation	gastrointestinal
K27.4	Peptic ulcer, chronic or unspecified with haemorrhage	gastrointestinal
K27.6	Chronic or unspecified with both haemorrhage and perforation	gastrointestinal
K28.0	Gastrojejunal ulcer, acute with haemorrhage	gastrointestinal
K28.2	Acute with both haemorrhage and perforation	gastrointestinal
K28.4	Gastrojejunal ulcer, chronic or unspecified with haemorrhage	gastrointestinal
K28.6	Chronic or unspecified with both haemorrhage and perforation	gastrointestinal
K29.0	Acute haemorrhagic gastritis	gastrointestinal
K62.5	Haemorrhage of anus and rectum	gastrointestinal
K66.1	Haemoperitoneum	gastrointestinal
K92.0	Haematemesis	gastrointestinal
K92.1	Melaena	gastrointestinal
K92.2	Gastrointestinal haemorrhage, unspecified	gastrointestinal
I60	Subarachnoid haemorrhage	intracranial bleed
I60.0	Subarachnoid haemorrhage from carotid siphon and bifurcation	intracranial bleed
I60.1	Subarachnoid haemorrhage from middle cerebral artery	intracranial bleed
I60.2	Subarachnoid haemorrhage from anterior communicating artery	intracranial bleed
I60.3	Subarachnoid haemorrhage from posterior communicating artery	intracranial bleed
I60.4	Subarachnoid haemorrhage from basilar artery	intracranial bleed
I60.5	Subarachnoid haemorrhage from vertebral artery	intracranial bleed
I60.6	Subarachnoid haemorrhage from other intracranial arteries	intracranial bleed
I60.7	Subarachnoid haemorrhage from intracranial artery, unspec	intracranial bleed
I60.8	Other subarachnoid haemorrhage	intracranial bleed
I60.9	Subarachnoid haemorrhage, unspecified	intracranial bleed
I61	Intracerebral haemorrhage	intracranial bleed

For peer review only

I61.0	Intracerebral haemorrhage in hemisphere, subcortical	intracranial bleed
I61.1	Intracerebral haemorrhage in hemisphere, cortical	intracranial bleed
I61.2	Intracerebral haemorrhage in hemisphere, unspecified	intracranial bleed
I61.3	Intracerebral haemorrhage in brain stem	intracranial bleed
I61.4	Intracerebral haemorrhage in cerebellum	intracranial bleed
I61.5	Intracerebral haemorrhage, intraventricular	intracranial bleed
I61.6	Intracerebral haemorrhage, multiple localized	intracranial bleed
I61.8	Other intracerebral haemorrhage	intracranial bleed
I61.9	Intracerebral haemorrhage, unspecified	intracranial bleed
I62	Other nontraumatic intracranial haemorrhage	intracranial bleed
I62.0	Subdural haemorrhage (acute)(nontraumatic)	intracranial bleed
I62.1	Nontraumatic extradural haemorrhage	intracranial bleed
I62.9	Intracranial haemorrhage (nontraumatic), unspecified	intracranial bleed
I69.0	Sequelae of subarachnoid haemorrhage	intracranial bleed
I69.1	Sequelae of intracerebral haemorrhage	intracranial bleed
I69.2	Sequelae of other nontraumatic intracranial haemorrhage	intracranial bleed
S06.4	Epidural haemorrhage	intracranial bleed

For peer review only

Appendix 2: ADAPTT literature searches

**DATABASE**	**Set 1. POPULATION + INTERVENTION + STUDIES** *(RCTs/Cohort - **Dual/Triple antiplatelet therapy**)*	**Set 2. POPULATION + INTERVENTION + OUTCOME** *(bleeding)*	**Set 3. POPULATION + OUTCOME + RISK** *Risk of bleeding after a coronary intervention*
**CENTRAL** (database of controlled studies: RCTs, CCTs, ITS, CBA)	**775**	Included in Set 1 (CENTRAL database)	**720**
**MEDLINE**	**1822**	**558** (de-duplicated against Set 1 MEDLINE)	**5001**
**Embase**	**1156**	**520** (de-duplicated against Set 1 EMBASE)	**1582**
Total=	**3753**	**1078**	**7303**
After de-duplication=	**2544**	**849**	**6273**

**Set 3 (P-O-R)**	**Search-within-a-search-1**: (score or scores or model or models or tool or tools or algorithm* or prognosis or predict or prediction or cohort):ti,ab n=1843
	**Search-within-a-search-2:** (risk near3 (score* or factor or factors or model or models or prediction or stratification or category or bleed*)):ti,ab n=3300 (prior to de-duplication)

1. OVID MEDLINE

[Epub Ahead of Print, In-Process & Other Non-Indexed Citations, Ovid MEDLINE(R) Daily and Ovid MEDLINE(R) 1946 to Present]

1. Acute Coronary Syndrome/

2. (acute coronary adj3 syndrome*).ti,ab,kf.

3. ACS.ti,ab,kf.

4. heart attack*1.ti,ab,kf.

5. exp Myocardial Infarction/

6. myocardial infarct*.ti,ab,kf.

7. (MI or AMI).ti,ab,kf.

8. (stemi or non-stemi or nstemi).ti,ab,kf.

9. exp Angina, Unstable/

10. (angina adj3 unstable).ti,ab,kf.

11. exp Percutaneous Coronary Intervention/

12. (percutaneous coronary adj3 intervention).ti,ab,kf.

13. (PCI or PPCI or PCI-S).ti,ab,kf.

14. exp Angioplasty/

15. angioplasty.ti,ab,kf.

16. exp Stents/

17. stent*1.ti,ab,kf.

18. exp Coronary Artery Bypass/

19. CABG.ti,ab,kf.

20. coronary artery bypass.ti,ab,kf.

21. or/1-20 [Population]

22. (dual antiplatelet adj (therapy or treatment)).ti,ab,kf.

23. (DAPT or DAT).ti,ab,kf.

24. or/22-23

25. Aspirin/

26. (aspirin or acetylsalicylic acid or ASA).ti,ab,kf,rn,nm.

27. or/25-26

28. (clopidrogel or prasugrel or ticagrelor or plavix or efient or brilinta).ti,ab,kf,rn,nm,sh.

29. PURINERGIC P2Y RECEPTOR ANTAGONISTS/

30. (P2Y12 adj2 (antagonist* or inhibitor*)).ti,ab,kf,rn,nm.

31. or/28-30

32. 24 or (27 and 31)

33. exp Anticoagulants/

34. (anticoagul* or antithrombo* or anti-coagul* or anti-thrombo* or OAC* or DOAC* or NOAC*).ti,ab,kf,rn,nm.

35. (coumarin* or coumadin* or warfarin or marevan or dicoumarol or dicoumarin or dicumarin or dicumarol or acenocoumarol or phenindione or aldocumar or dabigatram or pradaxa or BIBR1048 or Apixaban or Eliquis or BMS-562247-01 or Edoxaban or Lixiana or

For peer review only

savaysa or DU-176b or betrixaban or PRT-054021 or PRT0504021 or rivaroxaban or xarelto or BAY-59739 or Erixaban or D0913).ti,ab,kf,rn,nm.

36. ((Vitamin K or Factor Xa or Factor 10a or Factor IIa) adj2 (antagonist* or inhibitor*)).ti,ab,kw,rn,nm.

37. or/33-36

38. (triple therapy or triple antiplatelet therapy or triple antithrombotic therapy or triple antithrombotic combination therapy).ti,ab,kf.

39. (TAPT or TOAT).ti,ab,kf.

40. ((24 or 31) and 37) or 38 or 39

41. 32 or 40 [Intervention]

42. (bleed*1 or bleeding).ti,ab,kf.

43. Hemorrhage/

44. (hemorrhag* or haemorrhag*).ti,ab,kf.

45. or/42-44 [Outcome]

46. risk/ or risk assessment/ or risk factors/

47. risk stratification.ti,ab,kf.

48. (risk adj3 model*).ti,ab,kf.

49. risk factor*.ti,ab,kf.

50. or/46-49 [Risk]

51. randomized controlled trial.pt.

52. controlled clinical trial.pt.

53. (RCT or randomi*).ti,ab,kf.

54. placebo.ab.

55. (random* adj (assign* or allocat* or divide* or division)).ti,ab,kf.

56. trial.ti,ab.

57. groups.ab.

58. or/51-57

59. cohort studies/ or follow-up studies/ or longitudinal studies/ or prospective studies/ or retrospective studies/

60. longitudinal.ab.

61. (prospective or retrospective).ab.

62. (CCT or (control* adj (trial*1 or study or studies))).ti,ab,kf.

For peer review only

63. (Follow up adj2 (study or studies)).ti,ab,kf.

64. follow up assessment.ti,ab,kf.

65. (compar* and group*).ab.

66. cohort.ti,ab,kf.

67. (register or registry).ti,ab,kf.

68. or/59-67

69. 58 or 68 **[Study Design Filter]**

70. 21 and 41 and 69

71. 21 and 41 and 45

72. 21 and 41 and 50

73. 21 and 41 and 45 and 50

74. 21 and 45 and 50

2. The Cochrane Library, Issue 7, 2016

ID	Search
#1	MeSH descriptor: [Acute Coronary Syndrome] explode all trees
#2	"acute coronary syndrome":ti,ab,kw (Word variations have been searched)
#3	ACS:ab (Word variations have been searched)
#4	heart attack*:ti,ab,kw (Word variations have been searched)
#5	MeSH descriptor: [Myocardial Infarction] explode all trees
#6	myocardial next infarct*:ti,ab,kw (Word variations have been searched)
#7	MI or AMI:ab (Word variations have been searched)
#8	(stemi or non-stemi or nstemi):ti,ab,kw (Word variations have been searched)
#9	MeSH descriptor: [Angina, Unstable] explode all trees
#10	(angina near unstable):ti,ab,kw (Word variations have been searched)
#11	MeSH descriptor: [Percutaneous Coronary Intervention] explode all trees
#12	(percutaneous next coronary) and intervention:ti,ab,kw (Word variations have been searched)
#13	PCI or PPCI or PCI-S:ab (Word variations have been searched)
#14	MeSH descriptor: [Angioplasty] explode all trees
#15	angioplasty:ti,ab,kw (Word variations have been searched)
#16	MeSH descriptor: [Stents] explode all trees

For peer review only

#17 stent or stents or stenting:ti,ab,kw (Word variations have been searched)

#18 MeSH descriptor: [Coronary Artery Bypass] explode all trees

#19 CABG:ab (Word variations have been searched)

#20 "coronary artery bypass":ti,ab,kw (Word variations have been searched)

#21 (#1 or #2 or #3 or #4 or #5 or #6 or #7 or #8 or #9 or #10 or #11 or #12 or #13 or #14 or #15 or #16 or #17 or #18 or #19 or #20)

#22 (dual next antiplatelet) and (therapy or treatment):ti,ab,kw (Word variations have been searched)

#23 (DAPT or DAT):ti,ab,kw (Word variations have been searched)

#24 #22 or #23

#25 MeSH descriptor: [Aspirin] explode all trees

#26 aspirin or "acetylsalicylic acid":ti,ab,kw (Word variations have been searched)

#27 ASA:ab (Word variations have been searched)

#28 #25 or #26 or #27

#29 (clopidrogel or prasugrel or ticagrelor or plavix or efient or brilinta):ti,ab,kw (Word variations have been searched)

#30 MeSH descriptor: [Purinergic P2Y Receptor Antagonists] explode all trees

#31 (P2Y12 near (antagonist* or inhibitor*)):ti,ab,kw (Word variations have been searched)

#32 #29 or #30 or #31

#33 #24 or (#28 and #32)

#34 MeSH descriptor: [Anticoagulants] explode all trees

#35 (anticoagul* or antithrombo* or anti-coagul* or anti-thrombo* or OAC* or DOAC* or NOAC*):ti,ab,kw (Word variations have been searched)

#36 (coumarin* or coumadin* or warfarin or marevan or dicoumarol or dicoumarin or dicumarin or dicumarol or acenocoumarol or phenindione or aldocumar):ti,ab,kw (Word variations have been searched)

#37 dabigatram or pradaxa or BIBR1048 or Apixaban or Eliquis or BMS-562247-01 or Edoxaban or Lixiana or savaysa or DU-176b or betrixaban or PRT-054021 or PRT0504021 or rivaroxaban or xarelto or BAY-59739 or Erixaban or D0913:ti,ab,kw (Word variations have been searched)

#38 "vitamin K" and (antagonist* or inhibitor*):ti,ab,kw (Word variations have been searched)

#39 ("vitamin K" or "factor Xa" or "factor 10a" or "factor IIa") and (antagonist* or inhibitor*):ti,ab,kw (Word variations have been searched)

#40 #34 or #35 or #36 or #37 or #38 or #39

#41 triple near therapy:ti,ab,kw (Word variations have been searched)

For peer review only

#42 TAPT or TOAT:ab (Word variations have been searched)

#43 ((#24 or #32) and #40) or #41 or #42

#44 #33 or #43

#45 MeSH descriptor: [Hemorrhage] explode all trees

#46 bleed*:ti,ab,kw (Word variations have been searched)

#47 hemorrhag* or haemorrhag*:ti,ab,kw (Word variations have been searched)

#48 #45 or #46 or #47

#49 MeSH descriptor: [Risk] explode all trees

#50 "risk stratification":ti,ab,kw (Word variations have been searched)

#51 risk near (factor* or model*):ti,ab,kw (Word variations have been searched)

#52 #49 or #50 or #51

#53 #21 and #44

#54 #21 and #48 and #52

#55 (#54 not #53)

3. OVID EMBASE (1974 to date)

1. exp acute coronary syndrome/
2. (acute coronary adj3 syndrome*).ti,ab,kw.
3. ACS.ti,ab,kw.
4. heart attack*1.ti,ab,kw.
5. exp heart infarction/
6. myocardial infarct*.ti,ab,kw.
7. (MI or AMI).ti,ab,kw.
8. (stemi or non-stemi or nstemi).ti,ab,kw.
9. exp unstable angina pectoris/
10. (angina adj3 unstable).ti,ab,kw.
11. exp percutaneous coronary intervention/
12. (percutaneous coronary adj3 intervention).ti,ab,kw.
13. (PCI or PPCI or PCI-S).ti,ab,kw.
14. exp angioplasty/
15. angioplasty.ti,ab,kw.
16. exp stent/
17. stent*1.ti,ab,kw.

For peer review only

18. coronary artery bypass graft/

19. CABG.ti,ab,kw.

20. coronary artery bypass.ti,ab,kw.

21. or/1-20

22. (dual antiplatelet adj (therapy or treatment)).ti,ab,kw.

23. (DAPT or DAT).ti,ab,kw.

24. or/22-23

25. acetylsalicylic acid/

26. (aspirin or acetylsalicylic acid or ASA).ti,ab,kw,rn,tn.

27. or/25-26

28. (clopidrogel or prasugrel or ticagrelor or plavix or efient or brilinta).ti,ab,kw,rn,tn,sh.

29. antithrombocytic agent/

30. (P2Y12 adj2 (antagonist* or inhibitor*)).ti,ab,kw,rn.

31. exp purinergic receptor blocking agent/

32. or/28-31

33. 24 or (27 and 32)

34. exp anticoagulant agent/

35. (anticoagul* or antithrombo* or anti-coagul* or anti-thrombo* or OAC* or DOAC* or NOAC*).ti,ab,kw.

36. (coumarin* or coumadin* or warfarin or marevan or dicoumarol or dicoumarin or dicumarin or dicumarol or acenocoumarol or phenindione or aldocumar).ti,ab,kw,rn,tn.

37. (dabigatram or pradaxa or BIBR1048 or Apixaban or Eliquis or BMS-562247-01 or Edoxaban or Lixiana or savaysa or DU-176b or betrixaban or PRT-054021 or PRT0504021 or rivaroxaban or xarelto or BAY-59739 or Erixaban or D0913).ti,ab,kw,rn,tn.

38. (vitamin K adj2 (antagonist$ or inhibitor$)).ti,ab,kw,rn.

39. (factor Xa adj2 (antagonist$ or inhibitor$)).ti,ab,kw,rn.

40. (factor 10a adj2 (antagonist$ or inhibitor$)).ti,ab,kw,rn.

41. (factor IIa adj2 (antagonist$ or inhibitor$)).ti,ab,kw,rn.

42. (adjunct* or combin* or concurrent or cotherap* or co-therap* or dual or plus or triple).ti,ab,kw.

43. drug combination/

44. or/34-43

45. (triple therapy or triple antiplatelet therapy or triple antithrombotic therapy or triple antithrombotic combination therapy).ti,ab,kw.

46. (TAPT or TOAT).ti,ab,kw.

For peer review only

47. ((24 or 32) and 44) or 45 or 46

48. 33 or 47

49. (bleed*1 or bleeding).ti,ab,kw.

50. exp Bleeding/

51. (hemorrhag* or haemorrhag*).ti,ab,kw.

52. or/49-51

53. risk assessment/ or risk factor/ or patient risk/ or risk/ or high risk patient/

54. risk stratification.ti,ab,kw.

55. (risk adj3 model*).ti,ab,kw.

56. risk factor*.ti,ab,kw.

57. or/53-56

58. Randomized Controlled Trial/

59. Randomization/

60. (random* adj (assign* or allocat* or divide* or division)).ti,ab,kw.

61. (RCT or randomi*).ti,ab,kw.

62. trial.ti,ab.

63. placebo.ti,ab,kw.

64. ((assign$ or match or matched or allocation) adj5 (alternate or group$1 or intervention$1 or patient$1 or subject$1 or participant$1)).ti,ab,kw.

65. double blind procedure/

66. ((double or single or doubly or singly) adj (blind or blinded or blindly)).ti.

67. or/58-66

68. Controlled Clinical Trial/

69. (CCT or (controlled adj7 (study or design or trial))).ti,ab,kw.

70. cohort analysis/

71. cohort.ti,ab,kw.

72. longitudinal.ab.

73. (prospective or retrospective).ab.

74. follow up assessment.ti,ab,kw.

75. clinical trial/ or multicenter study/ or phase 2 clinical trial/ or phase 3 clinical trial/ or phase 4 clinical trial/

76. clinical study/ or exp longitudinal study/ or major clinical study/ or prospective study/ or retrospective study/

77. (Follow up adj2 study).ti,ab,kw.

For peer review only

78. register.ti,ab,kw.

79. or/68-78

80. 67 or 79

81. Animal experiment/ not (human experiment/ or human/)

82. (rat or rats or mouse or mice or swine or porcine or murine or sheep or lambs or pigs or piglets or rabbit or rabbits or cat or cats or dog or dogs or cattle or bovine or monkey or monkeys or trout or marmoset$1).ti. and animal experiment/

83. or/81-82

84. 80 not 83

85. 21 and 48 and 84

86. limit 85 to exclude medline journals

87. **21 and 48 and 52**

88. limit 87 to exclude medline journals

89. **21 and 48 and 57**

90. limit 89 to exclude medline journals

91. **21 and 48 and 52 and 57**

92. 86 or 88 or 90 or 91

For peer review only

Appendix 3: List of product codes in Clinical Practice Research Datalink (CPRD). All for antiplatelet agents (aspirin, clopidogrel, prasugrel and ticagrelor) and anticoagulants (warfarin, dabigatran, rivaroxaban, apixaban)

Antiplatelet agents (aspirin, clopidogrel, prasugrel and ticagrelor)

Gemscript product code	Product name	Category
3	Aspirin 75mg dispersible tablets	Aspirin
16	Aspirin 75mg tablets	Aspirin
34	Aspirin 75mg gastro-resistant tablets	Aspirin
111	ASPIRIN 40 MG CAP	Aspirin
216	ASPIRIN 70 MG TAB	Aspirin
254	Aspirin 300mg tablets	Aspirin
377	Aspirin 300mg dispersible tablets	Aspirin
383	ASPIRIN 60 MG TAB	Aspirin
393	Disprin 300mg dispersible tablets (Reckitt Benckiser Healthcare (UK) Ltd)	Aspirin
395	Aspirin mixture	Aspirin
434	Aspirin 300mg gastro-resistant tablets	Aspirin
1137	Nu-seals aspirin ec 300mg Gastro-resistant tablet (Eli Lilly and Company Ltd)	Aspirin
1486	ASPIRIN 75 MG SUP	Aspirin
2105	Solprin 300mg Tablet (Reckitt Benckiser Healthcare (UK) Ltd)	Aspirin
2607	Paynocil Tablet (Beecham Research Laboratories)	Aspirin
2628	Nu-seals aspirin ec 75mg Gastro-resistant tablet (Eli Lilly and Company Ltd)	Aspirin
2754	ASPIRIN SOLUBLE 150 MG TAB	Aspirin
2924	ASPIRIN 150 MG TAB	Aspirin
4271	ASPIRIN SOLUBLE 200 MG TAB	Aspirin
4523	ASPIRIN 50 MG CAP	Aspirin
6006	Nu-Seals 75 gastro-resistant tablets (Alliance Pharmaceuticals Ltd)	Aspirin
6007	Nu-Seals 300 gastro-resistant tablets (Alliance Pharmaceuticals Ltd)	Aspirin
6696	Micropirin 75mg gastro-resistant tablets (Dexcel-Pharma Ltd)	Aspirin
7417	ASPIRIN 40 MG TAB	Aspirin
7462	ASPIRIN 325 MG CAP	Aspirin
7486	ASPIRIN 37.5 MG TAB	Aspirin
7516	Aspirin 300mg effervescent tablets sugar free	Aspirin
7665	ASPIRIN SR 300 MG TAB	Aspirin
7915	ASPIRIN SR 100 MG TAB	Aspirin
7944	ASPIRIN SOLUBLE 40 MG CAP	Aspirin
8185	Disprin CV 300mg modified-release tablets (Reckitt Benckiser Healthcare (UK) Ltd)	Aspirin
8186	Aspirin 300mg modified-release tablets	Aspirin

For peer review only

8424	ASPIRIN PAED 81 MG TAB	Aspirin
8645	Aspirin 300mg effervescent tablets	Aspirin
8733	JUNIOR ASPIRIN 37.5 MG TAB	Aspirin
8734	ASPIRIN disp 37.5 MG TAB	Aspirin
8843	ASPIRIN 325 MG TAB	Aspirin
9027	ASPIRIN disp 150 MG TAB	Aspirin
9144	Caprin 75mg gastro-resistant tablets (Wockhardt UK Ltd)	Aspirin
9301	Aspirin 100mg modified-release tablets	Aspirin
10305	Aspirin 162.5mg capsules	Aspirin
10310	Aspirin powder	Aspirin
11941	ASPIRIN SACHETS 30 MG	Aspirin
11977	Aspro clear maximum strength tablets	Aspirin
12102	ASPIRIN SOLUBLE 100 MG TAB	Aspirin
13882	Imazin XL tablets (Napp Pharmaceuticals Ltd)	Aspirin
15397	ASPIRIN SOLUBLE 50 MG TAB	Aspirin
15517	ASPIRIN 100 MG SUP	Aspirin
17704	Platet 100mg Effervescent tablet (Roche Products Ltd)	Aspirin
17920	Disprin cv 100mg Modified-release tablet (Reckitt Benckiser Healthcare (UK) Ltd)	Aspirin
18030	Imazin XL forte tablets (Napp Pharmaceuticals Ltd)	Aspirin
18217	Aspirin 300mg orodispersible tablets sugar free	Aspirin
18329	Enprin 75mg gastro-resistant tablets (Galpharm International Ltd)	Aspirin
19189	Micropirin 75mg Gastro-resistant tablet (Ratiopharm UK Ltd)	Aspirin
19577	NU-SEALS ASPIRIN	Aspirin
19674	ASPIRIN DISPERSIBLE	Aspirin
19797	NU-SEALS ASPIRIN	Aspirin
19813	ASPIRIN SOLUBLE	Aspirin
20206	ASPIRIN 50 MG SUP	Aspirin
20840	Acetylsalicylic acid mix	Aspirin
21380	Aspirin 75mg / Isosorbide mononitrate 60mg modified-release tablets	Aspirin
21382	Aspirin 150mg / Isosorbide mononitrate 60mg modified-release tablets	Aspirin
21921	Postmi ec 300mg Gastro-resistant tablet (Ashbourne Pharmaceuticals Ltd)	Aspirin
22107	ASPIRIN disp 200 MG TAB	Aspirin
22138	Aspirin 324mg modified-release tablets	Aspirin
22232	Disprin Direct 300mg orodispersible tablets (Reckitt Benckiser Healthcare (UK) Ltd)	Aspirin
22618	Solprin 75mg Tablet (Reckitt Benckiser Healthcare (UK) Ltd)	Aspirin
22864	ASPIRIN PAED MIX	Aspirin
23488	Claradin 300mg Tablet (Nicholas Laboratories Ltd)	Aspirin
23495	ASPIRIN	Aspirin
23593	PostMI 75 dispersible tablets (Ashbourne Pharmaceuticals Ltd)	Aspirin
23878	Nu-seals cardio ec 75mg Gastro-resistant tablet (Genus Pharmaceuticals Ltd)	Aspirin

23932	Aspro Clear 300mg effervescent tablets (Bayer Plc)	Aspirin
24025	Caprin 300mg gastro-resistant tablets (Pinewood Healthcare)	Aspirin
24960	Aspirin 300mg tablets (Vantage)	Aspirin
25335	PostMI 75 EC tablets (Ashbourne Pharmaceuticals Ltd)	Aspirin
25718	Angettes 75 tablets (Bristol-Myers Squibb Pharmaceuticals Ltd)	Aspirin
27467	ASPIRIN SOLUBLE 400 MG TAB	Aspirin
28707	ASPIRIN M/F 324 MG TAB	Aspirin
29515	ACETYLSALICYLIC ACID	Aspirin
29759	Aspro Tablet (Roche Consumer Health)	Aspirin
29848	Aspirin 300mg with Glycine 150mg chewable tablets	Aspirin
30920	Aspirin 300mg Dispersible tablet (M & A Pharmachem Ltd)	Aspirin
31210	Aspirin 300mg Tablet (Co-operative)	Aspirin
31211	Aspirin 75mg Dispersible tablet (A A H Pharmaceuticals Ltd)	Aspirin
31858	Caspac xl 162.5mg Capsule (Pharmacia Ltd)	Aspirin
31870	Aspirin 320mg tablets	Aspirin
31938	Aspirin 75mg gastro-resistant tablets (Sandoz Ltd)	Aspirin
31953	Aspirin 75mg dispersible tablets (IVAX Pharmaceuticals UK Ltd)	Aspirin
31954	Aspirin 75mg dispersible tablets (Teva UK Ltd)	Aspirin
31956	Aspirin 75mg gastro-resistant tablets (Kent Pharmaceuticals Ltd)	Aspirin
32036	Aspirin 75mg dispersible tablets (Actavis UK Ltd)	Aspirin
32210	Aspirin 300mg dispersible tablets (Actavis UK Ltd)	Aspirin
32992	Aspirin 75mg gastro-resistant tablets (Mylan)	Aspirin
33293	Aspirin 75mg gastro-resistant tablets (Sterwin Medicines)	Aspirin
33320	Aspirin 75mg Dispersible tablet (Sovereign Medical Ltd)	Aspirin
33656	Aspirin 75mg dispersible tablets (A A H Pharmaceuticals Ltd)	Aspirin
33662	Aspirin 300mg Dispersible tablet (A A H Pharmaceuticals Ltd)	Aspirin
33668	Aspirin 300mg Dispersible tablet (Rusco Ltd)	Aspirin
33676	Aspirin 75mg dispersible tablets (Kent Pharmaceuticals Ltd)	Aspirin
34309	Aspirin 300mg dispersible tablets (A A H Pharmaceuticals Ltd)	Aspirin
34385	Aspirin 75mg Soluble tablet (Co-operative)	Aspirin
34386	Aspirin 300mg tablets (Actavis UK Ltd)	Aspirin
34434	Aspirin 75mg dispersible tablets (Thornton & Ross Ltd)	Aspirin
34485	Aspirin 75mg gastro-resistant tablets (IVAX Pharmaceuticals UK Ltd)	Aspirin
34611	Aspirin 75mg gastro-resistant tablets (C P Pharmaceuticals Ltd)	Aspirin
34666	Aspirin ec 300mg Gastro-resistant tablet (A A H Pharmaceuticals Ltd)	Aspirin
34762	Aspirin 300mg Gastro-resistant tablet (Galen Ltd)	Aspirin
34796	Aspirin 75mg Gastro-resistant tablet (Galen Ltd)	Aspirin
34797	Aspirin 75mg gastro-resistant tablets (Actavis UK Ltd)	Aspirin
34942	Aspirin 75mg Dispersible tablet (Nucare Plc)	Aspirin
36543	Aspirin 100mg effervescent tablets	Aspirin
37541	Aspirin 227mg medicated chewing-gum	Aspirin
39738	Aspirin 162.5mg modified-release capsules	Aspirin
40144	Aspirin 300mg Dispersible tablet (Thornton & Ross Ltd)	Aspirin

For peer review only

40381	Aspirin 75mg Soluble tablet (C P Pharmaceuticals Ltd)	Aspirin
41512	Aspirin 75mg gastro-resistant tablets (Teva UK Ltd)	Aspirin
41569	Aspirin 300mg tablets (A A H Pharmaceuticals Ltd)	Aspirin
41594	Aspirin 300mg Dispersible tablet (Teva UK Ltd)	Aspirin
42061	ASPIRIN 65 MG SUP	Aspirin
43060	Aspirin 300mg Soluble tablet (Celltech Pharma Europe Ltd)	Aspirin
43434	Aspirin 300mg gastro-resistant tablets (A A H Pharmaceuticals Ltd)	Aspirin
43679	Flamasacard 162.5mg Modified-release capsule (Abbey Pharmaceuticals Ltd)	Aspirin
43709	Aspirin 75mg gastro-resistant tablets (Almus Pharmaceuticals Ltd)	Aspirin
43806	Aspirin 300mg gastro-resistant tablets (Sandoz Ltd)	Aspirin
44639	Aspirin 300mg Dispersible tablet (Nucare Plc)	Aspirin
45643	Aspirin 75mg Soluble tablet (Celltech Pharma Europe Ltd)	Aspirin
45840	Aspirin 300mg Dispersible tablet (Numark Management Ltd)	Aspirin
45851	Aspirin 300mg Soluble tablet (Ranbaxy (UK) Ltd)	Aspirin
47937	Aspirin 75mg dispersible tablets (Wockhardt UK Ltd)	Aspirin
47992	Aspirin 75mg gastro-resistant tablets (A A H Pharmaceuticals Ltd)	Aspirin
48000	Aspirin 300mg tablets (Sigma Pharmaceuticals Plc)	Aspirin
48021	Aspirin 75mg Tablet (Hillcross Pharmaceuticals Ltd)	Aspirin
48165	Aspirin 300mg tablets (Aspar Pharmaceuticals Ltd)	Aspirin
48974	Aspirin 75mg tablets (Phoenix Healthcare Distribution Ltd)	Aspirin
49060	Aspirin 75mg dispersible tablets (Alliance Healthcare (Distribution) Ltd)	Aspirin
49220	Aspirin 300mg tablets (Kent Pharmaceuticals Ltd)	Aspirin
49685	Aspirin 75mg dispersible tablets (Sigma Pharmaceuticals Plc)	Aspirin
50555	Aspirin 300mg dispersible tablets (DE Pharmaceuticals)	Aspirin
50926	Aspirin 75mg dispersible tablets (The Boots Company Plc)	Aspirin
50949	Aspirin 75mg tablets (A A H Pharmaceuticals Ltd)	Aspirin
51561	Aspirin 75mg gastro-resistant tablets (Zanza Laboratories Ltd)	Aspirin
52044	Aspirin 300mg caplets (The Boots Company Plc)	Aspirin
52280	Aspirin 300mg Tablet (Wockhardt UK Ltd)	Aspirin
52618	Aspirin 75mg dispersible tablets (Bristol Laboratories Ltd)	Aspirin
52905	Aspirin 300mg tablets (Lloyds Pharmacy Ltd)	Aspirin
53178	Aspirin 75mg gastro-resistant tablets (Wockhardt UK Ltd)	Aspirin
53622	Aspirin 300mg Tablet (M & A Pharmachem Ltd)	Aspirin
53711	Aspirin 300mg Tablet (Nucare Plc)	Aspirin
53791	Aspirin 150mg suppositories (Alliance Healthcare (Distribution) Ltd)	Aspirin
53804	Aspirin 300mg gastro-resistant tablets (Alliance Healthcare (Distribution) Ltd)	Aspirin
53816	Aspirin 300mg dispersible tablets (Alliance Healthcare (Distribution) Ltd)	Aspirin
54284	Aspirin 75mg dispersible tablets (Almus Pharmaceuticals Ltd)	Aspirin
54430	Aspirin 75mg tablets (Alliance Healthcare (Distribution) Ltd)	Aspirin
54526	Aspirin 300mg tablets (Alliance Healthcare (Distribution) Ltd)	Aspirin

For peer review only

54565	Aspirin 75mg dispersible tablets (Lloyds Pharmacy Ltd)	Aspirin
54734	Aspirin 300mg tablets (Wockhardt UK Ltd)	Aspirin
54997	Aspirin 75mg dispersible tablets (Dowelhurst Ltd)	Aspirin
55230	Aspirin 300mg dispersible tablets (Kent Pharmaceuticals Ltd)	Aspirin
55579	Aspirin 300mg tablets (Almus Pharmaceuticals Ltd)	Aspirin
56007	Aspirin 300mg dispersible tablets (Sigma Pharmaceuticals Plc)	Aspirin
56736	Aspirin 300mg tablets (Waymade Healthcare Plc)	Aspirin
56883	Aspirin 75mg tablets (Waymade Healthcare Plc)	Aspirin
56995	Aspirin 75mg dispersible tablets (Phoenix Healthcare Distribution Ltd)	Aspirin
56996	Aspirin 75mg dispersible tablets (Waymade Healthcare Plc)	Aspirin
57057	Aspirin 75mg dispersible tablets (Wockhardt UK Ltd)	Aspirin
58331	Aspirin 300mg gastro-resistant tablets (Mylan)	Aspirin
59021	Aspirin 75mg gastro-resistant tablets (Bristol Laboratories Ltd)	Aspirin
59244	Aspirin 100mg capsules	Aspirin
59253	Aspirin 75mg gastro-resistant tablets (Waymade Healthcare Plc)	Aspirin
59728	Aspirin 75mg tablets (Alissa Healthcare Research Ltd)	Aspirin
59791	Aspirin 75mg dispersible tablets (Aspar Pharmaceuticals Ltd)	Aspirin
60127	Aspirin 75mg tablets (DE Pharmaceuticals)	Aspirin
60278	Aspirin 300mg tablets (DE Pharmaceuticals)	Aspirin
60693	Aspirin 15mg/5ml oral solution	Aspirin
60694	Aspirin 25mg/5ml oral solution	Aspirin
60777	Aspirin 75mg gastro-resistant tablets (DE Pharmaceuticals)	Aspirin
62334	Aspirin 300mg caplets (Wockhardt UK Ltd)	Aspirin
62430	Aspirin 300mg suppositories (A A H Pharmaceuticals Ltd)	Aspirin
63603	Laboprin Tablet (Laboratories For Applied Biology Ltd)	Aspirin
64071	Aspirin powder (J M Loveridge Ltd)	Aspirin
65027	Bisoprolol 5mg / Aspirin 100mg capsules	Aspirin
66345	Aspirin 75mg dispersible tablets (DE Pharmaceuticals)	Aspirin
66546	Aspirin 75mg dispersible tablets (Numark Ltd)	Aspirin
66563	Aspirin 75mg gastro-resistant tablets (Phoenix Healthcare Distribution Ltd)	Aspirin
66861	Aspirin 75mg effervescent tablets	Aspirin
67124	Bisoprolol 10mg / Aspirin 75mg capsules	Aspirin
67160	Aspirin 300mg dispersible tablets (Lloyds Pharmacy Ltd)	Aspirin
67362	Aspirin 300mg suppositories (Alliance Healthcare (Distribution) Ltd)	Aspirin
67521	Aspirin 15mg/5ml oral suspension	Aspirin
67754	Aspirin 300mg dispersible tablets (Almus Pharmaceuticals Ltd)	Aspirin
67858	Aspirin 25mg capsules	Aspirin
68051	Aspirin 150mg suppositories (Colorama Pharmaceuticals Ltd)	Aspirin
68752	Aspirin 75mg tablets (Sigma Pharmaceuticals Plc)	Aspirin
70549	Danamep 75mg dispersible tablets (Ecogen Europe Ltd)	Aspirin
70841	Aspirin 300mg Dispersible tablet (Family Health)	Aspirin

For peer review only

71078	Aspirin 300mg dispersible tablets (Mawdsley-Brooks & Company Ltd)	Aspirin
71192	Aspirin 75mg tablets (Kent Pharmaceuticals Ltd)	Aspirin
489	Clopidogrel 75mg tablets	Clopidogrel
836	Plavix 75mg tablets (Sanofi)	Clopidogrel
17816	PLAVIX FC	Clopidogrel
17817	CLOPIDOGREL FC	Clopidogrel
38349	Clopidogrel 300mg tablets	Clopidogrel
38998	Plavix 300mg tablets (Sanofi)	Clopidogrel
40913	Grepid 75mg tablets (Kent Pharmaceuticals Ltd)	Clopidogrel
42750	Clopidogrel 75mg tablets (Actavis UK Ltd)	Clopidogrel
45905	Clopidogrel 1mg/ml oral suspension	Clopidogrel
46891	Clopidogrel 75mg/5ml oral suspension	Clopidogrel
52761	Clopidogrel 75mg tablets (Dr Reddy's Laboratories (UK) Ltd)	Clopidogrel
53751	Clopidogrel 75mg tablets (Phoenix Healthcare Distribution Ltd)	Clopidogrel
54700	Clopidogrel 75mg tablets (A A H Pharmaceuticals Ltd)	Clopidogrel
55161	Clopidogrel 75mg tablets (Wockhardt UK Ltd)	Clopidogrel
56807	Clopidogrel 75mg tablets (Teva UK Ltd)	Clopidogrel
57036	Clopidogrel 75mg tablets (Mylan)	Clopidogrel
58347	Clopidogrel 75mg tablets (DE Pharmaceuticals)	Clopidogrel
58448	Clopidogrel 75mg tablets (Aspire Pharma Ltd)	Clopidogrel
59904	Clopidogrel 75mg/5ml oral solution	Clopidogrel
62855	Clopidogrel 75mg tablets (Alliance Healthcare (Distribution) Ltd)	Clopidogrel
62978	Clopidogrel 75mg tablets (Sandoz Ltd)	Clopidogrel
63450	Clopidogrel 75mg tablets (Almus Pharmaceuticals Ltd)	Clopidogrel
65909	Clopidogrel 75mg tablets (Milpharm Ltd)	Clopidogrel
67037	Clopidogrel 75mg tablets (Zentiva)	Clopidogrel
39932	Prasugrel 10mg tablets	Prasugrel
40114	Prasugrel 5mg tablets	Prasugrel
40591	Efient 5mg tablets (Eli Lilly and Company Ltd)	Prasugrel
41229	Efient 10mg tablets (Eli Lilly and Company Ltd)	Prasugrel
45576	Ticagrelor 90mg tablets	Ticagrelor
47895	Brilique 90mg tablets (AstraZeneca UK Ltd)	Ticagrelor
66973	Ticagrelor 60mg tablets	Ticagrelor
68710	Brilique 60mg tablets (AstraZeneca UK Ltd)	Ticagrelor
70606	Ticagrelor 90mg orodispersible tablets sugar free	Ticagrelor

For peer review only

Anticoagulants (warfarin, dabigatran, rivaroxaban, apixaban)

Gemscript product code	Product name
45	Warfarin 1mg tablets
61	Warfarin 3mg tablets
833	Warfarin 3mg/5ml oral solution
1781	Warfarin 5mg tablets
2675	Fragmin 10,000units/4ml solution for injection ampoules (Pfizer Ltd)
2676	Fragmin 5,000units/0.2ml solution for injection pre-filled syringes (Pfizer Ltd)
2677	Clexane 100mg/ml Injection (Aventis Pharma)
3744	Heparin 10 iu/ml Flush solution
3895	Heparin sodium 1000iu/ml Injection
4446	Acenocoumarol 1mg tablets
4888	Heplok 10 iu/ml Oral solution (LEO Pharma)
4995	Enoxaparin 100mg/ml injection
5305	Sinthrome 1mg tablets (Merus Labs Luxco S.a R.L.)
5526	Fragmin 2,500units/0.2ml solution for injection pre-filled syringes (Pfizer Ltd)
5747	Fragmin 25,000iu/ml Solution for injection (Pfizer Ltd)
5998	Fragmin 10,000 iu/ml Solution for injection (Pfizer Ltd)
6262	Warfarin 500microgram tablets
6478	Enoxaparin sodium 20mg/0.2ml solution for injection pre-filled syringes
6695	Dalteparin sodium 2,500units/0.2ml solution for injection pre-filled syringes
6822	Elmiron 100mg capsules (Teva UK Ltd)
6860	Fragmin 15,000units/0.6ml solution for injection pre-filled syringes (Pfizer Ltd)
7154	Clexane Forte 120mg/0.8ml solution for injection pre-filled syringes (Sanofi)
7199	Enoxaparin sodium 40mg/0.4ml solution for injection pre-filled syringes
7307	Clexane 40mg/0.4ml solution for injection pre-filled syringes (Sanofi)
7371	Clexane 100mg/1ml solution for injection pre-filled syringes (Sanofi)
8466	Marevan 1mg tablets (AMCo)
8467	Marevan 3mg tablets (AMCo)
8664	Heparin sodium 5000iu/ml Injection
9140	Dalteparin sodium 10,000units/4ml solution for injection ampoules
9593	Dalteparin 25000iu/ml injection solution
9605	Dalteparin sodium 5,000units/0.2ml solution for injection pre-filled syringes
9610	Tinzaparin 20000 iu/ml Injection
9640	Tinzaparin 10000 IU/ml Injection
10002	Dalteparin 10000iu/1ml injection solution
10004	Clexane 80mg/0.8ml solution for injection pre-filled syringes (Sanofi)
10044	Dalteparin sodium 10,000units/0.4ml solution for injection pre-filled syringes
10072	Fragmin 10,000units/0.4ml solution for injection pre-filled syringes (Pfizer Ltd)
10170	Dalteparin sodium 15,000units/0.6ml solution for injection pre-filled syringes
10194	Dalteparin sodium 12,500units/0.5ml solution for injection pre-filled syringes

For peer review only

10240	Tinzaparin sodium 14,000units/0.7ml solution for injection pre-filled syringes
10532	Minihep calcium 5000iu/0.2ml Injection (LEO Pharma)
10533	Calciparine 25,000iu/ml Injection (Sanofi-Synthelabo Ltd)
10560	WARFARIN 10 MG TAB
11117	Hep-flush 100iu/ml Oral solution (LEO Pharma)
11372	Heparin 100iu/ml Flush solution
12681	Heparin calcium 25,000iu/ml Injection
12974	Clexane 150mg/ml Injection (Aventis Pharma)
13058	Enoxaparin 150mg/ml injection
13097	Clexane 20mg/0.2ml solution for injection pre-filled syringes (Sanofi)
13210	Enoxaparin sodium 80mg/0.8ml solution for injection pre-filled syringes
13270	Enoxaparin sodium 120mg/0.8ml solution for injection pre-filled syringes
13348	Marevan 5mg tablets (AMCo)
13501	Dindevan 50mg Tablet (Goldshield Pharmaceuticals Ltd)
13502	Dindevan 10mg Tablet (Goldshield Pharmaceuticals Ltd)
13503	Phenindione 50mg tablets
13504	Phenindione 25mg tablets
13505	Phenindione 10mg tablets
13568	Heparin sodium 25,000iu/ml Subcutaneous injection
13644	Dindevan 25mg Tablet (Goldshield Pharmaceuticals Ltd)
13663	Innohep 20000 iu/ml Injection (LEO Pharma)
13716	Heparin sodium 25,000iu/ml Injection
14099	Clexane forte 150mg/ml Injection (Aventis Pharma)
14110	Tinzaparin sodium 10,000units/0.5ml solution for injection pre-filled syringes
14138	Enoxaparin sodium 60mg/0.6ml solution for injection pre-filled syringes
14212	Tinzaparin sodium 3,500units/0.35ml solution for injection pre-filled syringes
14308	Tinzaparin sodium 18,000units/0.9ml solution for injection pre-filled syringes
14341	Clexane Forte 150mg/1ml solution for injection pre-filled syringes (Sanofi)
14788	Innohep 10,000units/0.5ml solution for injection pre-filled syringes (LEO Pharma)
14794	Monoparin 1000iu/ml Injection (C P Pharmaceuticals Ltd)
14851	Tinzaparin sodium 4,500units/0.45ml solution for injection pre-filled syringes
14891	Dalteparin sodium 18,000units/0.72ml solution for injection pre-filled syringes
15006	Sinthrome 4mg Tablet (Alliance Pharmaceuticals Ltd)
15293	Heparin sodium 5000iu/ml pre-filled Injection
15376	Acenocoumarol 4mg tablets
15709	Tinzaparin 3500 IU/0.3ml Sterile solution
16061	Innohep 3,500units/0.35ml solution for injection pre-filled syringes (LEO Pharma)
16476	Fragmin 18,000units/0.72ml solution for injection pre-filled syringes (Pfizer Ltd)
16530	Fragmin 12,500units/0.5ml solution for injection pre-filled syringes (Pfizer Ltd)
17004	Tinzaparin sodium 20,000units/2ml solution for injection vials
17007	Tinzaparin sodium 2,500units/0.25ml solution for injection pre-filled syringes
17049	Innohep 18,000units/0.9ml solution for injection pre-filled syringes (LEO Pharma)
17484	Innohep 10,000 IU/ml Injection (LEO Pharma)
17592	Innohep 4,500units/0.45ml solution for injection pre-filled syringes (LEO Pharma)
17664	Clexane 60mg/0.6ml solution for injection pre-filled syringes (Sanofi)

For peer review only

17791	Innohep 5000iu/5ml Sterile solution (LEO Pharma)
17965	Marevan 500microgram tablets (AMCo)
18209	Fragmin 7,500units/0.3ml solution for injection pre-filled syringes (Pfizer Ltd)
18732	Innohep 3500 iu/0.3ml Sterile solution (LEO Pharma)
19280	Innohep 14,000units/0.7ml solution for injection pre-filled syringes (LEO Pharma)
19337	Multiparin 125,000units/5ml solution for injection vials (Wockhardt UK Ltd)
19486	Dalteparin sodium 7,500units/0.3ml solution for injection pre-filled syringes
19989	Tinzaparin sodium 40,000units/2ml solution for injection vials
20010	Uniparin calcium 25,000iu/ml Subcutaneous injection (C P Pharmaceuticals Ltd)
20024	Uniparin forte 10,000 iu/0.4ml Subcutaneous injection (C P Pharmaceuticals Ltd)
20028	Multiparin 5,000units/5ml solution for injection vials (Wockhardt UK Ltd)
20029	Multiparin 25,000units/5ml solution for injection vials (Wockhardt UK Ltd)
20153	Enoxaparin sodium 150mg/1ml solution for injection pre-filled syringes
20154	Enoxaparin sodium 100mg/1ml solution for injection pre-filled syringes
20411	Alphaparin 3000iu/0.5ml Injection (Grifols UK Ltd)
20754	WARFARIN
21233	Innohep 20,000units/2ml solution for injection vials (LEO Pharma)
21316	Innohep 40,000units/2ml solution for injection vials (LEO Pharma)
21365	Uniparin 5000iu/0.2ml Injection (C P Pharmaceuticals Ltd)
21490	Monoparin 5000iu/ml Injection (C P Pharmaceuticals Ltd)
21518	Monoparin 25,000iu/ml Injection (C P Pharmaceuticals Ltd)
22428	Dalteparin sodium 100,000units/4ml solution for injection vials
23078	Warfarin 1mg Tablet (WB Pharmaceuticals Ltd)
23570	Fondaparinux sodium 7.5mg/0.6ml solution for injection pre-filled syringes
23573	Fondaparinux sodium 5mg/0.4ml solution for injection pre-filled syringes
23579	Fondaparinux sodium 2.5mg/0.5ml solution for injection pre-filled syringes
24896	Heparin low molecular weight 2500 iu/0.2ml Sterile solution
25155	Fragmin 100,000units/4ml solution for injection vials (Pfizer Ltd)
25195	Heparin sodium 25,000iu/ml pre-filled Injection
25287	Unihep leo 1000unit/ml Injection (LEO Pharma)
26146	Heparin low molecular weight 10,000 iu/ml Sterile solution
27035	Pump-hep 1000unit/ml Infusion (LEO Pharma)
27139	Pentosan polysulfate sodium 100mg capsules
27325	Innohep 2,500units/0.25ml solution for injection pre-filled syringes (LEO Pharma)
28506	Heparin low molecular weight 3500 iu/0.3ml Sterile solution
28593	Heparin sodium 1000iu/ml pre-filled Injection
29043	Arixtra 2.5mg/0.5ml solution for injection pre-filled syringes (Aspen Pharma Trading Ltd)
29207	Innohep 5000iu/0.5ml Sterile solution (LEO Pharma)
29317	Tinzaparin 5000 i.u./0.5ml Sterile solution
29318	Heparin low molecular weight 2500 iu/ml Sterile solution
30108	Heparin calcium 5000iu/0.2ml Injection
30202	Warfarin wbp 1mg Tablet (Boehringer Ingelheim Ltd)
30203	Warfarin wbp 3mg Tablet (Boehringer Ingelheim Ltd)
30396	Unihep leo 5000iu/ml Injection (LEO Pharma)

For peer review only

31148	Flolan 500microgram powder and solvent (pH10.5) for solution for infusion vials (GlaxoSmithKline UK Ltd)
31511	Warfarin 3mg Tablet (WB Pharmaceuticals Ltd)
31937	Warfarin 5mg tablets (Teva UK Ltd)
32511	Tinzaparin 5000 i.u./5ml Sterile solution
32645	Heparin sodium 25,000iu/ml Injection
33307	Heparin sodium 5,000units/1ml solution for injection ampoules
33558	Monoparin calcium 5,000units/0.2ml solution for injection ampoules (Wockhardt UK Ltd)
33711	Warfarin 5mg Tablet (WB Pharmaceuticals Ltd)
34019	Warfarin 1mg tablets (IVAX Pharmaceuticals UK Ltd)
34086	Warfarin 3mg Tablet (Celltech Pharma Europe Ltd)
34087	Warfarin 1mg Tablet (Celltech Pharma Europe Ltd)
34088	Warfarin 5mg Tablet (Celltech Pharma Europe Ltd)
34095	Warfarin wbp 5mg Tablet (Boehringer Ingelheim Ltd)
34299	Warfarin 1mg tablets (Teva UK Ltd)
34416	Warfarin 1mg tablets (Kent Pharmaceuticals Ltd)
34417	Warfarin 3mg tablets (Teva UK Ltd)
34418	Warfarin 5mg tablets (Mylan)
34517	Warfarin 1mg tablets (Mylan)
34526	Warfarin 3mg tablets (Mylan)
34576	Warfarin 1mg Tablet (Lagap)
34691	Warfarin 5mg Tablet (Regent Laboratories Ltd)
34758	Warfarin 3mg tablets (IVAX Pharmaceuticals UK Ltd)
34864	Warfarin 5mg tablets (IVAX Pharmaceuticals UK Ltd)
34918	Warfarin 5mg tablets (Actavis UK Ltd)
35033	Heparin sodium 5,000units/5ml solution for injection vials
35941	Heparin sodium 5,000units/5ml solution for injection ampoules
36099	Warfarin 1mg/5ml oral suspension
36142	Heparin sodium 25,000units/1ml solution for injection ampoules
36172	Clexane 300mg/3ml solution for injection multidose vials (Sanofi)
36196	Heparin sodium 1,000units/1ml solution for injection ampoules
36617	Angiox 250mg powder for concentrate for solution for infusion vials (The Medicines Company UK Ltd)
36911	Fragmin 10,000units/1ml solution for injection ampoules (Pfizer Ltd)
36989	Fragmin 10,000units/1ml solution for injection pre-filled syringes (Pfizer Ltd)
37086	Enoxaparin sodium 300mg/3ml solution for injection vials
37131	Heparin sodium 25,000units/5ml solution for injection vials
37613	Heparin sodium 10,000units/10ml solution for injection ampoules
37616	Heparin sodium 10 unit/ml Solution
37678	Heparin sodium 5,000units/0.2ml solution for injection ampoules
37704	Minihep 25,000iu/ml Subcutaneous preparation (LEO Pharma)
38041	Warfarin sodium 5mg/ml oral suspension
38044	Warfarin 5mg/5ml oral solution

For peer review only

38327	Arixtra 7.5mg/0.6ml solution for injection pre-filled syringes (Aspen Pharma Trading Ltd)
38536	Fondaparinux sodium 1.5mg/0.3ml solution for injection pre-filled syringes
38839	Arixtra 5mg/0.4ml solution for injection pre-filled syringes (Aspen Pharma Trading Ltd)
39119	Rivaroxaban 10mg tablets
39444	Dabigatran etexilate 110mg capsules
39503	Dabigatran etexilate 75mg capsules
39639	Xarelto 10mg tablets (Bayer Plc)
39755	Pradaxa 110mg capsules (Boehringer Ingelheim Ltd)
39866	Warfarin 1mg tablets (Almus Pharmaceuticals Ltd)
40143	Warfarin 500microgram tablets (A A H Pharmaceuticals Ltd)
40715	Heparin 100iu/ml Oral solution (LEO Pharma)
42106	Unihep leo 25,000iu/ml Injection (LEO Pharma)
42474	Pradaxa 75mg capsules (Boehringer Ingelheim Ltd)
42853	Heparin calcium 25,000iu/ml Injection
43407	Warfarin 3mg tablets (A A H Pharmaceuticals Ltd)
43408	Warfarin 1mg tablets (A A H Pharmaceuticals Ltd)
43409	Warfarin 5mg tablets (A A H Pharmaceuticals Ltd)
43655	Warfarin sodium oral solution
44238	Heparin 50 iu/5ml Flush solution (Wockhardt UK Ltd)
44491	Heparin sodium 125,000units/5ml solution for injection vials
44866	Warfarin sodium 1mg/ml oral supension SF
45597	Lepirudin 50mg powder for solution for injection vials
45911	Arixtra 1.5mg/0.3ml solution for injection pre-filled syringes (Aspen Pharma Trading Ltd)
46632	Dabigatran etexilate 150mg capsules
46678	Pradaxa 150mg capsules (Boehringer Ingelheim Ltd)
46924	Phenindione 10mg tablets (AMCo)
47207	Rivaroxaban 20mg tablets
47353	Rivaroxaban 15mg tablets
47397	Heparin sodium 25,000units/5ml solution for injection ampoules
47566	Apixaban 2.5mg tablets
47925	Xarelto 20mg tablets (Bayer Plc)
47944	Warfarin 1mg tablets (Actavis UK Ltd)
48070	Warfarin sodium tablets
48134	Xarelto 15mg tablets (Bayer Plc)
48673	Dalteparin sodium 10,000units/1ml solution for injection ampoules
48869	Warfarin 1mg/ml oral suspension sugar free
48966	Rivaroxaban 15mg tablets
49578	Dalteparin sodium 10,000units/1ml solution for injection pre-filled syringes
50000	Warfarin 1mg/ml oral suspension sugar free (A A H Pharmaceuticals Ltd)
50391	Fragmin 18,000units/0.72ml solution for injection pre-filled syringes (Waymade Healthcare Plc)
50994	Heparin sodium 500units/500ml infusion bags

For peer review only

51006	Clexane 80mg/0.8ml solution for injection pre-filled syringes (DE Pharmaceuticals)
51350	Fragmin 15,000units/0.6ml solution for injection pre-filled syringes (Waymade Healthcare Plc)
51484	Warfarin 1mg tablets (Bristol Laboratories Ltd)
51496	Warfarin 1mg tablets (Phoenix Healthcare Distribution Ltd)
51509	Warfarin 1mg tablets (APC Pharmaceuticals & Chemicals (Europe) Ltd)
51642	Clexane 100mg/1ml solution for injection pre-filled syringes (Lexon (UK) Ltd)
52004	Fragmin 12,500units/0.5ml solution for injection pre-filled syringes (Waymade Healthcare Plc)
52841	Heparin calcium 5,000units/0.2ml solution for injection ampoules
53350	Heparin sodium 1,000units/500ml infusion bags
53740	Eliquis 2.5mg tablets (Bristol-Myers Squibb Pharmaceuticals Ltd)
53745	Warfarin 3mg tablets (Bristol Laboratories Ltd)
53752	Warfarin 1mg tablets (Alliance Healthcare (Distribution) Ltd)
54066	Apixaban 5mg tablets
54234	Heparin sodium 1,000units/500ml infusion Viaflex bags (Baxter Healthcare Ltd)
54451	Rivaroxaban 20mg tablets
54892	Warfarin 1mg/ml oral suspension sugar free (Alliance Healthcare (Distribution) Ltd)
54927	Heparin sodium 2,000units/1litre infusion bags
54946	Warfarin 3mg tablets (Actavis UK Ltd)
55096	Fragmin 5,000units/0.2ml solution for injection pre-filled syringes (Waymade Healthcare Plc)
55316	Warfarin 3mg/5ml oral suspension
55490	Heparin sodium 10,000 iu/ml Injection
55565	Clexane 100mg/1ml solution for injection pre-filled syringes (DE Pharmaceuticals)
55577	Sinthrome 1mg tablets (Lexon (UK) Ltd)
55604	Orgaran 750units/0.6ml solution for injection ampoules (Aspen Pharma Trading Ltd)
56166	Heparin sodium 100units/1ml solution for injection ampoules
56289	Xarelto 20mg tablets (Bayer Plc)
56314	Warfarin 3mg tablets (Kent Pharmaceuticals Ltd)
56315	Anticoagulant Citrate Dextrose Solution formula A infusion 500ml bags
56398	Fragmin 5,000units/0.2ml solution for injection pre-filled syringes (Mawdsley-Brooks & Company Ltd)
56640	Xarelto 15mg tablets (Bayer Plc)
57032	Warfarin 1mg/ml oral suspension sugar free (Rosemont Pharmaceuticals Ltd)
58519	Warfarin 1mg tablets (DE Pharmaceuticals)
58594	Eliquis 5mg tablets (Bristol-Myers Squibb Pharmaceuticals Ltd)
58787	Warfarin 5mg tablets (Alliance Healthcare (Distribution) Ltd)
58962	Warfarin 3mg tablets (DE Pharmaceuticals)
59400	Warfarin 500microgram tablets (Sigma Pharmaceuticals Plc)
59578	Warfarin 3mg tablets (Phoenix Healthcare Distribution Ltd)
59761	Heparin sodium 1,000units/1ml solution for injection ampoules (Wockhardt UK Ltd)
60041	Danaparoid sodium 750units/0.6ml solution for injection ampoules
60188	Heparin sodium 5,000units/1litre infusion bags
60589	Warfarin 500microgram tablets (Actavis UK Ltd)

For peer review only - http://bmjopen.bmj.com/site/about/guidelines.xhtml

60949	Warfarin 5mg/5ml oral suspension
61949	Fondaparinux sodium 10mg/0.8ml solution for injection pre-filled syringes
62150	Rivaroxaban 2.5mg tablets
62309	Warfarin 500microgram tablets (Kent Pharmaceuticals Ltd)
62310	Warfarin 500microgram tablets (AMCo)
62856	Tinzaparin sodium 12,000units/0.6ml solution for injection pre-filled syringes
62902	Tinzaparin sodium 16,000units/0.8ml solution for injection pre-filled syringes
62959	Heparin calcium 5,000units/0.2ml solution for injection ampoules (A A H Pharmaceuticals Ltd)
63071	Warfarin 4mg tablets
63101	Tinzaparin sodium 8,000units/0.4ml solution for injection pre-filled syringes
63146	Heparin sodium 20,000units/20ml solution for injection ampoules
63169	Innohep 12,000units/0.6ml solution for injection pre-filled syringes (LEO Pharma)
63297	Heparin sodium 5,000units/5ml solution for injection vials (LEO Pharma)
63440	Epoprostenol 500microgram powder and solvent (pH10.5) for solution for infusion vials
63571	Innohep 16,000units/0.8ml solution for injection pre-filled syringes (LEO Pharma)
64133	Heparin sodium 5,000units/0.2ml solution for injection ampoules (A A H Pharmaceuticals Ltd)
64315	Anticoagulant solution ACD-A 500ml bags (Haemonetics Ltd)
64500	Xarelto 2.5mg tablets (Bayer Plc)
64559	Heparin sodium 1,000units/1ml solution for injection ampoules (A A H Pharmaceuticals Ltd)
64581	Innohep 8,000units/0.4ml solution for injection pre-filled syringes (LEO Pharma)
64678	Edoxaban 60mg tablets
64969	Clexane 20mg/0.2ml solution for injection pre-filled syringes (Sigma Pharmaceuticals Plc)
64998	Epoprostenol 1.5mg powder and solvent (pH10.5) for solution for infusion vials
65247	Edoxaban 30mg tablets
65285	Warfarin 1mg tablets (Crescent Pharma Ltd)
65496	Warfarin 500microgram tablets (Phoenix Healthcare Distribution Ltd)
65538	Elmiron 100mg capsules (Imported (United States))
65746	Warfarin 500microgram tablets (DE Pharmaceuticals)
65850	Lixiana 60mg tablets (Daiichi Sankyo UK Ltd)
65876	Edoxaban 15mg tablets
66286	Warfarin 2.5mg/5ml oral solution
66529	Lixiana 30mg tablets (Daiichi Sankyo UK Ltd)
66570	Warfarin 1mg tablets (Waymade Healthcare Plc)
68591	Warfarin 500microgram tablets (Alliance Healthcare (Distribution) Ltd)
68667	Warfarin 5mg capsules
68795	Warfarin 1mg capsules
69128	Warfarin 500micrograms/5ml oral solution
69194	Heparin low molecular weight 5000iu/0.2ml Sterile solution
70831	Phenindione 50mg tablets (AMCo)
70866	Inhixa 40mg/0.4ml solution for injection pre-filled syringes (Techdow Pharma England Ltd)

For peer review only

71132	Clexane 20mg/0.2ml solution for injection pre-filled syringes (DE Pharmaceuticals)
71196	Warfarin 1.5mg/5ml oral solution
71274	Inhixa 60mg/0.6ml solution for injection pre-filled syringes (Techdow Pharma England Ltd)
71303	Rivaroxaban 15mg tablets and Rivaroxaban 20mg tablets
71386	Warfarin 1mg/5ml oral solution (Special Order)

For peer review only

BMJ Open

Comprehensive ascertainment of bleeding in patients prescribed different combinations of dual antiplatelet therapy (DAPT) and triple therapy (TT) in the UK: study protocol for three population-based cohort studies emulating 'target trials' (the ADAPTT Study)

Journal:	*BMJ Open*
Manuscript ID	bmjopen-2019-029388.R1
Article Type:	Protocol
Date Submitted by the Author:	04-Mar-2019

Complete List of Authors:	Pufulete, Maria; University of Bristol,<br>Harris, Jessica; University of Bristol, Clinical Trials and Evaluation Unit, Bristol Medical School, Level 7, Bristol Royal Infirmary<br>Sterne, Jonathan; University of Bristol, Dept of Social Medicine<br>Johnson, Thomas; University Hospitals Bristol NHS Foundation Trust, Bristol heart Institute<br>Lasserson, Daniel; University of Birmingham, Institute of Applied Health Research; University of Oxford, Nuffield Department of Medicine<br>Mumford, Andrew; University Hospitals Bristol NHS Foundation Trust, Department of Haematology<br>Doble, Brett; University of Oxford, Nuffield Department of Population Health<br>Wordsworth, Sarah; University of Oxford, University of Oxford<br>Benedetto, Umberto; University of Bristol, Clinical Sciences<br>Rogers, Chris; university of bristol, Clinical Science South Bristol<br>Loke, Yoon; University of East Anglia, Norwich Medical School<br>Pithara, Christalla; University of Bristol, CLAHRC West, 9th Floor, Whitefriars BS1 2NT<br>Redwood, Sabi; Bristol, Population Health Sciences<br>Reeves, Barnaby; University of Bristol, Bristol Heart Institute

**Primary Subject Heading**:	Cardiovascular medicine
Secondary Subject Heading:	Epidemiology, Health services research
Keywords:	Dual antiplatelet therapy (DAPT), Acute coronary syndrome (ACS), Percutaneous coronary intervention (PCI), Coronary artery bypass grafting (CABG), Clinical Practice Research Datalink (CPRD), Hospital Episode Statistics (HES)

SCHOLARONE™
Manuscripts

Comprehensive ascertainment of bleeding in patients prescribed different combinations of dual antiplatelet therapy (DAPT) and triple therapy (TT) in the UK: study protocol for three population-based cohort studies emulating 'target trials' (the ADAPTT Study)

Maria Pufulete[1*], Jessica Harris[1], Jonathan A C Sterne[2], Thomas W. Johnson[3], Daniel Lasserson[4], Andrew Mumford[5], Brett Doble[6], Sarah Wordsworth[6], Umberto Benedetto[5], Chris A Rogers[1], Yoon Loke[7], Christalla Pithara[8], Sabi Redwood[8], Barnaby C Reeves[1]

[1] Clinical Trials and Evaluation Unit, Bristol Trials Centre, University of Bristol, Bristol, UK

[2] NIHR Biomedical Research Centre, Department of Population Health Sciences, University of Bristol, Bristol, UK

[3] Bristol Heart Institute, Bristol, UK

[4] Institute of Applied Health Research, University of Birmingham, Birmingham, UK

[5] Bristol Medical School, University of Bristol, Bristol, UK

[6] Health Economics Research Centre, Nuffield Department of Population Health, University of Oxford, Oxford UK

[7] Norwich Medical School, University of East Anglia, Norwich, UK

[8] National Institute for Health Research Collaboration for Leadership in Applied Health Research and Care West (NIHR CLAHRC West), Bristol, UK

[*] **Correspondence to**: Maria Pufulete, Clinical Trials and Evaluation Unit, University of Bristol, Level 7, Bristol Royal Infirmary, Queen's Building, Bristol, United Kingdom. Tel. +44 117 3423284. Fax. +44 117 3423288. Email: maria.pufulete@bristol.ac.uk

Key words: Dual antiplatelet therapy (DAPT); acute coronary syndrome (ACS); percutaneous coronary intervention (PCI), coronary artery bypass grafting (CABG); bleeding; Clinical Practice Research Datalink (CPRD); Hospital Episode Statistics (HES).

Abbreviations: ACS, acute coronary syndrome; AP, aspirin and prasugrel; AT, aspirin and ticagrelor; BARC, Bleeding Academic Research Consortium; CABG, coronary artery bypass grafting; CI, confidence interval; CIPS, Continuous inpatient stays; CPRD, Clinical Practice Research Datalink; DAPT, dual antiplatelet therapy; GP, General Practitioner; HES; Hospital Episode Statistics; HR, hazard ratios, ICD, International Classification of Diseases; MI, myocardial infarction; NICE, National Institute for Health and Care Excellence; NIHR, National Institute for Health Research; NHS, National Health Service; ONS, Office for

For peer review only - http://bmjopen.bmj.com/site/about/guidelines.xhtml

National Statistics; OPCS, Office of Population Censuses and Surveys; PCI, percutaneous coronary intervention; RCT, Randomized controlled trial; RR, relative risk; TT, triple therapy.

Word count: 3468 words

Number of tables: 2

Number of figures: 2

For peer review only

For peer review only - http://bmjopen.bmj.com/site/about/guidelines.xhtml

Abstract

INTRODUCTION: 'Real world' bleeding in patients exposed to different regimens of dual antiplatelet therapy (DAPT) and triple therapy (TT, DAPT plus an anticoagulant) have a clinical and economic impact but have not been previously quantified.

METHODS AND ANALYSIS: We will use linked Clinical Practice Research Datalink (CPRD) and Hospital Episode Statistics (HES) data to assemble populations eligible for three "target trials" in patient groups: percutaneous coronary intervention (PCI); coronary artery bypass grafting (CABG); conservatively managed (medication only) acute coronary syndrome (ACS). Patients ≥18 years old will be eligible if, in CPRD records, they have: ≥1 year of data before the index event; no prescription for DAPT or anticoagulants in the preceding three months; a prescription for aspirin or DAPT within two months after discharge from the index event. The primary outcome will be any bleeding event (CPRD or HES) up to 12 months after the index event. We will estimate adjusted hazard ratios for time to first bleeding event comparing: aspirin and clopidogrel (reference) vs. aspirin and prasugrel or aspirin and ticagrelor after PCI; and aspirin (reference) vs. aspirin and clopidogrel after CABG and ACS. We will describe rates of bleeding in patients prescribed triple therapy (DAPT plus an anticoagulant). Potential confounders will be identified systematically using literature review, semi-structured interviews with clinicians and a short survey of clinicians. We will conduct sensitivity analyses addressing the robustness of results to the study's main limitation – that we will not be able to identify the intervention group for patients whose bleeding event occurs before a DAPT prescription in CPRD.

ETHICS AND DISSEMINATION: This protocol was approved by the Independent Scientific Advisory Committee for the UK Medicines and Healthcare Products Regulatory Agency Database Research (protocol 16_126R) and the South West Cornwall and Plymouth Research Ethics Committee (17/SW/0092). The findings will be presented in peer-reviewed journals, lay summaries, and briefing papers to commissioners/other stakeholders.

ISRCTN: 76607611

For peer review only - http://bmjopen.bmj.com/site/about/guidelines.xhtml

Strengths and limitations of this study

- We designed our study using the framework recommended by the Cochrane Bias and Non-Randomized Studies Methods Groups for establishing appropriate patient populations, interventions and follow-up to emulate three hypothetical RCTs (target trials).
- We will identify potential confounders systematically using literature review, semi-structured interviews with clinicians (cardiologists, cardiac surgeons and general practitioners) and a short survey with an additional group of clinicians.
- Because there are no medication data in HES, we will assume that patients' first DAPT prescription that appears in CPRD after their hospital admission is what they were prescribed at discharge.
- We will conduct sensitivity analyses to address the robustness of results to different assumptions about the unknown intervention group in patients who died or had a bleeding event before a DAPT prescription in CPRD.

For peer review only

For peer review only - http://bmjopen.bmj.com/site/about/guidelines.xhtml

Introduction

Dual antiplatelet therapy (DAPT), a combination of aspirin and either clopidogrel, prasugrel, or ticagrelor, is recommended for secondary prevention of ischaemic events (heart attack and stroke) in people with coronary artery disease. Guidelines recommend that patients are treated with DAPT for 6 to 12 months following myocardial infarction (MI) and coronary interventions (percutaneous coronary intervention, PCI, and coronary artery bypass grafting, CABG) [1-3] and support the use of the more potent antiplatelet inhibitors ticagrelor and prasugrel [2]. Antiplatelet agents reduce the risk of ischaemic events, by preventing the formation of clots in atherosclerotic coronary arteries and within stents (following PCI) or grafts (following CABG), but increase the risk of bleeding [4]. Randomized controlled trials (RCTs) have shown that adding clopidogrel to aspirin leads to 1% excess risk of major bleeding (requiring admission to hospital) compared with aspirin alone [5, 6]. Prasugrel and ticagrelor reduce the risk of ischemic events further but also further increase the risk of bleeding [7]. Some patients (e.g. those with existing atrial fibrillation or those who develop atrial fibrillation after PCI, CABG or ACS) are prescribed an anticoagulant (e.g. warfarin, dabigatran, rivaroxaban, apixaban) in addition to DAPT (triple therapy, TT), which further increases the risk of bleeding.

"Real world" bleeding events that do not require any intervention are likely to be much more frequent than those reported in RCTs, which excluded patients at high risk of bleeding and mainly reported only on major bleeding. Bleeding events that do not result in hospitalisation are largely managed in primary care and may have a significant clinical and economic impact [8]. Minor and nuisance bleeding (nose and gum bleeds, bruising and prolonged bleeding from cuts) may also reduce adherence to DAPT and therefore the benefit of DAPT in non-adherent patients [9] who are at increased risk of a secondary ischaemic coronary episode [10]. Only three studies have reported the incidence and consequences of nuisance bleeding after DAPT [11-13]; these suggest that nuisance bleeding is common (affecting 29-38% of patients) and impacts on adherence (11% of patients in one study discontinued clopidogrel [12]). A nested case control study using the Health Improvement Network (a UK primary care database) reported an increased risk of upper gastrointestinal bleeding with clopidogrel and aspirin compared with aspirin alone (relative risk [RR], 2.08; 95% confidence interval [CI], 1.34 to 3.21) [14].

For peer review only - http://bmjopen.bmj.com/site/about/guidelines.xhtml

The economic impact of bleeding events associated with DAPT is also poorly characterized, in particular for minor bleeding events and their impact on health-related quality of life [8].This is not surprising given that health economic analyses often lack detailed data on adverse effects of interventions, despite consensus that such effects should be considered [15, 16]. To ensure appropriate decisions are made about which DAPT regimens to use in clinical practice, the health and resource use consequences of minor and major bleeding events should be incorporated into assessments of cost-effectiveness. For DAPT, this entails accounting for uncertainty in the absolute risk of bleeding, the impact of different bleeding events on health-related quality of life and treatment adherence and subsequent risk of secondary ischaemic events, and the cost implications of managing these bleeding events.

We propose to use Hospital Episode Statistics (HES) and the Clinical Practice Research Datalink (CPRD) databases to estimate the incidence of all bleeding events occurring in patients prescribed different DAPT or TT regimens after undergoing coronary interventions (PCI and CABG) and in conservatively-managed ACS patients. Our study will also provide parameter estimates to update existing cost effectiveness models. We will use the framework recommended by the Cochrane Bias and Non-Randomized Studies Methods Groups for establishing appropriate patient populations, interventions and follow-up to emulate the following three hypothetical RCTs (hereafter referred to as the target trials, **Table 1**) [17]:

1. In patients who have undergone PCI, estimate the effect on bleeding events of assignment to aspirin and clopidogrel (reference) versus aspirin and prasugrel or aspirin and ticagrelor.
2. In patients who have undergone CABG, estimate the effect on bleeding events of assignment to aspirin (reference) versus aspirin and clopidogrel.
3. In patients who are conservatively-managed patients after presenting with ACS, estimate the effect on bleeding events of assignment to aspirin (reference) versus aspirin and clopidogrel.

For peer review only - http://bmjopen.bmj.com/site/about/guidelines.xhtml

Methods

Data sources

CPRD is a database of primary care electronic health record data (available online via CPRD GOLD) from participating general practices, covering 7% of the UK population [18]. Patients included in CPRD are largely representative of the UK population in terms of age, sex, ethnicity, and body mass index. HES covers all hospital admissions for all English patients whose treatment is funded by the UK National Health Service (NHS), whether treated by the NHS or by independent providers [19]. Seventy-five percent of English general practices included in CPRD are linked to HES data [18]. We obtained data from 1 April 2009 to 31 July 2017; this period covers the introduction of the newer antiplatelet agents prasugrel and ticagrelor. This study protocol has been approved by the Independent Scientific Advisory Committee (ISAC) of the CPRD (protocol number 16_126R).

Study populations

Eligibility and exclusion criteria for the three target trials (for patients undergoing PCI, patients undergoing CABG; or patients hospitalised and conservatively-managed for ACS) are listed in **Table 1**. We will identify eligible patients who are included in CPRD and eligible for linkage with HES and Office for National Statistics (ONS) mortality data (because they have a valid NHS number and are registered at a practice that was participating in the linkage programme). Patients are included if they had a PCI, CABG or ACS (index event) a record in HES during the study period (1 April 2010 – 1 January 2017), and have at least 1 year of linked CPRD-HES data before the date of their index event. They must also have been prescribed one of the treatment regimens being compared in the target trial corresponding to their index event. One year's data preceding eligibility for the target trial is adequate to apply most of the exclusion criteria and determine co-morbidities and medication history: such information would be collected at baseline in a randomized trial. The following Office of Population Censuses and Surveys (OPCS) procedure codes (PCI and CABG) and International Classification of Diseases (ICD-10) codes (ACS no procedure) will be used to identify patients: PCI, K49, K50 & K75; CABG, K40, K41, K42, K43, K44, K45 & K46; ACS without a procedure, I20.0, I21, I22, I24.9 (with no OPCS code for PCI or CABG in the same hospital admission). **Figure 1** shows full details of the inclusion and exclusion criteria.

For peer review only - http://bmjopen.bmj.com/site/about/guidelines.xhtml

Table 1. Summary of three target trials and how observational data will be used to emulate these

PICO component	Target trial	Issues in emulating the target trial using observational data
Eligibility criteria	**Target trial 1 (PCI)**<br>Consecutive patients (age ≥18 years) undergoing PCI (emergency or elective). Exclusions: DAPT or anticoagulant use in the previous 3 months; major bleed requiring hospitalization in previous 12 months; renal failure requiring dialysis; intolerance/allergy to aspirin, clopidogrel, prasugrel or ticagrelor.<br>**Target trial 2 (CABG)**<br>Consecutive patients (age ≥18 years) undergoing CABG (urgent and elective). Exclusions: DAPT or anticoagulant use in the previous 3 months; other concomitant cardiac surgery (e.g. valve surgery); major bleed requiring hospitalization in previous 12 months; renal failure requiring dialysis; intolerance/allergy to aspirin, clopidogrel, prasugrel or ticagrelor.<br>**Target trial 3 (conservatively-managed ACS)**<br>Consecutive patients (age ≥18 years) hospitalized for an acute coronary syndrome (ACS): myocardial infarction (MI) with or without ST-elevation or unstable angina. Exclusions: PCI or CABG performed at time of ACS diagnosis; major bleed requiring hospitalization in previous 12 months; renal failure requiring dialysis; intolerance/allergy to aspirin, clopidogrel, prasugrel or ticagrelor.	CPRD-HES linked dataset contains information that allows us to identify all eligible patients for the three target trials. The study period is April 2009-July 2017. All eligible patients will have sufficient data (1 year) preceding their index event to apply the exclusion criteria and characterize the population (e.g. co-morbidities) and sufficient follow-up data (1 year) to identify outcomes. It is not possible to capture intolerance/allergy to aspirin, clopidogrel, prasugrel or ticagrelor.
Interventions	**Target trial 1 (PCI)**<br>Clopidogrel (75mg daily) or prasugrel (5mg or 10mg daily) or ticagrelor (90mg twice daily). All patients will receive aspirin (at a dose of 75mg daily, in line with current guidelines).<br>**Target trial 2 (CABG)**	Relevant interventions can be identified as CPRD has information on all medications (including doses) prescribed in primary care.

For peer review only - http://bmjopen.bmj.com/site/about/guidelines.xhtml

	Clopidogrel (75mg) in addition to aspirin (at a dose of 75mg daily, in line with current guidelines) or aspirin only (any dose, reflecting variation in usual care).<br>**Target trial 3 (conservatively-managed ACS)**<br>As for target trial 2.	
Assignment to interventions	Participants are assigned to DAPT interventions in hospital.	Participants enter the study at index procedure date for PCI and CABG, and episode start date for ACS, and will be assigned to DAPT interventions using first prescription in CPRD (within 2 months of hospitalization) as a proxy for what they were prescribed in hospital (there are no medications data in HES). This assignment will exclude a proportion of eligible patients (those who died or experienced a major bleed that caused them to stop DAPT, or patients who have no prescription for DAPT within the 2 month window); we will identify and describe the characteristics of these excluded patients.<br>In sensitivity analyses, we will address the robustness of results to different assumptions about the intervention group in those patients where the DAPT medication is unknown or a major bleed occurs prior to the first DAPT medication, by using multiple imputation models for handling missing data. Prior known information regarding the likely prescription based on patient characteristics or general policies will be incorporated in these analyses.
Follow-up	Starts at assignment to intervention and ends at first bleed or 12 months from assignment (whichever comes first).	Starts at time of hospitalization for PCI, CABG or ACS and ends at first bleed or 12 months from hospitalization (whichever comes first).
Primary outcome	Any bleed within 12 months of the start of DAPT (DAPT is prescribed at hospitalization for PCI, CABG or ACS).	Any bleed within 12 months of hospitalization for PCI, CABG or ACS.

For peer review only - http://bmjopen.bmj.com/site/about/guidelines.xhtml

Analysis	Intention to treat	According to first prescription for DAPT in CPRD

For peer review only

For peer review only - http://bmjopen.bmj.com/site/about/guidelines.xhtml

Interventions

The interventions of interest for the three target trials are shown in **Table 1**. Guidelines recommend low dose aspirin (75 to 100mg/d) plus either clopidogrel (75mg/d), prasugrel (5mg/d or 10mg/d), or ticagrelor (90mg twice/d) for PCI and conservatively-managed ACS patients. For PCI patients, the interventions of interest are aspirin and clopidogrel, aspirin and prasugrel, and aspirin and ticagrelor. In conservatively-managed ACS patients, clopidogrel is the most commonly prescribed second antiplatelet agent (in addition to aspirin) and a large proportion of patients are prescribed aspirin only, therefore the interventions of interest are aspirin only (75 to 100mg/d) and aspirin and clopidogrel. There is variation in aspirin prescription for CABG patients; some surgeons choose 75mg/d, others 150mg/d, or 300mg/d. surgeons may also prescribe an additional antiplatelet agent, most commonly clopidogrel. Therefore, the comparisons of interest in CABG patients are aspirin only (any dose, reflecting variations in usual care in different hospitals) and aspirin and clopidogrel (doses as for PCI). We have specified these comparisons based on preliminary feasibility counts from CPRD, which indicate that few CABG and conservatively-managed ACS patients are prescribed aspirin and prasugrel or aspirin and ticagrelor.

In the target trials the interventions would be assigned during the hospital stay, as soon as patients are eligible for antiplatelet therapy. Our observational dataset does not have information on medication given to patients at discharge, because HES does not include medications data. Therefore, the first time at which we have information on the antiplatelet regimen to which patients were assigned in hospital is when they receive their first primary care prescription/s for aspirin or DAPT, recorded in CPRD. It is reasonable to use these as a proxy for the medications that patients started in hospital, because patients' general practitioners are unlikely to change the prescriptions that were started in hospital.

We will classify patients according to the first prescription recorded in CPRD during the first two months after hospitalization for PCI, CABG, or ACS. This two-month window is based on variability in the amount of DAPT medication provided to patients in hospital following their PCI, CABG or ACS and hence variability in the time when they first request a repeat prescription from their general practice. A preliminary investigation showed that more than 80% of eligible patients have a prescription for one or more antiplatelet agents during this time period. If a patient only has a prescription for aspirin during the 2-month window after

For peer review only - http://bmjopen.bmj.com/site/about/guidelines.xhtml

hospital discharge, they will be assigned to an aspirin only intervention. If patients also receives a prescription for clopidogrel, prasugrel or ticagrelor, they will be assigned to aspirin/clopidogrel, aspirin/prasugrel or aspirin/ticagrelor. If there is a prescription for more than one additional antiplatelet agent in the 2-month window, we will assign the patient to an intervention based on the agent prescribed first. For example, if a patient has an aspirin prescription and a prescription for clopidogrel before a prescription for ticagrelor, the patient will be assigned to the aspirin/clopidogrel intervention. Patients with no prescriptions in CPRD for aspirin, or aspirin and clopidogrel, prasugrel or ticagrelor within the 2-month window will be excluded from the main analysis. We will conduct sensitivity analyses of all eligible patients including those with unknown DAPT regimens or a major bleed prior to first DAPT prescription, by estimating assignment to DAPT interventions for those with no prescription data using multiple imputation based on a range of assumptions [20].

Outcomes

The primary outcome will be any bleeding event, classified as type 2-5 by the Bleeding Academic Research Consortium (BARC) bleeding scale [21]. For each patient, we will identify all bleeding events in HES and CPRD during follow-up. We will not be able to identify BARC type 1 bleeding events (bleeding that is not actionable and does not cause the patient to seek treatment) as type 1 assumes there is no interaction with the health system or healthcare professionals therefore no bleed event will be recorded in HES or CPRD. We have specified a comprehensive list of bleeding codes in CPRD and HES (see **Appendix 1**). These will be categorised according to anatomical site for descriptive purposes. Secondary outcomes will be: any major bleeding event; any minor bleeding event; all-cause mortality; cardiovascular mortality; mortality from bleeding (these will be identified from linked ONS data); hospital admission for a bleeding event (inpatient HES); MI; stroke; additional coronary intervention.

Follow up

The start of follow up (the index event) will be the date of the index hospital procedure (PCI, CABG) or start date of the hospital episode that contains the ACS diagnosis (ACS). Patients will be followed up until 12 months after the index event, since DAPT is prescribed for 12 months in accordance to guidelines.

For peer review only - http://bmjopen.bmj.com/site/about/guidelines.xhtml

Confounding and co-interventions

Potential confounders (variables that predict both risk of bleeding and intervention group) will be specified *a priori* [22, 23]. We will identify confounders and co-interventions using literature review and clinician expertise as recommended by the Cochrane Bias and Non-Randomized Studies Methods Groups [17]. We will carry out a comprehensive and systematic literature search to identify all RCT and cohort studies of DAPT interventions, or cohort studies that identify predictors of bleeding. The literature searches for the review are included in **Appendix 2**. Abstracts will be screened by one researcher and full text papers will be obtained. Data on confounders and co-interventions will be extracted by two researchers independently using a data extraction form specifically designed for the study; variables extracted will include study characteristics, population characteristics (reported in the tables of baseline characteristics), factors adjusted for in the statistical analyses, and factors reported to predict risk of bleeding in our populations. We will not perform a risk of bias assessment because the aim of the review is only descriptive (i.e. the output will be lists of confounders and co-interventions) and there are no established criteria for assessing the validity with which primary researchers consider potential confounders and co-interventions; therefore it would be inappropriate to apply a risk-of-bias tool for studies estimating a treatment effect. We will use "saturation" as a criterion for discontinuing data collection, defined as review of 10 consecutive studies without identifying an additional confounder/co-intervention.

In parallel, we will conduct semi quantitative interviews with six clinicians in each of three groups: cardiologists; cardiac surgeons and general practitioners (GPs, to determine whether DAPT prescriptions are changed in primary care). The main aim of the clinician interviews is to understand DAPT prescribing practice in the UK and identify the factors (relating to patients, centres and prescribing practices of the individual doctors) that influence the decision about which antiplatelet regimen to prescribe. All factors that influence DAPT prescribing (confounders) identified from the literature review and clinician interviews will be combined in a short survey (SurveyMonkey®). The survey questionnaire will be administered online to all consultant members of the British Cardiovascular Society (cardiologists) andthe Society for Cardiothoracic Surgery (cardiac surgeons). The survey will be either emailed to all members individually (if the professional bodies agree) or advertised in weekly/monthly newsletters. Confounders will be grouped in confounding domains [17].

For peer review only - http://bmjopen.bmj.com/site/about/guidelines.xhtml

We will attempt to identify each potential confounder (identified through the literature review, clinician interviews and survey) in the CPRD or HES dataset but acknowledge that there may be missing data across patients (and time) for some confounders.

Sample size

Estimated rates of bleeding with the different therapies are 5% for aspirin, 9% for aspirin/clopidogrel, and 12% for aspirin/prasugrel and aspirin/ticagrelor [5, 6, 24, 25]. Preliminary feasibility counts provided by CPRD suggest that there will at least the following numbers of patients eligible for each target trial:

- PCI: aspirin/clopidogrel (reference, 6738 patients) vs aspirin/prasugrel (842 patients) or aspirin/ticagrelor (770 patients)
- CABG: aspirin (reference, 2556 patients) vs aspirin/clopidogrel (595 patients)
- Conservatively-managed ACS: aspirin (reference, 8148 patients) vs aspirin/clopidogrel (3082 patients)

These estimates give expected event rates of at least 700 for PCI, 180 for CABG and 680 for ACS, assuming a ratio of 8:1 (aspirin/clopidogrel:aspirin/prasugrel or aspirin/clopidogrel:aspirin/ticagrelor) for PCI, 4:1 (aspirin:aspirin/clopidogrel) for CABG and 2.5:1 (aspirin:aspirin/clopidogrel) for ACS. The hazard ratios detectable with 90% and 80% power at the 5% statistical significance, assuming the group ratios given above are shown in **Table 2**. The correlation of the DAPT with other covariates adjusted for is unknown and we assessed the impact of a range of correlations (0, 0.3 and 0.5).

Table 2. Hazard ratios for a range of correlations for PCI, CABG and ACS

Ratio of presence: absence of covariate	Squared correlation with other covariates	Hazard ratio detectable 90% power	80% power
**PCI**			
8:1	0 (i.e. unadjusted)	1.48	1.41
	0.3	1.60	1.50
	0.5	1.74	1.62
**CABG**			

For peer review only - http://bmjopen.bmj.com/site/about/guidelines.xhtml

4:1	0 (i.e. unadjusted)	1.83	1.69
	0.3	2.06	1.87
	0.5	2.35	2.10
**Conservatively-managed ACS**			
2.5:1	0 (i.e. unadjusted)	1.32	1.27
	0.3	1.39	1.33
	0.5	1.48	1.40

Statistical analyses

We will describe temporal changes in DAPT prescribing and bleeding for PCI, CABG and ACS populations. We will use descriptive statistics to summarize the characteristics of the different intervention groups and standardised mean differences to compare them. We will estimate rates of bleeding (number of events/person time) with 95% confidence intervals (CIs) for each group. We will separate major and minor bleeding since adverse events of each type have different health and resource use consequences.

Analyses will estimate the effects of assigned intervention (analogous to an intention-to-treat analysis of a randomized trial) for the antiplatelet regimens corresponding to the first prescription of aspirin or DAPT in CPRD (see Interventions). We will use parametric survival models to estimate adjusted hazard ratios (HRs) with 95% CIs for the time to first bleeding event, comparing intervention groups for each target trial. Exploratory analyses, including assessment of proportional hazards assumptions, will be used to inform the choice of survival distribution (e.g. Weibull). Estimated time-dependent event probabilities will be used to update existing cost-effectiveness models [26]. The confounding factors to be included in the model (which will be identified as described previously and grouped into confounding domains from our dataset), the modelling strategy and the approach to handling correlated covariates will be documented in a data analysis plan. Participants free from a bleeding event will be censored at 12 months after the index event. For secondary endpoints we will use survival models to estimate adjusted hazard ratios (HRs) with 95% CIs for time

For peer review only - http://bmjopen.bmj.com/site/about/guidelines.xhtml

to first event. For mortality outcomes, we will take account of the competing risks of death due to other causes.

We will perform three sets of sensitivity analyses:

1. We will address the unknown intervention group of eligible patients who have no prescription data and therefore cannot be assigned to an intervention (i.e. those that died before receiving their first prescription, had a major bleed which caused them to stop DAPT, or have no aspirin/DAPT prescription recorded in CPRD within the 2 month window). These analyses will be undertaken using multiple imputation methods to deal with missing information on DAPT medication and will take patient characteristics, procedure/diagnosis and general medication policies into account, using a range of assumptions.
2. We will address the possibility that some minor bleeding events are not documented in CPRD but nevertheless prompt medication changes. We anticipate that most bleeding events will occur soon after the index event and before any medication change. Medication changes will be described relative to bleeding events observed (e.g. before event, after event, no bleeding event observed). If a substantial proportion (>10%) of people change medication before their first bleeding event, we will perform a sensitivity analysis excluding these patients.
3. We will assess the sensitivity of our findings when the analysis is restricted to a sub-population of patients at low risk of bleeding (i.e. excluding people at high risk for bleeding) who, we hypothesise, have the lowest risk of residual confounding.

We will attempt to identify an instrumental variable (e.g. clinician/GP practice prescribing practice) to control for confounding by indication. If we are successful we will repeat the above analyses for the PCI, CABG and ACS populations.

We will explore the consistency of treatment effect estimates in the following subgroups: ACS vs non-ACS (PCI and CABG populations); diabetic vs non-diabetic; chronic kidney disease vs. non-chronic kidney disease; concurrent prescription for proton pump inhibitors vs. no prescription for proton pump inhibitors. All subgroups will be defined in the data analysis plan and chosen based on the characteristics of the analysis populations before carrying out any analyses by intervention group. We will report p values from tests of interaction.

For peer review only - http://bmjopen.bmj.com/site/about/guidelines.xhtml

Triple therapy (TT)

We will establish additional groups of patients (PCI or CABG or conservatively-managed ACS) based on triple therapy (TT) prescription. The main reason for prescribing an anticoagulant is to treat atrial fibrillation in a patient otherwise eligible for either of the three target trials. Atrial fibrillation can precede (long-term anticoagulation for pre-existing atrial fibrillation) or follow the index event (anticoagulation for new-onset atrial fibrillation). **Figure 2** shows how the patient groups receiving TT will be constructed.

We will identify patients on long-term anticoagulation by identifying prescriptions of oral anticoagulants (warfarin, dabigatran, rivaroxaban, apixaban) in the 3 months before their index event and a concomitant prescription of any anticoagulant (TT) with DAPT and in the first two months after the index admission. Patients who develop atrial fibrillation after the index event will be identified from any new anticoagulant prescription during follow-up. We have compiled a list of all drug codes in CPRD for antiplatelet agents (aspirin, clopidogrel, prasugrel and ticagrelor) and anticoagulants (warfarin, dabigatran, rivaroxaban, apixaban) (see **Appendix 3**).

In patients receiving various TT regimens (grouped by pre-index event anticoagulant prescription and post-index event anticoagulant prescription in PCI, CABG and ACS separately, see Figure 2), we will describe rates of bleeding (number of events/person time) with 95% CIs for each group. We have not specified comparison groups because we know there is large variation in prescribing of anticoagulants;TT is usually prescribed for a relatively short period of time (1-6 months, depending on a patient's individual risks of thrombosis and bleeding) after which an antiplatelet agent (usually aspirin) is removed and patients continue to receive an anticoagulant and single antiplatelet for the remainder of the 12 months after the index admission.

Patient and public involvement

We set up a patient advisory group comprising of patients from our three cohorts (PCI, CABG and conservatively-managed ACS). The group was consulted about several aspects of the proposed study. Group members confirmed the importance of the research topic and and agreed that the choice of antiplatelet regimen should be based on shared decision making,

For peer review only - http://bmjopen.bmj.com/site/about/guidelines.xhtml

with clinicians and patients weighing the potential benefits against the adverse side effects and practical inconvenience. Our PPI group will attend regular meetings to discuss results from the study and will also contribute and collaborate on the development of our results dissemination strategy.

Ethics and dissemination

This study protocol has approval from the Independent Scientific Advisory Committee for the UK Medicines and Healthcare products Regulatory Agency Database Research (protocol 16_126R). We also obtained ethical approval for the semi-structured interviews with clinicians and survey from South West Cornwall and Plymouth Research Ethics Committee, 17/SW/0092. The findings will be presented at national/international conferences, published in peer-reviewed academic journals and accessible formats in newsletters to patients (where available). The findings will also be reported as a briefing paper to commissioners (e.g. commissioning groups, National Institute for Health and Care Excellence, NICE) and to other health care stakeholders with an interest in the research through the Cardiac & Stroke Networks. We will also present and discuss our results at local CPRD working groups to disseminate out methods to other researchers using the CPRD database.

Author contributions

MP: designed and will conduct the study and wrote the manuscript.

JH: study statistician responsible for data linkage and analysis.

JACS: provided strategic direction with respect to study design and analysis.

TJ: conceived the research question and provided cardiology expertise.

DL: provided general practice expertise, clinical epidemiological expertise

AM: conceived the research question and provided haematology expertise.

BD: provided health economics expertise.

SW: provided health economics expertise.

UB: provided cardiac surgery expertise.

CAR: provided guidance with respect to the statistical analysis.

YL: provided pharmacoepidemiology expertise.

CP: responsible for conduct of the qualitative study with clinicians.

For peer review only - http://bmjopen.bmj.com/site/about/guidelines.xhtml

SR: will oversee conduct and reporting of the qualitative study with clinicians.

BCR: provided strategic direction with respect to study design and analysis.

All authors read and approved the final version of the manuscript.

Funding

This study is funded by the National Institute for Health Research (NIHR) Health Technology Assessment (HTA 14/192/89). The British Heart Foundation and NIHR Bristol Biomedical Research Unit for Cardiovascular Disease funded some staff time (MP, JH, BCR). The views expressed are those of the authors and not necessarily those of the NHS, the NIHR or the Department of Health.

Competing interests

TJ has received speaker & consultancy fees from Astra Zeneca & Daichi-Sankyo

None of the other authors have competing interests to declare.

Acknowledgements

We would like to thank Taria Murray-Thomas, senior researcher at CRPD, who assisted with the data requests. We would also like to thank members of our PPI group and the PPI lead, Dr Noreen Hopewell-Kelly, for their input into the design of the study. This study was designed and delivered in collaboration with the Clinical Trials and Evaluation Unit (CTEU), a UKCRC registered clinical trials unit which, as part of the Bristol Trials Centre, is in receipt of National Institute for Health Research CTU support funding.

References

1. http://www.nice.org.uk/guidance/cg172/resources/guidance-mi-secondary-prevention-pdf NCG: 2013.
2. Valgimigli M, Bueno H, Byrne RA, Collet JP, Costa F, Jeppsson A, Juni P, Kastrati A, Kolh P, Mauri L *et al*: **2017 ESC focused update on dual antiplatelet therapy in coronary artery disease developed in collaboration with EACTS: The Task Force for dual antiplatelet therapy in coronary artery disease of the European Society of Cardiology (ESC) and of the European Association for Cardio-Thoracic Surgery (EACTS)**. *European heart journal* 2018, **39**(3):213-260.
3. Wijns W, Kolh P, Danchin N, Di Mario C, Falk V, Folliguet T, Garg S, Huber K, James S, Knuuti J *et al*: **Guidelines on myocardial revascularization**. *European heart journal* 2010, **31**(20):2501-2555.

For peer review only - http://bmjopen.bmj.com/site/about/guidelines.xhtml

4. Tada T, Natsuaki M, Morimoto T, Furukawa Y, Nakagawa Y, Byrne RA, Kastrati A, Kadota K, Iwabuchi M, Shizuta S *et al*: **Duration of dual antiplatelet therapy and long-term clinical outcome after coronary drug-eluting stent implantation: landmark analyses from the CREDO-Kyoto PCI/CABG Registry Cohort-2**. *Circulation Cardiovascular interventions* 2012, **5**(3):381-391.
5. Main C, Palmer S, Griffin S, Jones L, Orton V, Sculpher M, Henderson R, Sudlow C, Hawkins N, Riemsma R: **Clopidogrel used in combination with aspirin compared with aspirin alone in the treatment of non-ST-segment-elevation acute coronary syndromes: a systematic review and economic evaluation**. *Health technology assessment (Winchester, England)* 2004, **8**(40):iii-iv, xv-xvi, 1-141.
6. Terpening C: **An appraisal of dual antiplatelet therapy with clopidogrel and aspirin for prevention of cardiovascular events**. *Journal of the American Board of Family Medicine : JABFM* 2009, **22**(1):51-56.
7. Pilgrim T, Windecker S: **Antiplatelet therapy for secondary prevention of coronary artery disease**. *Heart (British Cardiac Society)* 2014, **100**(22):1750-1756.
8. Doble B, Pufulete M, Harris J, Johnson T, Lasserson D, Reeves BC, Wordsworth S: **Health-related quality of life impact of minor and major bleeding events during dual antiplatelet therapy: A systematic review and patient preference elicitation study.** *Health & Quality of Life Outcomes* 2018, **16**:191.
9. Czarny MJ, Nathan AS, Yeh RW, Mauri L: **Adherence to dual antiplatelet therapy after coronary stenting: a systematic review**. *Clinical cardiology* 2014, **37**(8):505-513.
10. Cohen M: **Expanding the recognition and assessment of bleeding events associated with antiplatelet therapy in primary care**. *Mayo Clinic proceedings* 2009, **84**(2):149-160.
11. Ben-Dor I, Torguson R, Scheinowitz M, Li Y, Delhaye C, Wakabayashi K, Maluenda G, Syed AI, Collins SD, Gonzalez MA *et al*: **Incidence, correlates, and clinical impact of nuisance bleeding after antiplatelet therapy for patients with drug-eluting stents**. *American heart journal* 2010, **159**(5):871-875.
12. Roy P, Bonello L, Torguson R, de Labriolle A, Lemesle G, Slottow TL, Steinberg DH, Kaneshige K, Xue Z, Satler LF *et al*: **Impact of "nuisance" bleeding on clopidogrel compliance in patients undergoing intracoronary drug-eluting stent implantation**. *The American journal of cardiology* 2008, **102**(12):1614-1617.
13. Amin AP, Bachuwar A, Reid KJ, Chhatriwalla AK, Salisbury AC, Yeh RW, Kosiborod M, Wang TY, Alexander KP, Gosch K *et al*: **Nuisance bleeding with prolonged dual antiplatelet therapy after acute myocardial infarction and its impact on health status**. *Journal of the American College of Cardiology* 2013, **61**(21):2130-2138.
14. Garcia Rodriguez LA, Lin KJ, Hernandez-Diaz S, Johansson S: **Risk of upper gastrointestinal bleeding with low-dose acetylsalicylic acid alone and in combination with clopidogrel and other medications**. *Circulation* 2011, **123**(10):1108-1115.
15. Craig D, McDaid C, Fonseca T, Stock C, Duffy S, Woolacott N: **Are adverse effects incorporated in economic models? An initial review of current practice**. *Health technology assessment (Winchester, England)* 2009, **13**(62):1-71, 97-181, iii.
16. Heather EM, Payne K, Harrison M, Symmons DP: **Including adverse drug events in economic evaluations of anti-tumour necrosis factor-alpha drugs for adult rheumatoid arthritis: a systematic review of economic decision analytic models**. *PharmacoEconomics* 2014, **32**(2):109-134.
17. Sterne JA, Hernan MA, Reeves BC, Savovic J, Berkman ND, Viswanathan M, Henry D, Altman DG, Ansari MT, Boutron I *et al*: **ROBINS-I: a tool for assessing risk of bias in non-randomised studies of interventions**. *BMJ (Clinical research ed)* 2016, **355**:i4919.
18. Herrett E, Gallagher AM, Bhaskaran K, Forbes H, Mathur R, van Staa T, Smeeth L: **Data Resource Profile: Clinical Practice Research Datalink (CPRD)**. *International journal of epidemiology* 2015, **44**(3):827-836.

For peer review only - http://bmjopen.bmj.com/site/about/guidelines.xhtml

19. http://content.digital.nhs.uk/hes.
20. Sterne JA, White IR, Carlin JB, Spratt M, Royston P, Kenward MG, Wood AM, Carpenter JR: **Multiple imputation for missing data in epidemiological and clinical research: potential and pitfalls**. *BMJ (Clinical research ed)* 2009, **338**:b2393.
21. Mehran R, Rao SV, Bhatt DL, Gibson CM, Caixeta A, Eikelboom J, Kaul S, Wiviott SD, Menon V, Nikolsky E *et al*: **Standardized bleeding definitions for cardiovascular clinical trials: a consensus report from the Bleeding Academic Research Consortium**. *Circulation* 2011, **123**(23):2736-2747.
22. Reeves BC, Deeks JJ, Higgins JPT, Wells GA: **Chapter 13: Including non-randomized studies. In: Higgins JPT, Green S (editors), Cochrane Handbook for Systematic Reviews of Interventions. Version 5.0.2 [updated September 2009]. The Cochrane Collaboration, 2009.**
23. Reeves BC, Higgins JP, Ramsay C, Shea B, Tugwell P, Wells GA: **An introduction to methodological issues when including non-randomised studies in systematic reviews on the effects of interventions**. *Research synthesis methods* 2013, **4**(1):1-11.
24. Wiviott SD, Braunwald E, McCabe CH, Montalescot G, Ruzyllo W, Gottlieb S, Neumann FJ, Ardissino D, De Servi S, Murphy SA *et al*: **Prasugrel versus clopidogrel in patients with acute coronary syndromes**. *The New England journal of medicine* 2007, **357**(20):2001-2015.
25. Wallentin L, Becker RC, Budaj A, Cannon CP, Emanuelsson H, Held C, Horrow J, Husted S, James S, Katus H *et al*: **Ticagrelor versus clopidogrel in patients with acute coronary syndromes**. *The New England journal of medicine* 2009, **361**(11):1045-1057.
26. Briggs A, Claxton K, Sculpher M: **Decision Modelling for Health Economic Evaluation. New York, Oxford University Press Inc.** . 2006.

For peer review only

Figure 1. Study diagram describing the construction of the PCI, CABG and ACS (conservatively managed) populations.

Figure 2. Study diagram describing the construction of the triple therapy (TT) populations.

For peer review only

For peer review only - http://bmjopen.bmj.com/site/about/guidelines.xhtml

Figure 1. Study diagram describing the construction of the PCI, CABG and ACS (conservatively managed) populations

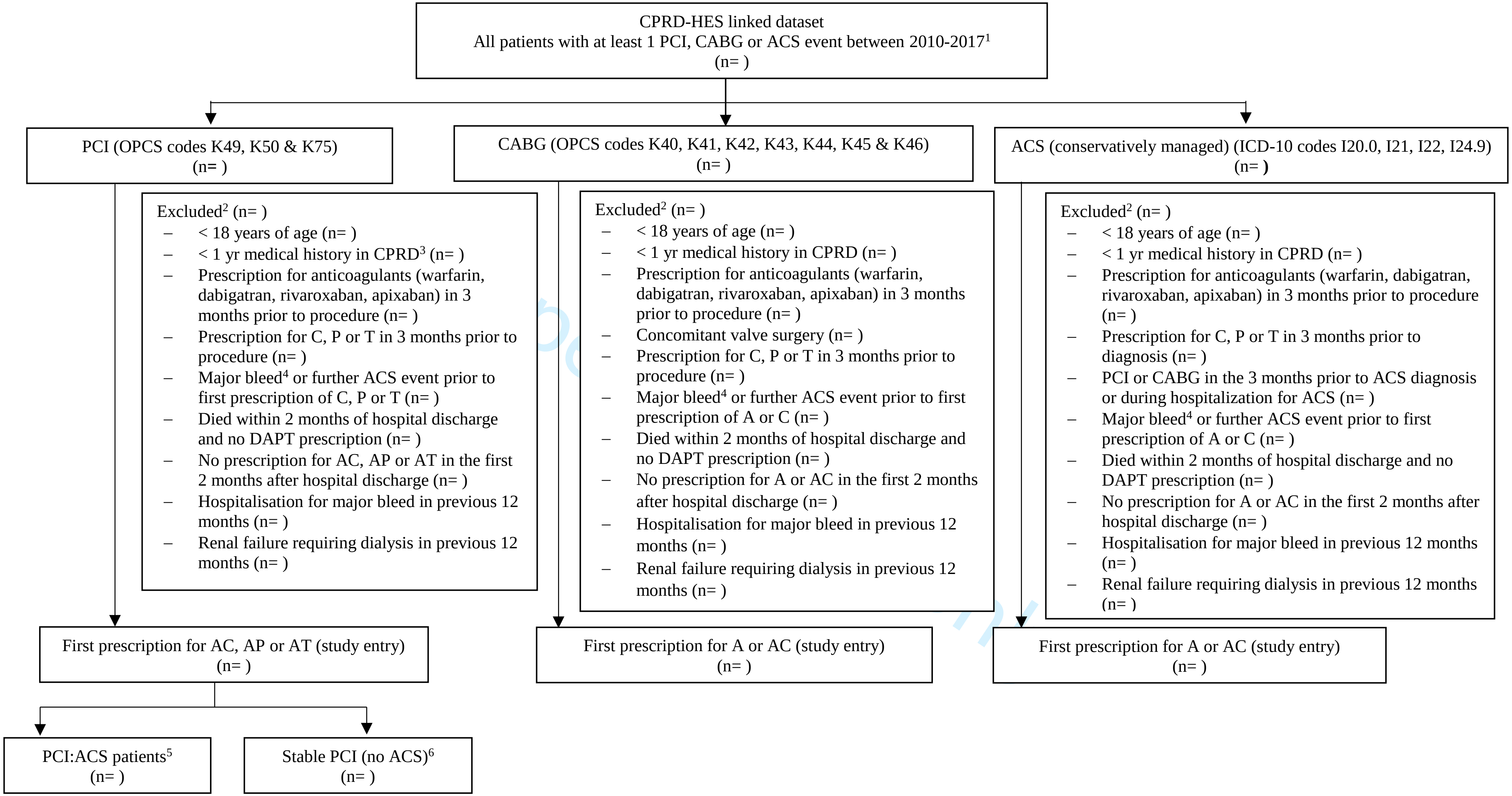

C: clopidogrel; P: prasugrel; T: ticagrelor; AC: aspirin + clopidogrel; AP: aspirin + prasugrel; AT: aspirin + ticagrelor
ACS: acute coronary syndrome; CABG: coronary artery bypass grafting; CPRD, Clinical Practice Research Datalink; HES; Hospital Episode Statistics; ICD International Classification of Diseases; OPCS, Office of Population Censuses and Surveys; PCI: percutaneous coronary intervention.

[1]Continuous inpatient stays (CIPS) that include both PCI and CABG are considered CABG; CIPS that include both ACS and PCI are considered PCI; CIPS that include both ACS and CABG are considered CABG. [2]Patients may be excluded for more than one reason. [3]At least one year of CPRD data available prior to event plus not "transferred out" before the event plus GP practice up to standard date precedes event date by at least 12 months. [4]Major bleed captured within hospital admission. [5]PCI and ACS in the same CIPS. [6]PCI event but no ACS within the same CIPS.

For peer review only - http://bmjopen.bmj.com/site/about/guidelines.xhtml

Figure 2. Study diagram describing the construction of the triple therapy (TT) populations

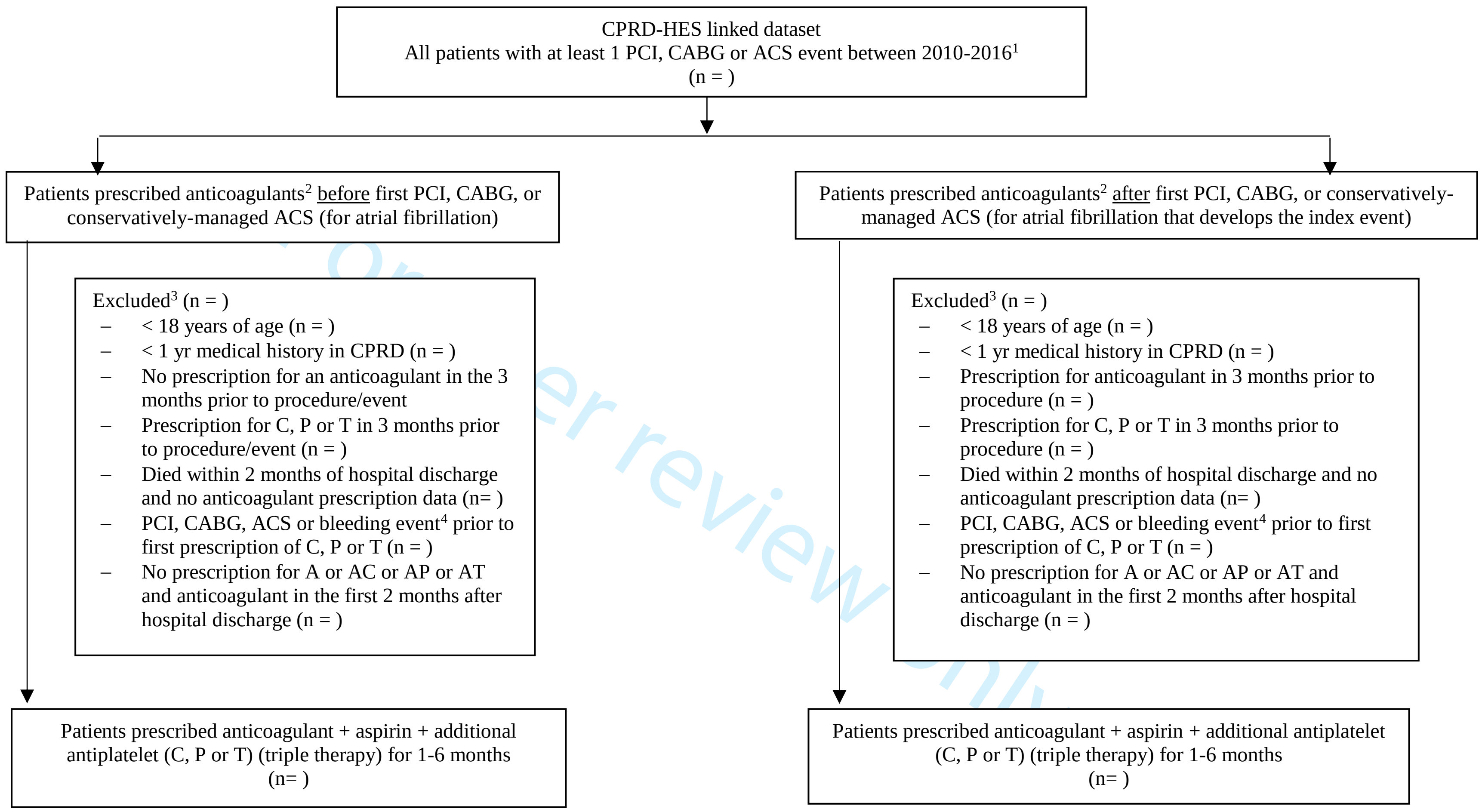

C: clopidogrel; P: prasugrel; T: ticagrelor; AC: aspirin + clopidogrel; AP: aspirin + prasugrel; AT: aspirin + ticagrelor
ACS: acute coronary syndrome; CABG: coronary artery bypass grafting; CPRD, Clinical Practice Research Datalink; HES; Hospital Episode Statistics; ICD International Classification of Diseases; OPCS, Office of Population Censuses and Surveys; PCI: percutaneous coronary intervention.
[1]Continuous inpatient stays (CIPS) that include both PCI and CABG are considered CABG; CIPS that include both ACS and PCI are considered PCI; CIPS that include both ACS and CABG are considered CABG. [2]Warfarin, dabigatran, rivaroxaban, apixaban. [3]Patients may be excluded for more than one reason. [4]Bleeding event captured within hospital admission or GP appointment.

For peer review only

Appendix 1: List of Read codes and ICD-10 diagnosis codes for bleeding in Clinical Practice Research Datalink (CPRD) and Hospital Episode Statistics (HES)

Read Code	Read Term
J573011	Rectal bleeding
R047.00	[D]Epistaxis
158..12	Vaginal bleeding
K5A1.00	Postmenopausal bleeding
K59z.11	Break - through bleeding
1C6..11	Epistaxis symptom
J573.11	Bleeding PR
F4C7100	Subconjunctival haemorrhage
K597.00	Postcoital bleeding
16B3.00	Spontaneous bruising
1C6..00	Nose bleed symptom
K59y300	Intermenstrual bleeding
SE4..11	Leg bruise
K596.11	Intermenstrual bleeding - irregular
K59yx11	Dysfunctional uterine bleeding
196C.00	Painless rectal bleeding
SE4z.11	Haematoma NOS
J573000	Rectal haemorrhage
SE...11	Haematoma with intact skin
..00	Bruising symptom
SE3..11	Arm bruise
22E9.00	O/E-subconjunctival haemorrh'g
K59yx00	Dysfunctional uterine haemorrhage NOS
G60..00	Subarachnoid haemorrhage
F4C7200	Conjunctival haemorrhage NOS
G848000	Bleeding haemorrhoids NOS
R047.11	[D]Nosebleed
SE33011	Subungal haematoma
R027.11	[D]Spontaneous bruising
K596.00	Metrorrhagia
2I15.00	O/E - bruising
J68z.11	GIB - Gastrointestinal bleeding
196B.00	Painful rectal bleeding
J68..00	Gastrointestinal haemorrhage
SE23111	Perianal haematoma
1C62.00	Has nose bleeds - epistaxis
G8y0.00	Haemorrhage NOS
K5E..00	Other abnormal uterine and vaginal bleeding
SE10.00	Black eye NOS
K56y111	Bleeding - vaginal NOS

For peer review only

G61..00	Intracerebral haemorrhage
F42y500	Retinal haemorrhage NOS
F4K2800	Vitreous haemorrhage
J573.00	Haemorrhage of rectum and anus
G844.11	Perianal haematoma
2D85.00	O/E - blood in auditory canal
SE46.00	Traumatic haematoma
2BB8.00	O/E - vitreous haemorrhages
J68zz00	Gastrointestinal tract haemorrhage NOS
1C6Z.00	Nose bleed symptom NOS
SP21300	Primary post tonsillectomy haemorrhage
S622.00	Closed traumatic subdural haemorrhage
K575.00	Haematoma of vulva
K19y411	Urethral bleeding
K31y000	Breast haematoma due to nontraumatic cause
S62..13	Subdural haemorrhage following injury
7032000	Evacuation of extradural haematoma
SP21400	Secondary post tonsillectomy haemorrhage
K19y400	Bleeding from urethra
J68z000	Gastric haemorrhage NOS
J150000	Acute haemorrhagic gastritis
J510900	Bleeding diverticulosis
R09z000	[D]Umbilical bleeding
J68z100	Intestinal haemorrhage NOS
G60z.00	Subarachnoid haemorrhage NOS
K5E1.00	Abnormal uterine bleeding, unspecified
S62A.00	Traumatic extradural haematoma
J573100	Anal haemorrhage
F404500	Intra-ocular haemorrhage
SP21.12	Haemorrhage - postoperative
J120100	Acute duodenal ulcer with haemorrhage
S62..00	Cerebral haemorrhage following injury
SE22300	Haematoma of rectus sheath
J121111	Bleeding chronic duodenal ulcer
G62z.00	Intracranial haemorrhage NOS
G617.00	Intracerebral haemorrhage, intraventricular
J110111	Bleeding acute gastric ulcer
J68z.00	Gastrointestinal haemorrhage unspecified
K5C2.00	Haematocolpos
F503100	Haematoma of pinna
K286100	Scrotal haemorrhage
SP03216	Bleeding due to intrauterine contraceptive device
SE42011	Heel bruise
7M0G000	Aspiration of haematoma of organ NOC

For peer review only

K286v00	Male genital haematoma NOS
K566.00	Vaginal haematoma
SP21200	Post-operative haematoma formation
G602.00	Subarachnoid haemorrhage from middle cerebral artery
S62..11	Extradural haemorrhage following injury
F42y400	Subretinal haemorrhage
7004300	Evacuation of intracerebral haematoma NEC
G77z000	Capillary haemorrhage
SE06.00	Bruise of mandibular joint area
K544.00	Haematometra
S760100	Kidney haematoma without mention of open wound into cavity
K138300	Intrarenal haematoma
K59yy00	Functional uterine haemorrhage NOS
S760111	Renal haematoma without mention of open wound into cavity
7M0U400	Reexploration of organ & surgical arrest postop bleeding NOC
J111100	Chronic gastric ulcer with haemorrhage
7004200	Evacuation of haematoma from cerebellum
SP03217	Contraception IUCD causing bleeding
K275100	Corpus cavernosum haematoma
K286400	Testicular haemorrhage
ZA13700	Drainage of subungual haematoma with hot wire
S628.00	Traumatic subdural haemorrhage
7008200	Aspiration of haematoma of brain tissue
SE11.12	Bruise of periocular tissue
7H02200	Reopen chest reexplore intraabdom op site surg arr PO bleed
S624.00	Closed traumatic extradural haemorrhage
F4G3200	Exophthalmos due to orbital haemorrhage
S740100	Liver haematoma and contusion without open wound into cavity
S620.00	Closed traumatic subarachnoid haemorrhage
TA0..11	Accidental haemorrhage during medical care
K221100	Prostatic haemorrhage
SP21000	Intra-operative haemorrhage
S626.00	Epidural haemorrhage
7404z00	Surgical arrest of bleeding from internal nose NOS
C063000	Thyroid haemorrhage
ZA13800	Drainage of subungual haematoma with drill
Kyu9D00	[X]Other specified abnormal uterine and vaginal bleeding
H5y0000	Tracheostomy haemorrhage
7E0F500	Uterus operation haemostasis
7H22600	Reopen abdo reexplore intraabd op site surg arr postop bleed
F436z00	Choroidal haemorrhage or rupture NOS
Gyu6200	[X]Other intracerebral haemorrhage
F501G00	Haemorrhagic otitis externa
D305000	Haemorrhagic disorder due to antithrombinaemia

For peer review only

J11yy00	Unspec gastric ulcer; unspec haemorrhage and/or perforation
J13y100	Unspecified peptic ulcer with haemorrhage
D305.00	Haemorrhagic disorder due to circulating anticoagulants
SK02.11	Secondary and recurrent haemorrhage
7404y00	Surgical arrest of bleeding from internal nose OS
J111300	Chronic gastric ulcer with haemorrhage and perforation
K138100	Renal artery haemorrhage
C154200	Adrenal haemorrhage
F42y300	Deep retinal haemorrhage
J12yy00	Unspec duodenal ulcer; unspec haemorrhage and/or perforation
J130300	Acute peptic ulcer with haemorrhage and perforation
K167.00	Haemorrhage into bladder wall
7609y11	Tanner devascularisation for bleeding varices
2DE7.00	O/E - throat haemorrhage
J14y100	Unspecified gastrojejunal ulcer with haemorrhage
Gyu6100	[X]Other subarachnoid haemorrhage
F437200	Haemorrhagic choroidal detachment
7303200	Drainage haematoma ext ear control cavity c bolster suture
Ryu7300	[X]Haemorrhage, not elsewhere classified
J13y300	Unspecified peptic ulcer with haemorrhage and perforation
Gyu6F00	[X]Intracerebral haemorrhage in hemisphere, unspecified
G852000	Oesophageal varices with bleeding in diseases EC
SE45.11	Haematoma of leg
1584	Heavy episode of vaginal bleeding
7017000	Evacuation of subdural haematoma
SP21.11	Haematoma - postoperative
J573012	PRB - Rectal bleeding
SE30011	Shoulder bruise
2D25.00	O/E - epistaxis
SE1..11	Bruise of eye
F42y.11	Haemorrhage - retinal
K5Ez.00	Abnormal uterine and vaginal bleeding, unspecified
7736000	Evacuation of perianal haematoma Subdural haemorrhage - nontraumatic
G621.00 K286000	Scrotal haematoma due to nontraumatic cause
K56y100	Haemorrhage of vagina
K56y112	BPV - Vaginal bleeding
SP21.00	Peri-operative haemorrhage or haematoma
K286w00	Male genital haemorrhage NOS
G61z.00	Intracerebral haemorrhage NOS
SE0..11	Bruise of face
16B2.00	Bruises easily
G622.00	Subdural haematoma - nontraumatic
SP21100	Post-operative haemorrhage

For peer review only

K59y.11	Metropathia haemorrhagica
S629.00	Traumatic subdural haematoma
2556	O/E - bleeding gums
J68z200	Upper gastrointestinal haemorrhage
F586200	Otorrhagia
G61..12	Stroke due to intracerebral haemorrhage
G842000	Internal bleeding haemorrhoids
G530.00	Haemopericardium
SK02.12	Secondary and recurrent haemorrhage
K587.00	Contact bleeding of cervix
G850.00	Oesophageal varices with bleeding
G61..11	CVA - cerebrovascular acid due to intracerebral haemorrhage
1928	Bleeding gums
2BB5.00	O/E - retinal haemorrhages
K5E2.00	Abnormal vaginal bleeding, unspecified
SE0..12	Bruise of head
7M0G400	Evacuation of haematoma NEC
SE43.11	Toenail bruise
SE2..11	Bruise, trunk
16BZ.00	Bruising symptom NOS
K599.00	Mid-cycle bleeding
16B4.00	Post-traumatic bruising
G623.00	Subdural haemorrhage NOS
R063000	[D]Cough with haemorrhage
G613.00	Cerebellar haemorrhage
G845000	External bleeding haemorrhoids
J12y100	Unspecified duodenal ulcer with haemorrhage
J573z00	Haemorrhage of rectum and anus NOS
G718.00	Leaking abdominal aortic aneurysm
SE24211	Bruise of scrotum
J110100	Acute gastric ulcer with haemorrhage
2D66.00	O/E - blood from ear
7303000	Drainage of haematoma of external ear
25T0.00	Bleeding stoma
R063100	[D]Pulmonary haemorrhage NOS
F436000	Unspecified choroidal haemorrhage
J10y000	Haemorrhage of oesophagus
G360.00	Haemopericardium/current comp folow acute myocardial infarction
SK02.00	Secondary and recurrent haemorrhage
J121100	Chronic duodenal ulcer with haemorrhage
ZA13600	Drainage of subungual haematoma
K286300	Testicular haematoma due to nontraumatic cause
SE05.11	Bruise of ear
7D05200	Evacuation of haematoma of vulva

For peer review only

J56y000	Haemoperitoneum - nontraumatic
G614.00	Pontine haemorrhage
K5E0.00	Abnormal uterine bleeding unrelated to menstrual cycle
G611.00	Internal capsule haemorrhage
G60X.00	Subarachnoid haemorrhage from intracranial artery, unspecified
K13y800	Perirenal haematoma
7004100	Evacuation of haematoma from temporal lobe of brain
K16y200	Bladder haemorrhage
K221.00	Prostatic congestion or haemorrhage
G61X000	Left sided intracerebral haemorrhage, unspecified
66UI.00	Hormone replacement therapy bleed pattern - abnormal
G620.00	Extradural haemorrhage - nontraumatic
851..00	Haemorrhage control by packing
F4Ey000	Haemorrhage of eyelid
G61X100	Right sided intracerebral haemorrhage, unspecified
7D1C000	Evacuation of haematoma from vagina
SE11.11	Bruise of eyelids
R048. 00	[D]Throat haemorrhage
7404	Surgical arrest of bleeding from internal nose
S750100	Spleen haematoma without mention of open wound into cavity
F436.00	Choroidal haemorrhage and rupture
SE4z.12	Intramuscular haematoma NOS
F4K7.00	Retrobulbar haemorrhage
G603.00	Subarachnoid haemorrhage from anterior communicating artery
S62..12	Subarachnoid haemorrhage following injury
G610.00	Cortical haemorrhage
G62..00	Other and unspecified intracranial haemorrhage
662o.00	Haemorrhagic stroke monitoring
S630.12	Intracranial haematoma following injury
J130100	Acute peptic ulcer with haemorrhage
K55y300	Haemorrhage of cervix
G61X.00	Intracerebral haemorrhage in hemisphere, unspecified
K53y600	Haematosalpinx
J111111	Bleeding chronic gastric ulcer
G605.00	Subarachnoid haemorrhage from basilar artery
2D65.00	O/E - blood stained ear disch.
G612.00	Basal nucleus haemorrhage
F42y100	Superficial retinal haemorrhage
G604.00	Subarachnoid haemorrhage from posterior communicating artery
K59B.00	Postmenopausal postcoital bleeding
761D500	Endoscopic injection haemostasis of duodenal ulcer
S62z.00	Cerebral haemorrhage following injury NOS
K537.00	Haematoma of the broad ligament
7405300	Insertion of Brighton epistaxis balloon

For peer review only

F4H4100	Optic nerve sheath haemorrhage
S624.11	Epidural haematoma following injury
7619100	Gastrotomy and ligation of bleeding point of stomach
K59A.00	Premenopausal postcoital bleeding
S62..14	Traumatic cerebral haemorrhage
S627.00	Traumatic subarachnoid haemorrhage
7G2H400	Liposuction removal of haematoma
J131100	Chronic peptic ulcer with haemorrhage
FyuH400	[X]Vitreous haemorrhage in diseases classified elsewhere
J11y100	Unspecified gastric ulcer with haemorrhage
J120300	Acute duodenal ulcer with haemorrhage and perforation
S629000	Traumatic subdural haematoma without open intracranial wound
G618.00	Intracerebral haemorrhage, multiple localized
F424300	Retinal pigment epithelium haemorrhagic detachment
F436100	Expulsive choroidal haemorrhage
G616.00	External capsule haemorrhage
G615.00	Bulbar haemorrhage
K275200	Corpus cavernosum haemorrhage
761D600	Endoscopic injection haemostasis of gastric ulcer
J017200	Teeth staining due to pulpal bleeding
7L1L300	Haemostasis of unspecified organ
SE05.12	Bruise of auricle
S761100	Kidney haematoma with open wound into cavity
K221z00	Prostatic congestion or haemorrhage NOS
S625.00	Open traumatic extradural haemorrhage
S63..00	Other cerebral haemorrhage following injury
G606.00	Subarachnoid haemorrhage from vertebral artery
7B37400	Open haemostasis of prostate
S63z.00	Other cerebral haemorrhage following injury NOS
7J01300	Reopen cranium reexploration op site arrest post op bleeding
G601.00	Subarachnoid haemorrhage from carotid siphon and bifurcation
D305100	Haemorrhagic disorder due to hyperheparinaemia
J121300	Chronic duodenal ulcer with haemorrhage and perforation
S751100	Spleen haematoma with open wound into cavity
C12y100	Haemorrhage of parathyroid
J110300	Acute gastric ulcer with haemorrhage and perforation
J12y300	Unspecified duodenal ulcer with haemorrhage and perforation
S623.00	Open traumatic subdural haemorrhage
J140100	Acute gastrojejunal ulcer with haemorrhage
S621.00	Open traumatic subarachnoid haemorrhage

For peer review only

ICD Code	ICD Term	Type
I85.0	Oesophageal varices with bleeding	gastrointestinal
K25.0	Gastric ulcer, acute with haemorrhage	gastrointestinal
K25.2	Gastric ulcer, acute with both haemorrhage and perforation	gastrointestinal
K25.4	Gastric ulcer, chronic or unspecified with haemorrhage	gastrointestinal
K25.6	Chronic or unspecified with both haemorrhage and perforation	gastrointestinal
K26.0	Duodenal ulcer, acute with haemorrhage	gastrointestinal
K26.2	Duodenal ulcer, acute with both haemorrhage and perforation	gastrointestinal
K26.4	Duodenal ulcer, chronic or unspecified with haemorrhage	gastrointestinal
K26.6	Chronic or unspecified with both haemorrhage and perforation	gastrointestinal
K27.0	Peptic ulcer, acute with haemorrhage	gastrointestinal
K27.2	Peptic ulcer, acute with both haemorrhage and perforation	gastrointestinal
K27.4	Peptic ulcer, chronic or unspecified with haemorrhage	gastrointestinal
K27.6	Chronic or unspecified with both haemorrhage and perforation	gastrointestinal
K28.0	Gastrojejunal ulcer, acute with haemorrhage	gastrointestinal
K28.2	Acute with both haemorrhage and perforation	gastrointestinal
K28.4	Gastrojejunal ulcer, chronic or unspecified with haemorrhage	gastrointestinal
K28.6	Chronic or unspecified with both haemorrhage and perforation	gastrointestinal
K29.0	Acute haemorrhagic gastritis	gastrointestinal
K62.5	Haemorrhage of anus and rectum	gastrointestinal
K66.1	Haemoperitoneum	gastrointestinal
K92.0	Haematemesis	gastrointestinal
K92.1	Melaena	gastrointestinal
K92.2	Gastrointestinal haemorrhage, unspecified	gastrointestinal
I60	Subarachnoid haemorrhage	intracranial bleed
I60.0	Subarachnoid haemorrhage from carotid siphon and bifurcation	intracranial bleed
I60.1	Subarachnoid haemorrhage from middle cerebral artery	intracranial bleed
I60.2	Subarachnoid haemorrhage from anterior communicating artery	intracranial bleed
I60.3	Subarachnoid haemorrhage from posterior communicating artery	intracranial bleed
I60.4	Subarachnoid haemorrhage from basilar artery	intracranial bleed
I60.5	Subarachnoid haemorrhage from vertebral artery	intracranial bleed
I60.6	Subarachnoid haemorrhage from other intracranial arteries	intracranial bleed
I60.7	Subarachnoid haemorrhage from intracranial artery, unspec	intracranial bleed
I60.8	Other subarachnoid haemorrhage	intracranial bleed
I60.9	Subarachnoid haemorrhage, unspecified	intracranial bleed
I61	Intracerebral haemorrhage	intracranial bleed

For peer review only

I61.0	Intracerebral haemorrhage in hemisphere, subcortical	intracranial bleed
I61.1	Intracerebral haemorrhage in hemisphere, cortical	intracranial bleed
I61.2	Intracerebral haemorrhage in hemisphere, unspecified	intracranial bleed
I61.3	Intracerebral haemorrhage in brain stem	intracranial bleed
I61.4	Intracerebral haemorrhage in cerebellum	intracranial bleed
I61.5	Intracerebral haemorrhage, intraventricular	intracranial bleed
I61.6	Intracerebral haemorrhage, multiple localized	intracranial bleed
I61.8	Other intracerebral haemorrhage	intracranial bleed
I61.9	Intracerebral haemorrhage, unspecified	intracranial bleed
I62	Other nontraumatic intracranial haemorrhage	intracranial bleed
I62.0	Subdural haemorrhage (acute)(nontraumatic)	intracranial bleed
I62.1	Nontraumatic extradural haemorrhage	intracranial bleed
I62.9	Intracranial haemorrhage (nontraumatic), unspecified	intracranial bleed
I69.0	Sequelae of subarachnoid haemorrhage	intracranial bleed
I69.1	Sequelae of intracerebral haemorrhage	intracranial bleed
I69.2	Sequelae of other nontraumatic intracranial haemorrhage	intracranial bleed
S06.4	Epidural haemorrhage	intracranial bleed

For peer review only

Appendix 2: ADAPTT literature searches

DATABASE	Set 1. POPULATION + INTERVENTION + STUDIES *(RCTs/Cohort - **Dual/Triple antiplatelet therapy**)*	Set 2. POPULATION + INTERVENTION + OUTCOME *(bleeding)*	Set 3. POPULATION + OUTCOME + RISK *Risk of bleeding after a coronary intervention*
**CENTRAL** (database of controlled studies: RCTs, CCTs, ITS, CBA)	**775**	Included in Set 1 (CENTRAL database)	**720**
**MEDLINE**	**1822**	**558** (de-duplicated against Set 1 MEDLINE)	**5001**
**Embase**	**1156**	**520** (de-duplicated against Set 1 EMBASE)	**1582**
Total=	**3753**	**1078**	**7303**
After de-duplication=	**2544**	**849**	**6273**

**Set 3 (P-O-R)**	**Search-within-a-search-1**:<br>(score or scores or model or models or tool or tools or algorithm* or prognosis or predict or prediction or cohort):ti,ab n=1843
	**Search-within-a-search-2:**<br>(risk near3 (score* or factor or factors or model or models or prediction or stratification or category or bleed*)):ti,ab n=3300 (prior to de-duplication)

1. OVID MEDLINE

[Epub Ahead of Print, In-Process & Other Non-Indexed Citations, Ovid MEDLINE(R) Daily and Ovid MEDLINE(R) 1946 to Present]

1. Acute Coronary Syndrome/

2. (acute coronary adj3 syndrome*).ti,ab,kf.

3. ACS.ti,ab,kf.

4. heart attack*1.ti,ab,kf.

5. exp Myocardial Infarction/

6. myocardial infarct*.ti,ab,kf.

7. (MI or AMI).ti,ab,kf.

8. (stemi or non-stemi or nstemi).ti,ab,kf.

9. exp Angina, Unstable/

10. (angina adj3 unstable).ti,ab,kf.

11. exp Percutaneous Coronary Intervention/

12. (percutaneous coronary adj3 intervention).ti,ab,kf.

13. (PCI or PPCI or PCI-S).ti,ab,kf.

14. exp Angioplasty/

15. angioplasty.ti,ab,kf.

16. exp Stents/

17. stent*1.ti,ab,kf.

18. exp Coronary Artery Bypass/

19. CABG.ti,ab,kf.

20. coronary artery bypass.ti,ab,kf.

21. or/1-20 [Population]

22. (dual antiplatelet adj (therapy or treatment)).ti,ab,kf.

23. (DAPT or DAT).ti,ab,kf.

24. or/22-23

25. Aspirin/

26. (aspirin or acetylsalicylic acid or ASA).ti,ab,kf,rn,nm.

27. or/25-26

28. (clopidrogel or prasugrel or ticagrelor or plavix or efient or brilinta).ti,ab,kf,rn,nm,sh.

29. PURINERGIC P2Y RECEPTOR ANTAGONISTS/

30. (P2Y12 adj2 (antagonist* or inhibitor*)).ti,ab,kf,rn,nm.

31. or/28-30

32. 24 or (27 and 31)

33. exp Anticoagulants/

34. (anticoagul* or antithrombo* or anti-coagul* or anti-thrombo* or OAC* or DOAC* or NOAC*).ti,ab,kf,rn,nm.

35. (coumarin* or coumadin* or warfarin or marevan or dicoumarol or dicoumarin or dicumarin or dicumarol or acenocoumarol or phenindione or aldocumar or dabigatram or pradaxa or BIBR1048 or Apixaban or Eliquis or BMS-562247-01 or Edoxaban or Lixiana or

For peer review only

savaysa or DU-176b or betrixaban or PRT-054021 or PRT0504021 or rivaroxaban or xarelto or BAY-59739 or Erixaban or D0913).ti,ab,kf,rn,nm.

36. ((Vitamin K or Factor Xa or Factor 10a or Factor IIa) adj2 (antagonist* or inhibitor*)).ti,ab,kw,rn,nm.

37. or/33-36

38. (triple therapy or triple antiplatelet therapy or triple antithrombotic therapy or triple antithrombotic combination therapy).ti,ab,kf.

39. (TAPT or TOAT).ti,ab,kf.

40. ((24 or 31) and 37) or 38 or 39

41. 32 or 40 [Intervention]

42. (bleed*1 or bleeding).ti,ab,kf.

43. Hemorrhage/

44. (hemorrhag* or haemorrhag*).ti,ab,kf.

45. or/42-44 [Outcome]

46. risk/ or risk assessment/ or risk factors/

47. risk stratification.ti,ab,kf.

48. (risk adj3 model*).ti,ab,kf.

49. risk factor*.ti,ab,kf.

50. or/46-49 [Risk]

51. randomized controlled trial.pt.

52. controlled clinical trial.pt.

53. (RCT or randomi*).ti,ab,kf.

54. placebo.ab.

55. (random* adj (assign* or allocat* or divide* or division)).ti,ab,kf.

56. trial.ti,ab.

57. groups.ab.

58. or/51-57

59. cohort studies/ or follow-up studies/ or longitudinal studies/ or prospective studies/ or retrospective studies/

60. longitudinal.ab.

61. (prospective or retrospective).ab.

62. (CCT or (control* adj (trial*1 or study or studies))).ti,ab,kf.

For peer review only

63. (Follow up adj2 (study or studies)).ti,ab,kf.

64. follow up assessment.ti,ab,kf.

65. (compar* and group*).ab.

66. cohort.ti,ab,kf.

67. (register or registry).ti,ab,kf.

68. or/59-67

69. 58 or 68 **[Study Design Filter]**

70. 21 and 41 and 69

71. 21 and 41 and 45

72. 21 and 41 and 50

73. 21 and 41 and 45 and 50

74. 21 and 45 and 50

For peer review only

2. The Cochrane Library, Issue 7, 2016

ID	Search
#1	MeSH descriptor: [Acute Coronary Syndrome] explode all trees
#2	"acute coronary syndrome":ti,ab,kw (Word variations have been searched)
#3	ACS:ab (Word variations have been searched)
#4	heart attack*:ti,ab,kw (Word variations have been searched)
#5	MeSH descriptor: [Myocardial Infarction] explode all trees
#6	myocardial next infarct*:ti,ab,kw (Word variations have been searched)
#7	MI or AMI:ab (Word variations have been searched)
#8	(stemi or non-stemi or nstemi):ti,ab,kw (Word variations have been searched)
#9	MeSH descriptor: [Angina, Unstable] explode all trees
#10	(angina near unstable):ti,ab,kw (Word variations have been searched)
#11	MeSH descriptor: [Percutaneous Coronary Intervention] explode all trees
#12	(percutaneous next coronary) and intervention:ti,ab,kw (Word variations have been searched)
#13	PCI or PPCI or PCI-S:ab (Word variations have been searched)
#14	MeSH descriptor: [Angioplasty] explode all trees
#15	angioplasty:ti,ab,kw (Word variations have been searched)
#16	MeSH descriptor: [Stents] explode all trees

#17 stent or stents or stenting:ti,ab,kw (Word variations have been searched)

#18 MeSH descriptor: [Coronary Artery Bypass] explode all trees

#19 CABG:ab (Word variations have been searched)

#20 "coronary artery bypass":ti,ab,kw (Word variations have been searched)

#21 (#1 or #2 or #3 or #4 or #5 or #6 or #7 or #8 or #9 or #10 or #11 or #12 or #13 or #14 or #15 or #16 or #17 or #18 or #19 or #20)

#22 (dual next antiplatelet) and (therapy or treatment):ti,ab,kw (Word variations have been searched)

#23 (DAPT or DAT):ti,ab,kw (Word variations have been searched)

#24 #22 or #23

#25 MeSH descriptor: [Aspirin] explode all trees

#26 aspirin or "acetylsalicylic acid":ti,ab,kw (Word variations have been searched)

#27 ASA:ab (Word variations have been searched)

#28 #25 or #26 or #27

#29 (clopidrogel or prasugrel or ticagrelor or plavix or efient or brilinta):ti,ab,kw (Word variations have been searched)

#30 MeSH descriptor: [Purinergic P2Y Receptor Antagonists] explode all trees

#31 (P2Y12 near (antagonist* or inhibitor*)):ti,ab,kw (Word variations have been searched)

#32 #29 or #30 or #31

#33 #24 or (#28 and #32)

#34 MeSH descriptor: [Anticoagulants] explode all trees

#35 (anticoagul* or antithrombo* or anti-coagul* or anti-thrombo* or OAC* or DOAC* or NOAC*):ti,ab,kw (Word variations have been searched)

#36 (coumarin* or coumadin* or warfarin or marevan or dicoumarol or dicoumarin or dicumarin or dicumarol or acenocoumarol or phenindione or aldocumar):ti,ab,kw (Word variations have been searched)

#37 dabigatram or pradaxa or BIBR1048 or Apixaban or Eliquis or BMS-562247-01 or Edoxaban or Lixiana or savaysa or DU-176b or betrixaban or PRT-054021 or PRT0504021 or rivaroxaban or xarelto or BAY-59739 or Erixaban or D0913:ti,ab,kw (Word variations have been searched)

#38 "vitamin K" and (antagonist* or inhibitor*):ti,ab,kw (Word variations have been searched)

#39 ("vitamin K" or "factor Xa" or "factor 10a" or "factor IIa") and (antagonist* or inhibitor*):ti,ab,kw (Word variations have been searched)

#40 #34 or #35 or #36 or #37 or #38 or #39

#41 triple near therapy:ti,ab,kw (Word variations have been searched)

For peer review only

#42 TAPT or TOAT:ab (Word variations have been searched)

#43 ((#24 or #32) and #40) or #41 or #42

#44 #33 or #43

#45 MeSH descriptor: [Hemorrhage] explode all trees

#46 bleed*:ti,ab,kw (Word variations have been searched)

#47 hemorrhag* or haemorrhag*:ti,ab,kw (Word variations have been searched)

#48 #45 or #46 or #47

#49 MeSH descriptor: [Risk] explode all trees

#50 "risk stratification":ti,ab,kw (Word variations have been searched)

#51 risk near (factor* or model*):ti,ab,kw (Word variations have been searched)

#52 #49 or #50 or #51

#53 #21 and #44

#54 #21 and #48 and #52

#55 (#54 not #53)

3. OVID EMBASE (1974 to date)

1. exp acute coronary syndrome/
2. (acute coronary adj3 syndrome*).ti,ab,kw.
3. ACS.ti,ab,kw.
4. heart attack*1.ti,ab,kw.
5. exp heart infarction/
6. myocardial infarct*.ti,ab,kw.
7. (MI or AMI).ti,ab,kw.
8. (stemi or non-stemi or nstemi).ti,ab,kw.
9. exp unstable angina pectoris/
10. (angina adj3 unstable).ti,ab,kw.
11. exp percutaneous coronary intervention/
12. (percutaneous coronary adj3 intervention).ti,ab,kw.
13. (PCI or PPCI or PCI-S).ti,ab,kw.
14. exp angioplasty/
15. angioplasty.ti,ab,kw.
16. exp stent/
17. stent*1.ti,ab,kw.

For peer review only

18. coronary artery bypass graft/

19. CABG.ti,ab,kw.

20. coronary artery bypass.ti,ab,kw.

21. or/1-20

22. (dual antiplatelet adj (therapy or treatment)).ti,ab,kw.

23. (DAPT or DAT).ti,ab,kw.

24. or/22-23

25. acetylsalicylic acid/

26. (aspirin or acetylsalicylic acid or ASA).ti,ab,kw,rn,tn.

27. or/25-26

28. (clopidrogel or prasugrel or ticagrelor or plavix or efient or brilinta).ti,ab,kw,rn,tn,sh.

29. antithrombocytic agent/

30. (P2Y12 adj2 (antagonist* or inhibitor*)).ti,ab,kw,rn.

31. exp purinergic receptor blocking agent/

32. or/28-31

33. 24 or (27 and 32)

34. exp anticoagulant agent/

35. (anticoagul* or antithrombo* or anti-coagul* or anti-thrombo* or OAC* or DOAC* or NOAC*).ti,ab,kw.

36. (coumarin* or coumadin* or warfarin or marevan or dicoumarol or dicoumarin or dicumarin or dicumarol or acenocoumarol or phenindione or aldocumar).ti,ab,kw,rn,tn.

37. (dabigatram or pradaxa or BIBR1048 or Apixaban or Eliquis or BMS-562247-01 or Edoxaban or Lixiana or savaysa or DU-176b or betrixaban or PRT-054021 or PRT0504021 or rivaroxaban or xarelto or BAY-59739 or Erixaban or D0913).ti,ab,kw,rn,tn.

38. (vitamin K adj2 (antagonist$ or inhibitor$)).ti,ab,kw,rn.

39. (factor Xa adj2 (antagonist$ or inhibitor$)).ti,ab,kw,rn.

40. (factor 10a adj2 (antagonist$ or inhibitor$)).ti,ab,kw,rn.

41. (factor IIa adj2 (antagonist$ or inhibitor$)).ti,ab,kw,rn.

42. (adjunct* or combin* or concurrent or cotherap* or co-therap* or dual or plus or triple).ti,ab,kw.

43. drug combination/

44. or/34-43

45. (triple therapy or triple antiplatelet therapy or triple antithrombotic therapy or triple antithrombotic combination therapy).ti,ab,kw.

46. (TAPT or TOAT).ti,ab,kw.

For peer review only

47. ((24 or 32) and 44) or 45 or 46

48. 33 or 47

49. (bleed*1 or bleeding).ti,ab,kw.

50. exp Bleeding/

51. (hemorrhag* or haemorrhag*).ti,ab,kw.

52. or/49-51

53. risk assessment/ or risk factor/ or patient risk/ or risk/ or high risk patient/

54. risk stratification.ti,ab,kw.

55. (risk adj3 model*).ti,ab,kw.

56. risk factor*.ti,ab,kw.

57. or/53-56

58. Randomized Controlled Trial/

59. Randomization/

60. (random* adj (assign* or allocat* or divide* or division)).ti,ab,kw.

61. (RCT or randomi*).ti,ab,kw.

62. trial.ti,ab.

63. placebo.ti,ab,kw.

64. ((assign$ or match or matched or allocation) adj5 (alternate or group$1 or intervention$1 or patient$1 or subject$1 or participant$1)).ti,ab,kw.

65. double blind procedure/

66. ((double or single or doubly or singly) adj (blind or blinded or blindly)).ti.

67. or/58-66

68. Controlled Clinical Trial/

69. (CCT or (controlled adj7 (study or design or trial))).ti,ab,kw.

70. cohort analysis/

71. cohort.ti,ab,kw.

72. longitudinal.ab.

73. (prospective or retrospective).ab.

74. follow up assessment.ti,ab,kw.

75. clinical trial/ or multicenter study/ or phase 2 clinical trial/ or phase 3 clinical trial/ or phase 4 clinical trial/

76. clinical study/ or exp longitudinal study/ or major clinical study/ or prospective study/ or retrospective study/

77. (Follow up adj2 study).ti,ab,kw.

For peer review only

78. register.ti,ab,kw.

79. or/68-78

80. 67 or 79

81. Animal experiment/ not (human experiment/ or human/)

82. (rat or rats or mouse or mice or swine or porcine or murine or sheep or lambs or pigs or piglets or rabbit or rabbits or cat or cats or dog or dogs or cattle or bovine or monkey or monkeys or trout or marmoset$1).ti. and animal experiment/

83. or/81-82

84. 80 not 83

85. 21 and 48 and 84

86. limit 85 to exclude medline journals

87. **21 and 48 and 52**

88. limit 87 to exclude medline journals

89. **21 and 48 and 57**

90. limit 89 to exclude medline journals

91. **21 and 48 and 52 and 57**

92. 86 or 88 or 90 or 91

For peer review only

Appendix 3: List of product codes in Clinical Practice Research Datalink (CPRD). All for antiplatelet agents (aspirin, clopidogrel, prasugrel and ticagrelor) and anticoagulants (warfarin, dabigatran, rivaroxaban, apixaban)

Antiplatelet agents (aspirin, clopidogrel, prasugrel and ticagrelor)

Gemscript product code	Product name	Category
3	Aspirin 75mg dispersible tablets	Aspirin
16	Aspirin 75mg tablets	Aspirin
34	Aspirin 75mg gastro-resistant tablets	Aspirin
111	ASPIRIN 40 MG CAP	Aspirin
216	ASPIRIN 70 MG TAB	Aspirin
254	Aspirin 300mg tablets	Aspirin
377	Aspirin 300mg dispersible tablets	Aspirin
383	ASPIRIN 60 MG TAB	Aspirin
393	Disprin 300mg dispersible tablets (Reckitt Benckiser Healthcare (UK) Ltd)	Aspirin
395	Aspirin mixture	Aspirin
434	Aspirin 300mg gastro-resistant tablets	Aspirin
1137	Nu-seals aspirin ec 300mg Gastro-resistant tablet (Eli Lilly and Company Ltd)	Aspirin
1486	ASPIRIN 75 MG SUP	Aspirin
2105	Solprin 300mg Tablet (Reckitt Benckiser Healthcare (UK) Ltd)	Aspirin
2607	Paynocil Tablet (Beecham Research Laboratories)	Aspirin
2628	Nu-seals aspirin ec 75mg Gastro-resistant tablet (Eli Lilly and Company Ltd)	Aspirin
2754	ASPIRIN SOLUBLE 150 MG TAB	Aspirin
2924	ASPIRIN 150 MG TAB	Aspirin
4271	ASPIRIN SOLUBLE 200 MG TAB	Aspirin
4523	ASPIRIN 50 MG CAP	Aspirin
6006	Nu-Seals 75 gastro-resistant tablets (Alliance Pharmaceuticals Ltd)	Aspirin
6007	Nu-Seals 300 gastro-resistant tablets (Alliance Pharmaceuticals Ltd)	Aspirin
6696	Micropirin 75mg gastro-resistant tablets (Dexcel-Pharma Ltd)	Aspirin
7417	ASPIRIN 40 MG TAB	Aspirin
7462	ASPIRIN 325 MG CAP	Aspirin
7486	ASPIRIN 37.5 MG TAB	Aspirin
7516	Aspirin 300mg effervescent tablets sugar free	Aspirin
7665	ASPIRIN SR 300 MG TAB	Aspirin
7915	ASPIRIN SR 100 MG TAB	Aspirin
7944	ASPIRIN SOLUBLE 40 MG CAP	Aspirin
8185	Disprin CV 300mg modified-release tablets (Reckitt Benckiser Healthcare (UK) Ltd)	Aspirin
8186	Aspirin 300mg modified-release tablets	Aspirin

For peer review only

8424	ASPIRIN PAED 81 MG TAB	Aspirin
8645	Aspirin 300mg effervescent tablets	Aspirin
8733	JUNIOR ASPIRIN 37.5 MG TAB	Aspirin
8734	ASPIRIN disp 37.5 MG TAB	Aspirin
8843	ASPIRIN 325 MG TAB	Aspirin
9027	ASPIRIN disp 150 MG TAB	Aspirin
9144	Caprin 75mg gastro-resistant tablets (Wockhardt UK Ltd)	Aspirin
9301	Aspirin 100mg modified-release tablets	Aspirin
10305	Aspirin 162.5mg capsules	Aspirin
10310	Aspirin powder	Aspirin
11941	ASPIRIN SACHETS 30 MG	Aspirin
11977	Aspro clear maximum strength tablets	Aspirin
12102	ASPIRIN SOLUBLE 100 MG TAB	Aspirin
13882	Imazin XL tablets (Napp Pharmaceuticals Ltd)	Aspirin
15397	ASPIRIN SOLUBLE 50 MG TAB	Aspirin
15517	ASPIRIN 100 MG SUP	Aspirin
17704	Platet 100mg Effervescent tablet (Roche Products Ltd)	Aspirin
17920	Disprin cv 100mg Modified-release tablet (Reckitt Benckiser Healthcare (UK) Ltd)	Aspirin
18030	Imazin XL forte tablets (Napp Pharmaceuticals Ltd)	Aspirin
18217	Aspirin 300mg orodispersible tablets sugar free	Aspirin
18329	Enprin 75mg gastro-resistant tablets (Galpharm International Ltd)	Aspirin
19189	Micropirin 75mg Gastro-resistant tablet (Ratiopharm UK Ltd)	Aspirin
19577	NU-SEALS ASPIRIN	Aspirin
19674	ASPIRIN DISPERSIBLE	Aspirin
19797	NU-SEALS ASPIRIN	Aspirin
19813	ASPIRIN SOLUBLE	Aspirin
20206	ASPIRIN 50 MG SUP	Aspirin
20840	Acetylsalicylic acid mix	Aspirin
21380	Aspirin 75mg / Isosorbide mononitrate 60mg modified-release tablets	Aspirin
21382	Aspirin 150mg / Isosorbide mononitrate 60mg modified-release tablets	Aspirin
21921	Postmi ec 300mg Gastro-resistant tablet (Ashbourne Pharmaceuticals Ltd)	Aspirin
22107	ASPIRIN disp 200 MG TAB	Aspirin
22138	Aspirin 324mg modified-release tablets	Aspirin
22232	Disprin Direct 300mg orodispersible tablets (Reckitt Benckiser Healthcare (UK) Ltd)	Aspirin
22618	Solprin 75mg Tablet (Reckitt Benckiser Healthcare (UK) Ltd)	Aspirin
22864	ASPIRIN PAED MIX	Aspirin
23488	Claradin 300mg Tablet (Nicholas Laboratories Ltd)	Aspirin
23495	ASPIRIN	Aspirin
23593	PostMI 75 dispersible tablets (Ashbourne Pharmaceuticals Ltd)	Aspirin
23878	Nu-seals cardio ec 75mg Gastro-resistant tablet (Genus Pharmaceuticals Ltd)	Aspirin

23932	Aspro Clear 300mg effervescent tablets (Bayer Plc)	Aspirin
24025	Caprin 300mg gastro-resistant tablets (Pinewood Healthcare)	Aspirin
24960	Aspirin 300mg tablets (Vantage)	Aspirin
25335	PostMI 75 EC tablets (Ashbourne Pharmaceuticals Ltd)	Aspirin
25718	Angettes 75 tablets (Bristol-Myers Squibb Pharmaceuticals Ltd)	Aspirin
27467	ASPIRIN SOLUBLE 400 MG TAB	Aspirin
28707	ASPIRIN M/F 324 MG TAB	Aspirin
29515	ACETYLSALICYLIC ACID	Aspirin
29759	Aspro Tablet (Roche Consumer Health)	Aspirin
29848	Aspirin 300mg with Glycine 150mg chewable tablets	Aspirin
30920	Aspirin 300mg Dispersible tablet (M & A Pharmachem Ltd)	Aspirin
31210	Aspirin 300mg Tablet (Co-operative)	Aspirin
31211	Aspirin 75mg Dispersible tablet (A A H Pharmaceuticals Ltd)	Aspirin
31858	Caspac xl 162.5mg Capsule (Pharmacia Ltd)	Aspirin
31870	Aspirin 320mg tablets	Aspirin
31938	Aspirin 75mg gastro-resistant tablets (Sandoz Ltd)	Aspirin
31953	Aspirin 75mg dispersible tablets (IVAX Pharmaceuticals UK Ltd)	Aspirin
31954	Aspirin 75mg dispersible tablets (Teva UK Ltd)	Aspirin
31956	Aspirin 75mg gastro-resistant tablets (Kent Pharmaceuticals Ltd)	Aspirin
32036	Aspirin 75mg dispersible tablets (Actavis UK Ltd)	Aspirin
32210	Aspirin 300mg dispersible tablets (Actavis UK Ltd)	Aspirin
32992	Aspirin 75mg gastro-resistant tablets (Mylan)	Aspirin
33293	Aspirin 75mg gastro-resistant tablets (Sterwin Medicines)	Aspirin
33320	Aspirin 75mg Dispersible tablet (Sovereign Medical Ltd)	Aspirin
33656	Aspirin 75mg dispersible tablets (A A H Pharmaceuticals Ltd)	Aspirin
33662	Aspirin 300mg Dispersible tablet (A A H Pharmaceuticals Ltd)	Aspirin
33668	Aspirin 300mg Dispersible tablet (Rusco Ltd)	Aspirin
33676	Aspirin 75mg dispersible tablets (Kent Pharmaceuticals Ltd)	Aspirin
34309	Aspirin 300mg dispersible tablets (A A H Pharmaceuticals Ltd)	Aspirin
34385	Aspirin 75mg Soluble tablet (Co-operative)	Aspirin
34386	Aspirin 300mg tablets (Actavis UK Ltd)	Aspirin
34434	Aspirin 75mg dispersible tablets (Thornton & Ross Ltd)	Aspirin
34485	Aspirin 75mg gastro-resistant tablets (IVAX Pharmaceuticals UK Ltd)	Aspirin
34611	Aspirin 75mg gastro-resistant tablets (C P Pharmaceuticals Ltd)	Aspirin
34666	Aspirin ec 300mg Gastro-resistant tablet (A A H Pharmaceuticals Ltd)	Aspirin
34762	Aspirin 300mg Gastro-resistant tablet (Galen Ltd)	Aspirin
34796	Aspirin 75mg Gastro-resistant tablet (Galen Ltd)	Aspirin
34797	Aspirin 75mg gastro-resistant tablets (Actavis UK Ltd)	Aspirin
34942	Aspirin 75mg Dispersible tablet (Nucare Plc)	Aspirin
36543	Aspirin 100mg effervescent tablets	Aspirin
37541	Aspirin 227mg medicated chewing-gum	Aspirin
39738	Aspirin 162.5mg modified-release capsules	Aspirin
40144	Aspirin 300mg Dispersible tablet (Thornton & Ross Ltd)	Aspirin

40381	Aspirin 75mg Soluble tablet (C P Pharmaceuticals Ltd)	Aspirin
41512	Aspirin 75mg gastro-resistant tablets (Teva UK Ltd)	Aspirin
41569	Aspirin 300mg tablets (A A H Pharmaceuticals Ltd)	Aspirin
41594	Aspirin 300mg Dispersible tablet (Teva UK Ltd)	Aspirin
42061	ASPIRIN 65 MG SUP	Aspirin
43060	Aspirin 300mg Soluble tablet (Celltech Pharma Europe Ltd)	Aspirin
43434	Aspirin 300mg gastro-resistant tablets (A A H Pharmaceuticals Ltd)	Aspirin
43679	Flamasacard 162.5mg Modified-release capsule (Abbey Pharmaceuticals Ltd)	Aspirin
43709	Aspirin 75mg gastro-resistant tablets (Almus Pharmaceuticals Ltd)	Aspirin
43806	Aspirin 300mg gastro-resistant tablets (Sandoz Ltd)	Aspirin
44639	Aspirin 300mg Dispersible tablet (Nucare Plc)	Aspirin
45643	Aspirin 75mg Soluble tablet (Celltech Pharma Europe Ltd)	Aspirin
45840	Aspirin 300mg Dispersible tablet (Numark Management Ltd)	Aspirin
45851	Aspirin 300mg Soluble tablet (Ranbaxy (UK) Ltd)	Aspirin
47937	Aspirin 75mg dispersible tablets (Wockhardt UK Ltd)	Aspirin
47992	Aspirin 75mg gastro-resistant tablets (A A H Pharmaceuticals Ltd)	Aspirin
48000	Aspirin 300mg tablets (Sigma Pharmaceuticals Plc)	Aspirin
48021	Aspirin 75mg Tablet (Hillcross Pharmaceuticals Ltd)	Aspirin
48165	Aspirin 300mg tablets (Aspar Pharmaceuticals Ltd)	Aspirin
48974	Aspirin 75mg tablets (Phoenix Healthcare Distribution Ltd)	Aspirin
49060	Aspirin 75mg dispersible tablets (Alliance Healthcare (Distribution) Ltd)	Aspirin
49220	Aspirin 300mg tablets (Kent Pharmaceuticals Ltd)	Aspirin
49685	Aspirin 75mg dispersible tablets (Sigma Pharmaceuticals Plc)	Aspirin
50555	Aspirin 300mg dispersible tablets (DE Pharmaceuticals)	Aspirin
50926	Aspirin 75mg dispersible tablets (The Boots Company Plc)	Aspirin
50949	Aspirin 75mg tablets (A A H Pharmaceuticals Ltd)	Aspirin
51561	Aspirin 75mg gastro-resistant tablets (Zanza Laboratories Ltd)	Aspirin
52044	Aspirin 300mg caplets (The Boots Company Plc)	Aspirin
52280	Aspirin 300mg Tablet (Wockhardt UK Ltd)	Aspirin
52618	Aspirin 75mg dispersible tablets (Bristol Laboratories Ltd)	Aspirin
52905	Aspirin 300mg tablets (Lloyds Pharmacy Ltd)	Aspirin
53178	Aspirin 75mg gastro-resistant tablets (Wockhardt UK Ltd)	Aspirin
53622	Aspirin 300mg Tablet (M & A Pharmachem Ltd)	Aspirin
53711	Aspirin 300mg Tablet (Nucare Plc)	Aspirin
53791	Aspirin 150mg suppositories (Alliance Healthcare (Distribution) Ltd)	Aspirin
53804	Aspirin 300mg gastro-resistant tablets (Alliance Healthcare (Distribution) Ltd)	Aspirin
53816	Aspirin 300mg dispersible tablets (Alliance Healthcare (Distribution) Ltd)	Aspirin
54284	Aspirin 75mg dispersible tablets (Almus Pharmaceuticals Ltd)	Aspirin
54430	Aspirin 75mg tablets (Alliance Healthcare (Distribution) Ltd)	Aspirin
54526	Aspirin 300mg tablets (Alliance Healthcare (Distribution) Ltd)	Aspirin

For peer review only

54565	Aspirin 75mg dispersible tablets (Lloyds Pharmacy Ltd)	Aspirin
54734	Aspirin 300mg tablets (Wockhardt UK Ltd)	Aspirin
54997	Aspirin 75mg dispersible tablets (Dowelhurst Ltd)	Aspirin
55230	Aspirin 300mg dispersible tablets (Kent Pharmaceuticals Ltd)	Aspirin
55579	Aspirin 300mg tablets (Almus Pharmaceuticals Ltd)	Aspirin
56007	Aspirin 300mg dispersible tablets (Sigma Pharmaceuticals Plc)	Aspirin
56736	Aspirin 300mg tablets (Waymade Healthcare Plc)	Aspirin
56883	Aspirin 75mg tablets (Waymade Healthcare Plc)	Aspirin
56995	Aspirin 75mg dispersible tablets (Phoenix Healthcare Distribution Ltd)	Aspirin
56996	Aspirin 75mg dispersible tablets (Waymade Healthcare Plc)	Aspirin
57057	Aspirin 75mg dispersible tablets (Wockhardt UK Ltd)	Aspirin
58331	Aspirin 300mg gastro-resistant tablets (Mylan)	Aspirin
59021	Aspirin 75mg gastro-resistant tablets (Bristol Laboratories Ltd)	Aspirin
59244	Aspirin 100mg capsules	Aspirin
59253	Aspirin 75mg gastro-resistant tablets (Waymade Healthcare Plc)	Aspirin
59728	Aspirin 75mg tablets (Alissa Healthcare Research Ltd)	Aspirin
59791	Aspirin 75mg dispersible tablets (Aspar Pharmaceuticals Ltd)	Aspirin
60127	Aspirin 75mg tablets (DE Pharmaceuticals)	Aspirin
60278	Aspirin 300mg tablets (DE Pharmaceuticals)	Aspirin
60693	Aspirin 15mg/5ml oral solution	Aspirin
60694	Aspirin 25mg/5ml oral solution	Aspirin
60777	Aspirin 75mg gastro-resistant tablets (DE Pharmaceuticals)	Aspirin
62334	Aspirin 300mg caplets (Wockhardt UK Ltd)	Aspirin
62430	Aspirin 300mg suppositories (A A H Pharmaceuticals Ltd)	Aspirin
63603	Laboprin Tablet (Laboratories For Applied Biology Ltd)	Aspirin
64071	Aspirin powder (J M Loveridge Ltd)	Aspirin
65027	Bisoprolol 5mg / Aspirin 100mg capsules	Aspirin
66345	Aspirin 75mg dispersible tablets (DE Pharmaceuticals)	Aspirin
66546	Aspirin 75mg dispersible tablets (Numark Ltd)	Aspirin
66563	Aspirin 75mg gastro-resistant tablets (Phoenix Healthcare Distribution Ltd)	Aspirin
66861	Aspirin 75mg effervescent tablets	Aspirin
67124	Bisoprolol 10mg / Aspirin 75mg capsules	Aspirin
67160	Aspirin 300mg dispersible tablets (Lloyds Pharmacy Ltd)	Aspirin
67362	Aspirin 300mg suppositories (Alliance Healthcare (Distribution) Ltd)	Aspirin
67521	Aspirin 15mg/5ml oral suspension	Aspirin
67754	Aspirin 300mg dispersible tablets (Almus Pharmaceuticals Ltd)	Aspirin
67858	Aspirin 25mg capsules	Aspirin
68051	Aspirin 150mg suppositories (Colorama Pharmaceuticals Ltd)	Aspirin
68752	Aspirin 75mg tablets (Sigma Pharmaceuticals Plc)	Aspirin
70549	Danamep 75mg dispersible tablets (Ecogen Europe Ltd)	Aspirin
70841	Aspirin 300mg Dispersible tablet (Family Health)	Aspirin
71078	Aspirin 300mg dispersible tablets (Mawdsley-Brooks & Company	Aspirin

For peer review only

	Ltd)	
71192	Aspirin 75mg tablets (Kent Pharmaceuticals Ltd)	Aspirin
489	Clopidogrel 75mg tablets	Clopidogrel
836	Plavix 75mg tablets (Sanofi)	Clopidogrel
17816	PLAVIX FC	Clopidogrel
17817	CLOPIDOGREL FC	Clopidogrel
38349	Clopidogrel 300mg tablets	Clopidogrel
38998	Plavix 300mg tablets (Sanofi)	Clopidogrel
40913	Grepid 75mg tablets (Kent Pharmaceuticals Ltd)	Clopidogrel
42750	Clopidogrel 75mg tablets (Actavis UK Ltd)	Clopidogrel
45905	Clopidogrel 1mg/ml oral suspension	Clopidogrel
46891	Clopidogrel 75mg/5ml oral suspension	Clopidogrel
52761	Clopidogrel 75mg tablets (Dr Reddy's Laboratories (UK) Ltd)	Clopidogrel
53751	Clopidogrel 75mg tablets (Phoenix Healthcare Distribution Ltd)	Clopidogrel
54700	Clopidogrel 75mg tablets (A A H Pharmaceuticals Ltd)	Clopidogrel
55161	Clopidogrel 75mg tablets (Wockhardt UK Ltd)	Clopidogrel
56807	Clopidogrel 75mg tablets (Teva UK Ltd)	Clopidogrel
57036	Clopidogrel 75mg tablets (Mylan)	Clopidogrel
58347	Clopidogrel 75mg tablets (DE Pharmaceuticals)	Clopidogrel
58448	Clopidogrel 75mg tablets (Aspire Pharma Ltd)	Clopidogrel
59904	Clopidogrel 75mg/5ml oral solution	Clopidogrel
62855	Clopidogrel 75mg tablets (Alliance Healthcare (Distribution) Ltd)	Clopidogrel
62978	Clopidogrel 75mg tablets (Sandoz Ltd)	Clopidogrel
63450	Clopidogrel 75mg tablets (Almus Pharmaceuticals Ltd)	Clopidogrel
65909	Clopidogrel 75mg tablets (Milpharm Ltd)	Clopidogrel
67037	Clopidogrel 75mg tablets (Zentiva)	Clopidogrel
39932	Prasugrel 10mg tablets	Prasugrel
40114	Prasugrel 5mg tablets	Prasugrel
40591	Efient 5mg tablets (Eli Lilly and Company Ltd)	Prasugrel
41229	Efient 10mg tablets (Eli Lilly and Company Ltd)	Prasugrel
45576	Ticagrelor 90mg tablets	Ticagrelor
47895	Brilique 90mg tablets (AstraZeneca UK Ltd)	Ticagrelor
66973	Ticagrelor 60mg tablets	Ticagrelor
68710	Brilique 60mg tablets (AstraZeneca UK Ltd)	Ticagrelor
70606	Ticagrelor 90mg orodispersible tablets sugar free	Ticagrelor

For peer review only

Anticoagulants (warfarin, dabigatran, rivaroxaban, apixaban)

Gemscript product code	Product name
45	Warfarin 1mg tablets
61	Warfarin 3mg tablets
833	Warfarin 3mg/5ml oral solution
1781	Warfarin 5mg tablets
2675	Fragmin 10,000units/4ml solution for injection ampoules (Pfizer Ltd)
2676	Fragmin 5,000units/0.2ml solution for injection pre-filled syringes (Pfizer Ltd)
2677	Clexane 100mg/ml Injection (Aventis Pharma)
3744	Heparin 10 iu/ml Flush solution
3895	Heparin sodium 1000iu/ml Injection
4446	Acenocoumarol 1mg tablets
4888	Heplok 10 iu/ml Oral solution (LEO Pharma)
4995	Enoxaparin 100mg/ml injection
5305	Sinthrome 1mg tablets (Merus Labs Luxco S.a R.L.)
5526	Fragmin 2,500units/0.2ml solution for injection pre-filled syringes (Pfizer Ltd)
5747	Fragmin 25,000iu/ml Solution for injection (Pfizer Ltd)
5998	Fragmin 10,000 iu/ml Solution for injection (Pfizer Ltd)
6262	Warfarin 500microgram tablets
6478	Enoxaparin sodium 20mg/0.2ml solution for injection pre-filled syringes
6695	Dalteparin sodium 2,500units/0.2ml solution for injection pre-filled syringes
6822	Elmiron 100mg capsules (Teva UK Ltd)
6860	Fragmin 15,000units/0.6ml solution for injection pre-filled syringes (Pfizer Ltd)
7154	Clexane Forte 120mg/0.8ml solution for injection pre-filled syringes (Sanofi)
7199	Enoxaparin sodium 40mg/0.4ml solution for injection pre-filled syringes
7307	Clexane 40mg/0.4ml solution for injection pre-filled syringes (Sanofi)
7371	Clexane 100mg/1ml solution for injection pre-filled syringes (Sanofi)
8466	Marevan 1mg tablets (AMCo)
8467	Marevan 3mg tablets (AMCo)
8664	Heparin sodium 5000iu/ml Injection
9140	Dalteparin sodium 10,000units/4ml solution for injection ampoules
9593	Dalteparin 25000iu/ml injection solution
9605	Dalteparin sodium 5,000units/0.2ml solution for injection pre-filled syringes
9610	Tinzaparin 20000 iu/ml Injection
9640	Tinzaparin 10000 IU/ml Injection
10002	Dalteparin 10000iu/1ml injection solution
10004	Clexane 80mg/0.8ml solution for injection pre-filled syringes (Sanofi)
10044	Dalteparin sodium 10,000units/0.4ml solution for injection pre-filled syringes
10072	Fragmin 10,000units/0.4ml solution for injection pre-filled syringes (Pfizer Ltd)
10170	Dalteparin sodium 15,000units/0.6ml solution for injection pre-filled syringes
10194	Dalteparin sodium 12,500units/0.5ml solution for injection pre-filled syringes

For peer review only

10240	Tinzaparin sodium 14,000units/0.7ml solution for injection pre-filled syringes
10532	Minihep calcium 5000iu/0.2ml Injection (LEO Pharma)
10533	Calciparine 25,000iu/ml Injection (Sanofi-Synthelabo Ltd)
10560	WARFARIN 10 MG TAB
11372	Heparin 100iu/ml Flush solution
12681	Heparin calcium 25,000iu/ml Injection
12974	Clexane 150mg/ml Injection (Aventis Pharma)
13058	Enoxaparin 150mg/ml injection
13097	Clexane 20mg/0.2ml solution for injection pre-filled syringes (Sanofi)
13210	Enoxaparin sodium 80mg/0.8ml solution for injection pre-filled syringes
13270	Enoxaparin sodium 120mg/0.8ml solution for injection pre-filled syringes
13348	Marevan 5mg tablets (AMCo)
13501	Dindevan 50mg Tablet (Goldshield Pharmaceuticals Ltd)
13502	Dindevan 10mg Tablet (Goldshield Pharmaceuticals Ltd)
13503	Phenindione 50mg tablets
13504	Phenindione 25mg tablets
13505	Phenindione 10mg tablets
13568	Heparin sodium 25,000iu/ml Subcutaneous injection
13644	Dindevan 25mg Tablet (Goldshield Pharmaceuticals Ltd)
13663	Innohep 20000 iu/ml Injection (LEO Pharma)
13716	Heparin sodium 25,000iu/ml Injection
14099	Clexane forte 150mg/ml Injection (Aventis Pharma)
14110	Tinzaparin sodium 10,000units/0.5ml solution for injection pre-filled syringes
14138	Enoxaparin sodium 60mg/0.6ml solution for injection pre-filled syringes
14212	Tinzaparin sodium 3,500units/0.35ml solution for injection pre-filled syringes
14308	Tinzaparin sodium 18,000units/0.9ml solution for injection pre-filled syringes
14341	Clexane Forte 150mg/1ml solution for injection pre-filled syringes (Sanofi)
14788	Innohep 10,000units/0.5ml solution for injection pre-filled syringes (LEO Pharma)
14794	Monoparin 1000iu/ml Injection (C P Pharmaceuticals Ltd)
14851	Tinzaparin sodium 4,500units/0.45ml solution for injection pre-filled syringes
14891	Dalteparin sodium 18,000units/0.72ml solution for injection pre-filled syringes
15006	Sinthrome 4mg Tablet (Alliance Pharmaceuticals Ltd)
15293	Heparin sodium 5000iu/ml pre-filled Injection
15376	Acenocoumarol 4mg tablets
15709	Tinzaparin 3500 IU/0.3ml Sterile solution
16061	Innohep 3,500units/0.35ml solution for injection pre-filled syringes (LEO Pharma)
16476	Fragmin 18,000units/0.72ml solution for injection pre-filled syringes (Pfizer Ltd)
16530	Fragmin 12,500units/0.5ml solution for injection pre-filled syringes (Pfizer Ltd)
17004	Tinzaparin sodium 20,000units/2ml solution for injection vials
17007	Tinzaparin sodium 2,500units/0.25ml solution for injection pre-filled syringes
17049	Innohep 18,000units/0.9ml solution for injection pre-filled syringes (LEO Pharma)
17484	Innohep 10,000 IU/ml Injection (LEO Pharma)
17592	Innohep 4,500units/0.45ml solution for injection pre-filled syringes (LEO Pharma)
17664	Clexane 60mg/0.6ml solution for injection pre-filled syringes (Sanofi)
17791	Innohep 5000iu/5ml Sterile solution (LEO Pharma)

For peer review only

17965	Marevan 500microgram tablets (AMCo)
18209	Fragmin 7,500units/0.3ml solution for injection pre-filled syringes (Pfizer Ltd)
18732	Innohep 3500 iu/0.3ml Sterile solution (LEO Pharma)
19280	Innohep 14,000units/0.7ml solution for injection pre-filled syringes (LEO Pharma)
19337	Multiparin 125,000units/5ml solution for injection vials (Wockhardt UK Ltd)
19486	Dalteparin sodium 7,500units/0.3ml solution for injection pre-filled syringes
19989	Tinzaparin sodium 40,000units/2ml solution for injection vials
20010	Uniparin calcium 25,000iu/ml Subcutaneous injection (C P Pharmaceuticals Ltd)
20024	Uniparin forte 10,000 iu/0.4ml Subcutaneous injection (C P Pharmaceuticals Ltd)
20028	Multiparin 5,000units/5ml solution for injection vials (Wockhardt UK Ltd)
20029	Multiparin 25,000units/5ml solution for injection vials (Wockhardt UK Ltd)
20153	Enoxaparin sodium 150mg/1ml solution for injection pre-filled syringes
20154	Enoxaparin sodium 100mg/1ml solution for injection pre-filled syringes
20411	Alphaparin 3000iu/0.5ml Injection (Grifols UK Ltd)
20754	WARFARIN
21233	Innohep 20,000units/2ml solution for injection vials (LEO Pharma)
21316	Innohep 40,000units/2ml solution for injection vials (LEO Pharma)
21365	Uniparin 5000iu/0.2ml Injection (C P Pharmaceuticals Ltd)
21490	Monoparin 5000iu/ml Injection (C P Pharmaceuticals Ltd)
21518	Monoparin 25,000iu/ml Injection (C P Pharmaceuticals Ltd)
22428	Dalteparin sodium 100,000units/4ml solution for injection vials
23078	Warfarin 1mg Tablet (WB Pharmaceuticals Ltd)
23570	Fondaparinux sodium 7.5mg/0.6ml solution for injection pre-filled syringes
23573	Fondaparinux sodium 5mg/0.4ml solution for injection pre-filled syringes
23579	Fondaparinux sodium 2.5mg/0.5ml solution for injection pre-filled syringes
24896	Heparin low molecular weight 2500 iu/0.2ml Sterile solution
25155	Fragmin 100,000units/4ml solution for injection vials (Pfizer Ltd)
25195	Heparin sodium 25,000iu/ml pre-filled Injection
25287	Unihep leo 1000unit/ml Injection (LEO Pharma)
26146	Heparin low molecular weight 10,000 iu/ml Sterile solution
27035	Pump-hep 1000unit/ml Infusion (LEO Pharma)
27139	Pentosan polysulfate sodium 100mg capsules
27325	Innohep 2,500units/0.25ml solution for injection pre-filled syringes (LEO Pharma)
28506	Heparin low molecular weight 3500 iu/0.3ml Sterile solution
28593	Heparin sodium 1000iu/ml pre-filled Injection
29043	Arixtra 2.5mg/0.5ml solution for injection pre-filled syringes (Aspen Pharma Trading Ltd)
29207	Innohep 5000iu/0.5ml Sterile solution (LEO Pharma)
29317	Tinzaparin 5000 i.u./0.5ml Sterile solution
29318	Heparin low molecular weight 2500 iu/ml Sterile solution
30108	Heparin calcium 5000iu/0.2ml Injection
30202	Warfarin wbp 1mg Tablet (Boehringer Ingelheim Ltd)
30203	Warfarin wbp 3mg Tablet (Boehringer Ingelheim Ltd)
30396	Unihep leo 5000iu/ml Injection (LEO Pharma)
31148	Flolan 500microgram powder and solvent (pH10.5) for solution for infusion vials

	(GlaxoSmithKline UK Ltd)
31511	Warfarin 3mg Tablet (WB Pharmaceuticals Ltd)
31937	Warfarin 5mg tablets (Teva UK Ltd)
32511	Tinzaparin 5000 i.u./5ml Sterile solution
32645	Heparin sodium 25,000iu/ml Injection
33307	Heparin sodium 5,000units/1ml solution for injection ampoules
33558	Monoparin calcium 5,000units/0.2ml solution for injection ampoules (Wockhardt UK Ltd)
33711	Warfarin 5mg Tablet (WB Pharmaceuticals Ltd)
34019	Warfarin 1mg tablets (IVAX Pharmaceuticals UK Ltd)
34086	Warfarin 3mg Tablet (Celltech Pharma Europe Ltd)
34087	Warfarin 1mg Tablet (Celltech Pharma Europe Ltd)
34088	Warfarin 5mg Tablet (Celltech Pharma Europe Ltd)
34095	Warfarin wbp 5mg Tablet (Boehringer Ingelheim Ltd)
34299	Warfarin 1mg tablets (Teva UK Ltd)
34416	Warfarin 1mg tablets (Kent Pharmaceuticals Ltd)
34417	Warfarin 3mg tablets (Teva UK Ltd)
34418	Warfarin 5mg tablets (Mylan)
34517	Warfarin 1mg tablets (Mylan)
34526	Warfarin 3mg tablets (Mylan)
34576	Warfarin 1mg Tablet (Lagap)
34691	Warfarin 5mg Tablet (Regent Laboratories Ltd)
34758	Warfarin 3mg tablets (IVAX Pharmaceuticals UK Ltd)
34864	Warfarin 5mg tablets (IVAX Pharmaceuticals UK Ltd)
34918	Warfarin 5mg tablets (Actavis UK Ltd)
35033	Heparin sodium 5,000units/5ml solution for injection vials
35941	Heparin sodium 5,000units/5ml solution for injection ampoules
36099	Warfarin 1mg/5ml oral suspension
36142	Heparin sodium 25,000units/1ml solution for injection ampoules
36172	Clexane 300mg/3ml solution for injection multidose vials (Sanofi)
36196	Heparin sodium 1,000units/1ml solution for injection ampoules
36911	Fragmin 10,000units/1ml solution for injection ampoules (Pfizer Ltd)
36989	Fragmin 10,000units/1ml solution for injection pre-filled syringes (Pfizer Ltd)
37086	Enoxaparin sodium 300mg/3ml solution for injection vials
37131	Heparin sodium 25,000units/5ml solution for injection vials
37613	Heparin sodium 10,000units/10ml solution for injection ampoules
37616	Heparin sodium 10 unit/ml Solution
37678	Heparin sodium 5,000units/0.2ml solution for injection ampoules
37704	Minihep 25,000iu/ml Subcutaneous preparation (LEO Pharma)
38041	Warfarin sodium 5mg/ml oral suspension
38044	Warfarin 5mg/5ml oral solution
38327	Arixtra 7.5mg/0.6ml solution for injection pre-filled syringes (Aspen Pharma Trading Ltd)
38536	Fondaparinux sodium 1.5mg/0.3ml solution for injection pre-filled syringes
38839	Arixtra 5mg/0.4ml solution for injection pre-filled syringes (Aspen Pharma Trading

For peer review only

	Ltd)
39119	Rivaroxaban 10mg tablets
39444	Dabigatran etexilate 110mg capsules
39503	Dabigatran etexilate 75mg capsules
39639	Xarelto 10mg tablets (Bayer Plc)
39755	Pradaxa 110mg capsules (Boehringer Ingelheim Ltd)
39866	Warfarin 1mg tablets (Almus Pharmaceuticals Ltd)
40143	Warfarin 500microgram tablets (A A H Pharmaceuticals Ltd)
40715	Heparin 100iu/ml Oral solution (LEO Pharma)
42106	Unihep leo 25,000iu/ml Injection (LEO Pharma)
42474	Pradaxa 75mg capsules (Boehringer Ingelheim Ltd)
42853	Heparin calcium 25,000iu/ml Injection
43407	Warfarin 3mg tablets (A A H Pharmaceuticals Ltd)
43408	Warfarin 1mg tablets (A A H Pharmaceuticals Ltd)
43409	Warfarin 5mg tablets (A A H Pharmaceuticals Ltd)
43655	Warfarin sodium oral solution
44238	Heparin 50 iu/5ml Flush solution (Wockhardt UK Ltd)
44491	Heparin sodium 125,000units/5ml solution for injection vials
44866	Warfarin sodium 1mg/ml oral supension SF
45597	Lepirudin 50mg powder for solution for injection vials
45911	Arixtra 1.5mg/0.3ml solution for injection pre-filled syringes (Aspen Pharma Trading Ltd)
46632	Dabigatran etexilate 150mg capsules
46678	Pradaxa 150mg capsules (Boehringer Ingelheim Ltd)
46924	Phenindione 10mg tablets (AMCo)
47207	Rivaroxaban 20mg tablets
47353	Rivaroxaban 15mg tablets
47397	Heparin sodium 25,000units/5ml solution for injection ampoules
47566	Apixaban 2.5mg tablets
47925	Xarelto 20mg tablets (Bayer Plc)
47944	Warfarin 1mg tablets (Actavis UK Ltd)
48070	Warfarin sodium tablets
48134	Xarelto 15mg tablets (Bayer Plc)
48673	Dalteparin sodium 10,000units/1ml solution for injection ampoules
48869	Warfarin 1mg/ml oral suspension sugar free
48966	Rivaroxaban 15mg tablets
49578	Dalteparin sodium 10,000units/1ml solution for injection pre-filled syringes
50000	Warfarin 1mg/ml oral suspension sugar free (A A H Pharmaceuticals Ltd)
50391	Fragmin 18,000units/0.72ml solution for injection pre-filled syringes (Waymade Healthcare Plc)
50994	Heparin sodium 500units/500ml infusion bags
51006	Clexane 80mg/0.8ml solution for injection pre-filled syringes (DE Pharmaceuticals)
51350	Fragmin 15,000units/0.6ml solution for injection pre-filled syringes (Waymade Healthcare Plc)
51484	Warfarin 1mg tablets (Bristol Laboratories Ltd)

For peer review only

51496	Warfarin 1mg tablets (Phoenix Healthcare Distribution Ltd)
51509	Warfarin 1mg tablets (APC Pharmaceuticals & Chemicals (Europe) Ltd)
51642	Clexane 100mg/1ml solution for injection pre-filled syringes (Lexon (UK) Ltd)
52004	Fragmin 12,500units/0.5ml solution for injection pre-filled syringes (Waymade Healthcare Plc)
52841	Heparin calcium 5,000units/0.2ml solution for injection ampoules
53350	Heparin sodium 1,000units/500ml infusion bags
53740	Eliquis 2.5mg tablets (Bristol-Myers Squibb Pharmaceuticals Ltd)
53745	Warfarin 3mg tablets (Bristol Laboratories Ltd)
53752	Warfarin 1mg tablets (Alliance Healthcare (Distribution) Ltd)
54066	Apixaban 5mg tablets
54234	Heparin sodium 1,000units/500ml infusion Viaflex bags (Baxter Healthcare Ltd)
54451	Rivaroxaban 20mg tablets
54892	Warfarin 1mg/ml oral suspension sugar free (Alliance Healthcare (Distribution) Ltd)
54927	Heparin sodium 2,000units/1litre infusion bags
54946	Warfarin 3mg tablets (Actavis UK Ltd)
55096	Fragmin 5,000units/0.2ml solution for injection pre-filled syringes (Waymade Healthcare Plc)
55316	Warfarin 3mg/5ml oral suspension
55490	Heparin sodium 10,000 iu/ml Injection
55565	Clexane 100mg/1ml solution for injection pre-filled syringes (DE Pharmaceuticals)
55577	Sinthrome 1mg tablets (Lexon (UK) Ltd)
55604	Orgaran 750units/0.6ml solution for injection ampoules (Aspen Pharma Trading Ltd)
56166	Heparin sodium 100units/1ml solution for injection ampoules
56289	Xarelto 20mg tablets (Bayer Plc)
56314	Warfarin 3mg tablets (Kent Pharmaceuticals Ltd)
56315	Anticoagulant Citrate Dextrose Solution formula A infusion 500ml bags
56398	Fragmin 5,000units/0.2ml solution for injection pre-filled syringes (Mawdsley-Brooks & Company Ltd)
56640	Xarelto 15mg tablets (Bayer Plc)
57032	Warfarin 1mg/ml oral suspension sugar free (Rosemont Pharmaceuticals Ltd)
58519	Warfarin 1mg tablets (DE Pharmaceuticals)
58594	Eliquis 5mg tablets (Bristol-Myers Squibb Pharmaceuticals Ltd)
58787	Warfarin 5mg tablets (Alliance Healthcare (Distribution) Ltd)
58962	Warfarin 3mg tablets (DE Pharmaceuticals)
59400	Warfarin 500microgram tablets (Sigma Pharmaceuticals Plc)
59578	Warfarin 3mg tablets (Phoenix Healthcare Distribution Ltd)
59761	Heparin sodium 1,000units/1ml solution for injection ampoules (Wockhardt UK Ltd)
60041	Danaparoid sodium 750units/0.6ml solution for injection ampoules
60188	Heparin sodium 5,000units/1litre infusion bags
60589	Warfarin 500microgram tablets (Actavis UK Ltd)
60949	Warfarin 5mg/5ml oral suspension
61949	Fondaparinux sodium 10mg/0.8ml solution for injection pre-filled syringes
62150	Rivaroxaban 2.5mg tablets
62309	Warfarin 500microgram tablets (Kent Pharmaceuticals Ltd)

For peer review only

62310	Warfarin 500microgram tablets (AMCo)
62856	Tinzaparin sodium 12,000units/0.6ml solution for injection pre-filled syringes
62902	Tinzaparin sodium 16,000units/0.8ml solution for injection pre-filled syringes
62959	Heparin calcium 5,000units/0.2ml solution for injection ampoules (A A H Pharmaceuticals Ltd)
63071	Warfarin 4mg tablets
63101	Tinzaparin sodium 8,000units/0.4ml solution for injection pre-filled syringes
63146	Heparin sodium 20,000units/20ml solution for injection ampoules
63169	Innohep 12,000units/0.6ml solution for injection pre-filled syringes (LEO Pharma)
63297	Heparin sodium 5,000units/5ml solution for injection vials (LEO Pharma)
63440	Epoprostenol 500microgram powder and solvent (pH10.5) for solution for infusion vials
63571	Innohep 16,000units/0.8ml solution for injection pre-filled syringes (LEO Pharma)
64133	Heparin sodium 5,000units/0.2ml solution for injection ampoules (A A H Pharmaceuticals Ltd)
64315	Anticoagulant solution ACD-A 500ml bags (Haemonetics Ltd)
64500	Xarelto 2.5mg tablets (Bayer Plc)
64559	Heparin sodium 1,000units/1ml solution for injection ampoules (A A H Pharmaceuticals Ltd)
64581	Innohep 8,000units/0.4ml solution for injection pre-filled syringes (LEO Pharma)
64678	Edoxaban 60mg tablets
64969	Clexane 20mg/0.2ml solution for injection pre-filled syringes (Sigma Pharmaceuticals Plc)
64998	Epoprostenol 1.5mg powder and solvent (pH10.5) for solution for infusion vials
65247	Edoxaban 30mg tablets
65285	Warfarin 1mg tablets (Crescent Pharma Ltd)
65496	Warfarin 500microgram tablets (Phoenix Healthcare Distribution Ltd)
65538	Elmiron 100mg capsules (Imported (United States))
65746	Warfarin 500microgram tablets (DE Pharmaceuticals)
65850	Lixiana 60mg tablets (Daiichi Sankyo UK Ltd)
65876	Edoxaban 15mg tablets
66286	Warfarin 2.5mg/5ml oral solution
66529	Lixiana 30mg tablets (Daiichi Sankyo UK Ltd)
66570	Warfarin 1mg tablets (Waymade Healthcare Plc)
68591	Warfarin 500microgram tablets (Alliance Healthcare (Distribution) Ltd)
68667	Warfarin 5mg capsules
68795	Warfarin 1mg capsules
69128	Warfarin 500micrograms/5ml oral solution
69194	Heparin low molecular weight 5000iu/0.2ml Sterile solution
70831	Phenindione 50mg tablets (AMCo)
70866	Inhixa 40mg/0.4ml solution for injection pre-filled syringes (Techdow Pharma England Ltd)
71132	Clexane 20mg/0.2ml solution for injection pre-filled syringes (DE Pharmaceuticals)
71196	Warfarin 1.5mg/5ml oral solution
71274	Inhixa 60mg/0.6ml solution for injection pre-filled syringes (Techdow Pharma England Ltd)

For peer review only

71303	Rivaroxaban 15mg tablets and Rivaroxaban 20mg tablets
71386	Warfarin 1mg/5ml oral solution (Special Order)

For peer review only
