# Supplementary material for: Comprehensive ascertainment of bleeding in patients prescribed different combinations of dual antiplatelet therapy (DAPT) and triple therapy (TT) in the UK: study protocol for three population-based cohort studies emulating ‘target trials’ (the ADAPTT Study)
Source: BMJ Open. 2019 Jun 4;9(6):e029388. doi: 10.1136/bmjopen-2019-029388 (PMC6561407; doi:10.1136/bmjopen-2019-029388)
Supplement: Reviewer comments [file bmjopen-2019-029388.reviewer_comments.pdf]

PEER REVIEW HISTORY

BMJ Open publishes all reviews undertaken for accepted manuscripts. Reviewers are asked to complete a checklist review form (**http://bmjopen.bmj.com/site/about/resources/checklist.pdf**) and are provided with free text boxes to elaborate on their assessment. These free text comments are reproduced below.

ARTICLE DETAILS

**TITLE (PROVISIONAL)**	Comprehensive ascertainment of bleeding in patients prescribed different combinations of dual antiplatelet therapy (DAPT) and triple therapy (TT) in the UK: study protocol for three population-based cohort studies emulating 'target trials' (the ADAPTT Study)
**AUTHORS**	Pufulete, Maria; Harris, Jessica; Sterne, Jonathan; Johnson, Thomas; Lasserson, Daniel; Mumford, Andrew; Doble, Brett; Wordsworth, Sarah; Benedetto, Umberto; Rogers, Chris; Loke, Yoon; Pithara, Christalla; Redwood, Sabi; Reeves, Barnaby

This article was not externally reviewed at BMJ Open. Protocols that have been independently assessed prior to submission to BMJ Open are usually fast-tracked to publication on the grounds that further substantial changes will not be possible. This independent assessment will usually be external, independent review for both a substantial grant award from a non-commercial or government funder and ethics approval.
